# Integration of HIV and sexual and reproductive health in the era of anti-retroviral-based prevention: findings from assessments in Kenya, Malawi and Zimbabwe

**DOI:** 10.12688/gatesopenres.13330.1

**Published:** 2021-09-15

**Authors:** Fannie Kachale, Imelda Mahaka, Fatima Mhuriro, Mary Mugambi, Joseph Murungu, Barbra Ncube, Getrude Ncube, Albert Ndwiga, Rose Nyirenda, Violet Otindo, Anna Carter, Megan Dunbar, Janet Fleischman, Jessica Rodrigues, Kate Segal

**Affiliations:** 1 Department of Reproductive Health, Ministry of Health, Lilongwe, Malawi; 2 Pangaea Zimbabwe AIDS Trust (PZAT), Harare, Zimbabwe; 3 Reproductive Health Unit, Ministry of Health and Child Care, Harare, Zimbabwe; 4 National AIDS & STI Control Programme (NASCOP), Ministry of Health, Nairobi, Kenya; 5 Department of AIDS & TB, Ministry of Health and Child Care, Harare, Zimbabwe; 6 Department of Family Health, Ministry of Health, Nairobi, Kenya; 7 Department of HIV/AIDS, Ministry of Health, Lilongwe, Malawi; 8 Georgetown University Center for Innovation in Global Health, Washington, DC, USA; 9 Consultant, AVAC, New York, USA; 10 Consultant, Georgetown University Center for Innovation in Global Health, Washington, DC, USA; 11 AVAC, New York, USA

**Keywords:** HIV prevention, sexual and reproductive health, integration, oral pre-exposure prophylaxis, family planning, adolescent girls and young women

## Abstract

**Background: **Though substantial progress has been made to curb the HIV epidemic, high rates of new HIV infections persist among adolescent girls and young women (AGYW) in sub-Saharan Africa, reflecting critical gaps in reaching them with integrated HIV prevention and sexual and reproductive health (SRH) services. With the scale-up of oral pre-exposure prophylaxis (PrEP) and multiple novel HIV prevention products on the horizon, countries have a unique opportunity to expand innovative approaches to deliver comprehensive, integrated HIV/SRH services.

**Methods: **This article is a comparative analysis of findings from rapid landscaping analyses in Kenya, Malawi and Zimbabwe to highlight cross-country trends and context-specific realities around HIV/SRH integration. The analyses in Kenya and Zimbabwe were completed by Ministries of Health (MOH) and the HIV Prevention Market Manager project and include 20 health facility assessments, 73 key informant interviews and six community dialogues. In Malawi, the analysis was completed by the MOH and Georgetown University Center for Innovation in Global Health and includes 70 key informant interviews and a review of national policies and program implementation in Blantyre. Findings were validated through a review of literature and policies in each country.

**Results: **The policy environment in all three countries is conducive to HIV/SRH integration, though operationalization continues to present challenges, with most policies preceding and not accounting for oral PrEP rollout. National coordination mechanisms, youth-friendly health services and prevention of mother-to-child transmission programs are promising practices, while siloed and resource-constrained health systems, limited provider capacity, lack of support for demand generation and structural factors exacerbate barriers to achieving integration.

**Conclusions: **As new HIV prevention products are introduced, demand for integrated HIV/SRH services is likely to grow. Investing in HIV/SRH integration can help to ensure sustainable, government-led responses to the HIV epidemic, streamline service delivery and improve the health outcomes and lives of AGYW.

## Introduction

Though substantial progress has been made to curb the HIV epidemic over the past decade, high rates of new HIV infections persist, especially among adolescent girls and young women (AGYW) in sub-Saharan Africa (SSA), who in 2019 had approximately three times the HIV incidence rate of their male counterparts. ^1^ To meet global HIV prevention goals and improve health outcomes, prevention programs need to reach AGYW. The 2019 results of the Evidence for Contraceptive Options in HIV Outcomes study (ECHO) underscored a critical gap in targeting AGYW with integrated HIV prevention and sexual and reproductive health (SRH) services. ^2^ The SARS-CoV-2 (COVID-19) pandemic has amplified the urgency for integration, as dire social and economic impacts heightened AGYW risk of HIV and STI infection and unintended pregnancy, disrupted critical HIV and SRH services, and further constrained health systems and healthcare workers in many low- and middle-income settings. With the scale up of oral pre-exposure prophylaxis (PrEP) and introduction of multiple novel HIV prevention products on the horizon, health systems are at a turning point, with an opportunity to expand new and innovative approaches to reach AGYW with comprehensive HIV/SRH services, including expanding access to existing and future PrEP products.

This article analyzes the progress that Kenya, Malawi and Zimbabwe have made towards the integration of HIV prevention and SRH (HIV/SRH integration). Examples from each country highlight promising approaches to HIV/SRH integration, identifying opportunities for improved service delivery and pinpointing persistent gaps that effectively limit the impact of high-potential interventions. By examining cross-country experiences, this article aims to highlight regional trends and context-specific realities around advancing HIV/SRH integration. Lessons can inform the scale-up of HIV/SRH integration in other high HIV-burden countries, though increased government and donor investment and political support will be required. 

### HIV prevention and SRH trends and landscape

**Figure 1.**
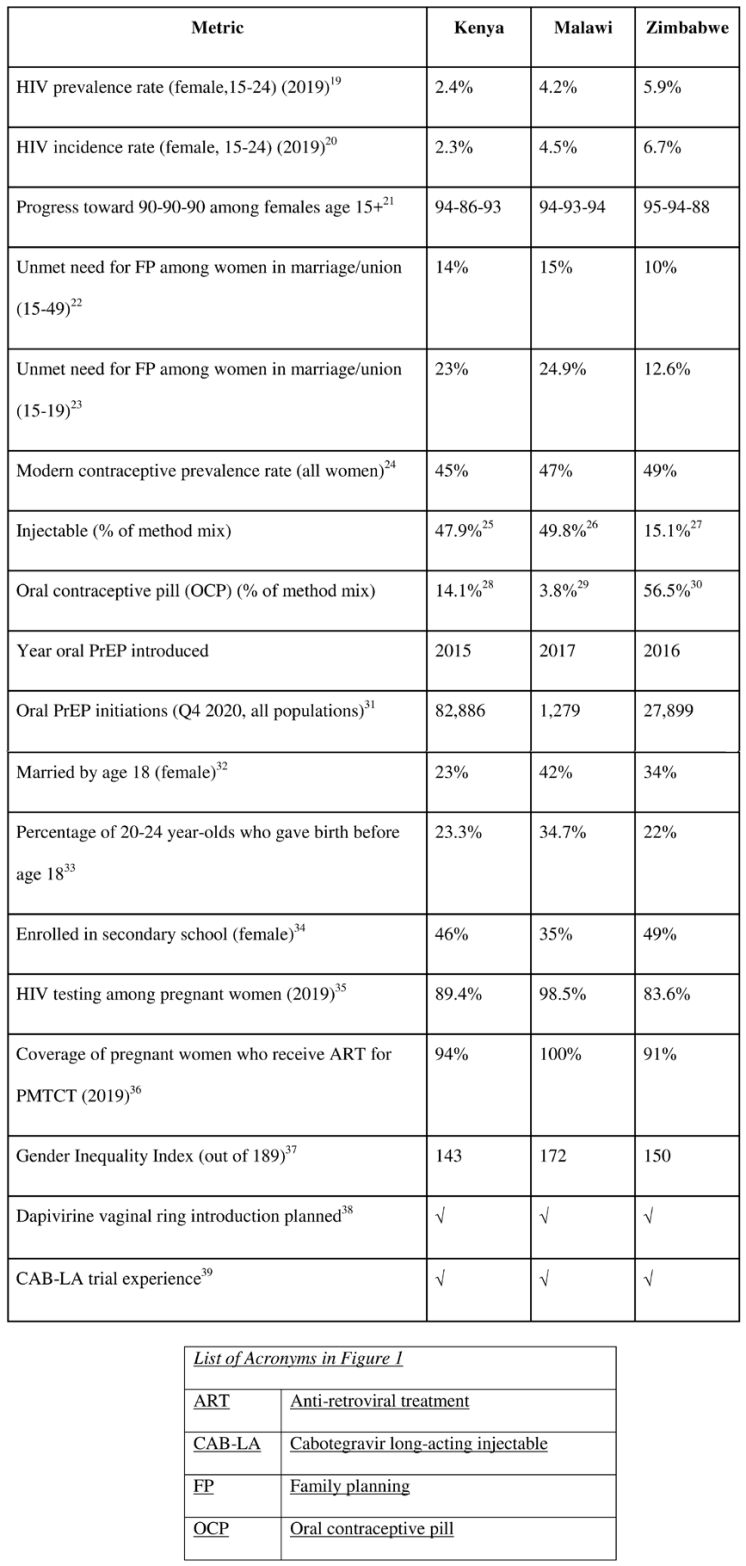
Comparison of demographic and epidemiological data in Kenya, Malawi and Zimbabwe.

Across countries, data reveal high HIV prevalence among young women, with especially high rates in Zimbabwe and Malawi (see Figure 1). ^3^ Data show significant strides in HIV testing among pregnant women, prevention of mother-to-child transmission (PMTCT) coverage and relatively high modern contraceptive prevalence rates for all women, pointing to SRH and maternal child health (MCH) services as strong entry points for integrated services. ^4, 5^ Unmet need for family planning (FP) is higher in Kenya and Malawi than in Zimbabwe, and the contraceptive method mix in Kenya and Malawi skews toward injectables, while in Zimbabwe, the oral contraceptive pill is the most common method. ^6– 9^ Kenya was the first of these countries to introduce PrEP and is furthest ahead with scale-up, while Malawi’s program is just beginning to roll out. ^10^

Across countries, contraception and PrEP are primarily accessed in public facilities, though the private sector, including healthcare providers in private practice, non-governmental organizations (NGOs) and faith-based clinics and pharmacies, are an important source of FP services, pointing to opportunities for integration with oral PrEP. In Kenya, 60% of contraceptives are obtained in the public sector and 34% in the private sector, while oral PrEP is delivered primarily in HIV clinics (57%) and safe spaces (25%, many NGO-run). ^11, 12^ Similarly, in Zimbabwe, 73% of contraceptives are obtained in the public sector and 22% via the private sector; oral PrEP is primarily obtained in public HIV clinics. ^13^ In Malawi, 79% of contraceptives are accessed in public facilities, 8% from Banja la Mtsogolo (BLM), the local Marie Stopes affiliate, and 6% from the private sector. ^14^

High HIV prevalence and incidence among AGYW, and the large percentage of the latter who gave birth before age 18, signal the need to better reach this population with HIV prevention and SRH information and services. High rates of early marriage in Malawi and low rates of secondary school enrolment for girls in Malawi and Zimbabwe underscore that structural and social barriers often undermine health outcomes. Limited data on AGYW points to a need for sex- and age-disaggregated data to better tailor programs. While the unmet need for FP among women of reproductive age ranges from 10% in Zimbabwe to 15% in Malawi, this figure is higher among AGYW (ranging from 12.6% in Zimbabwe to 24.9% in Malawi). Unmet need for FP among women living with HIV has been shown to be slightly lower than among women in the general population, ^15, 16^ though studies cite missed opportunities to provide FP through anti-retroviral treatment (ART) clinics as a contributing factor to the ongoing unmet need within this population. ^17, 18^

## Methods

This article is a comparative analysis of findings from three completed rapid landscaping analyses in Kenya, Malawi and Zimbabwe, to understand the current state of HIV/SRH integration in each country and to elucidate cross-country trends. The analyses collected programmatic data and insights to inform national programs. While original data from key informant interviews and health facility assessments are not contained in this article, because the analyses form the basis of this article, their methodologies are elaborated upon below.

### Research teams

In Kenya ^40^ and Zimbabwe, ^41^ the landscaping analyses were completed in 2020 by multi-disciplinary teams from the Ministries of Health (MOH) in each country and the HIV Prevention Market Manager project (PMM). Research teams identified sites, settings and key informants, developed data collection tools and carried out data collection and analysis. In Kenya, the research team was comprised of representatives from the National AIDS and STI Control Programme (NASCOP) and the Department of Family Health in Kenya and AVAC. In Zimbabwe, the research team was comprised of representatives from the Department of AIDS & TB at the Ministry of Health and Child Care (MoHCC), Pangaea Zimbabwe AIDS Trust (PZAT) and AVAC. Advocates in each country organized and led community dialogues with AGYW and, in Zimbabwe, organizations working with AGYW.

In Malawi, ^42^ the landscaping analysis was completed in 2020 by representatives from the Department of HIV/AIDS and Department of Reproductive Health at the MOH and Georgetown University Center for Innovation in Global Health. The analysis was conducted to inform the Blantyre Prevention Strategy, launched in May 2020 to catalyze local development of an innovative and data-driven HIV prevention delivery system at the district level. The district of Blantyre was chosen as the initial geography, due to the seemingly intractable HIV epidemic and continuing high numbers of new infections there. The research team conducted a literature review, identified government documents and key informants, conducted interviews and data collection, and analyzed findings.

### Data collection and analysis

In Kenya and Zimbabwe, research teams identified a cross-section of health facilities (public/private/youth-friendly (YF), high/low volume), settings (rural/urban) and key informants to provide a representative snapshot of HIV/SRH integration at country level. In total, 73 semi-structured, one-hour interviews were conducted with national, provincial, county and district-level MOH representatives; frontline HIV, SRH and MCH healthcare providers; implementing partners and donors. Six one-day community dialogues were held in-person with AGYW and NGOs. Twenty health facility assessments were conducted in five regions in Kenya and four provinces in Zimbabwe. Interviews were requested via email or telephone and health facility visits by letter or email, per MOH protocols. Interviews and facility assessments were primarily conducted in-person and virtually where not feasible, including due to COVID-19 restrictions.

Interviews were recorded and notes taken simultaneously, and facility assessment data were logged in a spreadsheet. To understand opportunities and barriers to HIV/SRH integration, qualitative interview findings were grouped by stakeholder perspectives and recurring themes identified at the government, health facility and community levels. Health facility assessments collected quantitative data on HIV, FP/SRH and YF services available (e.g., methods and hours offered, whether and how services are integrated, commodity stock-outs, YF accommodations) and staffing and general infrastructure (e.g., facility size, number and cadres of staff across HIV, SRH and YF service areas) to analyze the level of HIV/SRH integration and YF service delivery at each facility. PMM standardized interview guides, health facility assessment checklists and analysis of data across the two countries.

In Malawi, the research team conducted a review of national policies and implementation of HIV and SRH programs in the southern district of Blantyre and approximately 70 semi-structured interviews with key informants, which included: Blantyre district health officials; representatives from national and international NGOs, advocacy organizations, and implementing partners working on HIV, SRH and AGYW; healthcare providers at district health facilities; representatives from community-based organizations focused on women’s health and AGYW; organizations associated with the Blantyre Prevention Strategy; officials from U.S. government agencies in Malawi and from other key donors; and DREAMS ambassadors and AGYW peer educators. Interviews were conducted by WhatsApp, Zoom, telephone and email. Participants were recruited through the network of organizations and district and national officials associated with the Blantyre Prevention Strategy, local and international NGOs and researchers and community-based organizations with whom they worked. The research team standardized interview protocols and analysis of data.

To understand the spectrum of services in the Blantyre district, the research team analyzed the HIV and SRH information and services offered through public sector facilities and through youth-friendly health services (YFHS). It then examined services in the private sector, including national and international NGOs and those NGOs working through the U.S.-led DREAMS initiative. In addition, this analysis highlighted social and structural barriers for sustained and effective use of prevention interventions, including through the education system and through gender-based violence (GBV) programs, and the new and evolving challenges presented by the COVID-19 pandemic.

Standard informed consent procedures were followed and oral informed consent was sought and obtained by respective research team members in each country before interviews were conducted (MOH and AVAC in Kenya; MOH and PZAT in Zimbabwe; MOH and Georgetown University Center for Innovation in Global Health in Malawi). Interview transcripts, notes, recordings and health facility assessments were only accessible by the research teams.

The three assessments were comparatively analyzed in this article by findings on policies and enabling environments, health systems, service delivery and gaps to implementation of HIV/SRH integration. Findings were validated through a review of literature and policies in each country.

### Ethics approval

No ethics approval was sought for this article, as it analyzes the aggregate findings from completed assessments and did not entail additional data collection.

## Results

In this article, we grouped results according to: Enabling Environment and Policy Implementation; Health Systems; Service Delivery and Key Gaps. We highlighted promising national coordination mechanisms, health systems considerations and service delivery approaches to improve HIV/SRH integration, and summarized persistent barriers across countries.

### Enabling environment and policy implementation

Our analyses revealed that the policy environment in all three countries is conducive to expanded integration of HIV prevention and SRH, though most policies on integration preceded oral PrEP rollout (and therefore do not address its integration into SRH services). Operationalizing policies, however, continues to present challenges, especially in reaching AGYW with YFHS and safe spaces, and in translating policies to local/district levels.

While strong policies provide the framework for implementation, dedicated structures are required to support coordination of implementation of policies throughout the health system. Sub-national MOH departments for HIV and FP/reproductive health (RH) are critical for cascading operational changes down to facilities, yet they often work in silos, resulting in parallel budgets, workplans and M&E systems. One county RH coordinator in Kenya likened it to *“operat[ing] like water and oil, but this is changing.”* ^43^ Collaboration across local HIV and FP/RH departments has led to the formation of mechanisms to implement integration. Structures to support this varied across the countries included in this analysis.

Prior to the assessment, Kenya had separate mechanisms supporting the rollout of SRH and HIV services at national and sub-national levels. ^44^ In response to early assessment findings, the MOH in Kenya built on these existing structures to join HIV prevention and SRH leaders to renew commitment to integration: Kenya’s National AIDS and STI Control Programme (NASCOP) and Department of Family Health formed a national HIV/SRH integration sub-committee in 2020. The sub-committee analyzed 16 HIV/SRH policies and consulted stakeholders in all 47 counties in Kenya and, based on findings that most policies preceded oral PrEP and required updating to be more comprehensive, produced a policy circular on HIV/SRH integration containing concise directives for county officials and health facilities, and a package of resources to aid implementation. ^45^ Integration pilots are being planned in 2021 in five counties.

In Zimbabwe, close coordination across HIV and SRH leadership has historically been a strength. Government-led, program-specific (e.g., PrEP) technical working groups (TWGs) are integrated, as are the Prevention Partnership and Adolescent Reproductive and Sexual Health Partnership Forums. ^46^ Importantly, these platforms include representation from implementing partners and potential beneficiaries, including AGYW.

In Malawi, until recently, separate TWGs at the City and the District level resulted in fragmented programs, with City Councils responsible for urban catchment areas and District Councils responsible for rural ones, with the absence of a forum for routine involvement of community members, including AGYW and other beneficiaries. While Malawi has a national-level SRHR and HIV and AIDS integration sub-committee, the establishment of joint City and District TWGs with routine community engagement presents an opportunity to coordinate effective integrated services at the district-level. There was broad agreement among key informants in Malawi interviewed for the assessment that district-level leadership is key to operationalizing national integration policies, ^47^ consistent with findings in Kenya and Zimbabwe.

*“Coordination is a big challenge… The districts are eager to understand and provide input to the programs that are ongoing, including how targets are set for partners in their district, and they want to be in the lead in coordinating and directing projects and funding, but they lack adequate staffing and bandwidth. With limited time and human resource capacity, it’s difficult for them to engage as much as they might like in supervising partner activities.” -USAID/Malawi official* ^48^
*“Whatever model, we need to strengthen the DHO [District Health Office] – it’s the unit to ensure continuity. Supervision coupled with strengthening the relevant DHO office is what’s needed for the long run.” -University of Malawi, College of Medicine, professor* ^49^

## Health systems

In spite of promising approaches and models showing successful examples of HIV prevention and SRH integration (see Service Delivery section), siloed and resource-constrained health systems exacerbate barriers to achieving integration.

### Siloed delivery, M&E and supply chain systems

Across countries, HIV and SRH programs are funded, managed and delivered vertically through separate HIV and FP/RH departments within the MOH and delivered in separate physical spaces by different providers in facilities, and referrals are commonly used to fill gaps in services offered.

*“There is a tendency at facility level to look at HIV services as quite involving. As such, there is a tendency to push and refer, rather than to embrace integration. At the same time, in CCCs [comprehensive care clinics] we are already overwhelmed and yet we are asked to do family planning.” -County AIDS and STI Coordinator (CASCO), Kenya* ^50^
*“For young people it is even harder. You know how difficult it is to be shuffled from one place to another for services. It is disturbing coming to seek reproductive health service[s] then you have to explain your issue over and over to different nurses. It is embarrassing. So they leave without accessing the service. We have missed opportunities.” -MoHCC official, Zimbabwe* ^51^
*“If we are providing PrEP as a hospital but they have to go to OI [Opportunistic Infection, or HIV clinic] to get, it becomes a barrier. You tell them please go to OI, and you give them directions, then you call OI to check if the client has arrived; they don’t get there.” -YF provider, Zimbabwe* ^52^

Our assessments and other studies have found that this siloed service delivery cascade creates barriers to uptake, exacerbates stigma and does not respond to the priorities or multiple vulnerabilities faced by AGYW. ^53– 56^ This system is largely entrenched due to international donor support for a significant portion of these programs, requiring target setting, provider training and staffing, and reporting for HIV and SRH to be done separately. Moreover, there is a large imbalance in funding, where HIV is exponentially better-resourced than other SRH services. In Kenya, for instance, few counties have FP-specific budget lines and external donor investments in FP have decreased over time. ^57^

*“Sometimes the investments of partner-funded programs can lead to less integration. There is this tendency like … me I work with [an implementing partner], I am supposed to do HIV preventive services. MCH is none of my business.” -MCH nurse, Kenya* ^58^
*“There will be a funder who will fund HIV prevention, but won’t fund family planning or reproductive health activities. Or there will be a funder who will fund reproductive health and leave behind issues to do with HIV. For instance, the funder may say I won’t buy test kits for HIV but I will buy contraceptives for young people*. *” -MoHCC official, Zimbabwe* ^59^

As a result, data systems for monitoring and evaluation (M&E) and supply chain management are generally not integrated. ^60^ At facility level, HIV and SRH data are collected at different delivery areas and through separate registers, though providers emphasized that integrating M&E could be an impetus for delivering integrated services *(“if it is not reportable, then it is [perceived by providers to be] unimportant”*). ^61, 62^ Though some indicators may be shared across registers (e.g., Malawi has an integrated register for Antenatal Care and Maternity Delivery, which captures HIV information), patient tracking and monitoring remains unintegrated across sites. Furthermore, much of the data collected on HIV testing and treatment or prevention services, including oral PrEP, and specific contraceptive methods, are not always available by specific age bands, target populations or service areas, and data completeness and quality are ongoing challenges. ^63, 64^ As one implementing partner in Malawi noted, *“We’re missing out on a lot of data on the SRH entry point, since they [facilities] don’t have to report on those indicators. We’re not capturing how many we’re reaching and what the issues are.”* ^65^

In addition, conducting analyses of FP and HIV/PrEP data for program-monitoring or decision-making requires special analysis, though strategic information officers at MOH-level are typically siloed. Those working in HIV departments cannot readily access FP data and vice versa, and mechanisms in DHIS-2 (e.g., built-in pivot tables and standard reporting functions) do not support integrated analyses. There is a need to revise and strengthen M&E systems to document the provision of HIV services in SRH service areas and vice versa, and to integrate systems up the chain to national level, to enhance the ability to look at data in an integrated fashion. Electronic monitoring systems, currently being rolled out in Kenya, Malawi and Zimbabwe, provide an opportunity to track individual client health records across services and facilities. Data should be made available in real time and disaggregated by age to improve targeting of at-risk AGYW for evidence-based decision-making. Furthermore, M&E systems should be revised to incorporate new indicators on HIV/SRH integration as defined by governments, such as identifying at-risk AGYW through the presence of STIs and early or unintended pregnancy. ^66^

Bi-directional integration of data systems can support the integrated management of HIV and FP commodities; however, siloed supply chain systems for HIV and SRH commodities supporting public sector facilities and pharmacies are also a barrier to integration which needs to be addressed. ^67^ For example, PEPFAR, the largest HIV donor, currently does not allow funds to be used to procure contraceptive commodities other than condoms. Renewed donor efforts are needed to address these health system challenges to integration in order to move toward the provision of integrated services. 

### Healthcare provider capacity to offer integrated services to AGYW

Across countries and studies, healthcare provider capacity-building is underscored as the most critical need for integrating HIV prevention services, including HIV testing and PrEP, in SRH service delivery points. ^68– 70^ Generally, HIV healthcare providers are trained through pre-service education in SRH/FP, but SRH/FP providers lack similar training in HIV services and require training to test for HIV and screen for and provide oral PrEP.

Task-shifting for HIV testing and oral PrEP delivery could expand opportunities to offer combination prevention alongside SRH services. Similarly, task-shifting for contraceptive commodities, including for long-acting reversible contraceptives (LARCs) and for the self-injectable contraceptive, subcutaneous depot medroxyprogesterone acetate (DMPA-SC), could broaden access for at-risk women and girls. Currently, oral PrEP is generally delivered by ART nurses and staff specifically certified to prescribe PrEP. Task-shifting for PrEP counseling is prevalent in Zimbabwe, where lay providers can counsel on PrEP after completing PrEP-specific trainings, a model other countries can follow. ^71^ Diffusing the capacity to provide services across staff in this way could be perceived as alleviating nurses’ workloads, while offering a more comprehensive HIV/SRH package. A systematic review of evidence from low- and middle-income countries found task-shifting led to cost savings and efficiency gains across settings and health areas. ^72^

Task-shifting FP to community-based delivery is endorsed in all three countries, and far more advanced compared to oral PrEP. It has contributed to notable increases in contraceptive use in all three countries and in referrals for HIV testing and HIV/STI-related referrals, and increased dual prevention methods for women living with HIV. ^73, 74^ In Zimbabwe, community health workers (CHWs) can be key to creating demand among AGYW, with CHWs discretely supplying FP methods in rural communities. ^75^ In Malawi, health surveillance assistants (HSAs) provide integrated service delivery at the community level, while community-based distribution agents (CBDAs) provide information and distribute basic commodities to young people in communities and provide referrals to facilities for other services. ^76, 77^ Linkages between services in the community and health facilities are often weak for AGYW, indicating a need to expand and integrate the services that CHWs are authorized to provide.

**Figure 2.**
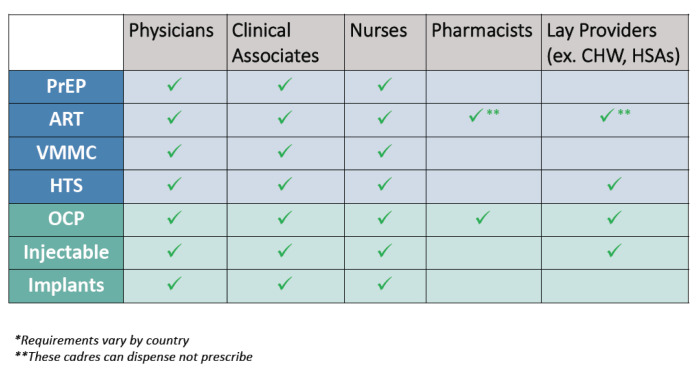
Task-shifting for administering contraceptives & HIV prevention interventions in SSA ^78^.

In Kenya and Zimbabwe, SRH healthcare providers cited a lack of training as the primary hindrance to providing HIV services, including screening and education about PrEP, in SRH settings. ^79, 80^ These providers expressed a lack of confidence to even bring up PrEP during FP counseling or if a high risk for HIV was detected. In Kenya, it was also reported that highly trained FP nurses in the public sector are often transferred to facilities in need of improving service delivery, ^81^ while in Malawi ^82^ and Zimbabwe, ^83^ severe health care worker shortages were underscored as barriers to integration. Training on the basics of oral PrEP and on screening high-risk individuals for eligibility would vastly improve the capacity of providers in SRH service areas to identify and refer potential clients for oral PrEP.

*“Sometimes a person can come to a facility where there is no one who can provide HIV testing, and end up receiving family planning services only because there was no one who could test her. Too few people are being trained, which leads to clients opting for other services because there is no one who can provide the service they want at the time of visit.”-Nurse, Zimbabwe* ^84^

### Limited engagement of the private sector

Leveraging the private sector ^85^ to enhance uptake is an important service delivery option that has the potential to increase access to HIV prevention, as it is already a well-established channel for SRH but has limited HIV prevention delivery across countries. Private sector services are an important source of health care in Kenya, where 34% of modern contraceptive methods are obtained via private providers, including 45% of oral contraceptive pills from pharmacies and 29% of injectables from private hospitals/clinics. ^86^ In Malawi and Zimbabwe, the private sector comprises about one-fifth of delivery channels for FP ^87, 88^ – an opportunity to integrate HIV prevention services – but access to private practitioners is limited to those who can afford it. NGOs, which are typically subsidized by donor funds, have been important private players in providing integrated SRH and HIV services for AGYW, but donor dependency may impede the sustainability of these channels. ^89^ The Malawian MOH has implemented public-private partnerships (PPPs) to ensure that clients are offered a full package of HIV/SRH services and fill gaps where public sector services are not attracting young people. Similarly, in Zimbabwe, the Ministry of Health and Child Care is implementing a PPP framework that aims to leverage resources from the public and private sectors to directly reduce the impact of HIV, AIDS and tuberculosis (TB) in the country. ^90^ Government-NGO collaboration in Malawi includes the work with Banja La Mtsogolo (BLM), which operates a “nested” approach in 14 sites, and public sector strengthening in 29 sites, to improve capacity for FP providers at public facilities, especially for LARCs. ^91^

Pharmacies are a critical channel for FP services in all three countries and an option with major potential for expanding HIV prevention commodities such as oral PrEP, once PrEP delivery is permitted. For example, 13.5% of contraceptives are accessed through pharmacies in Zimbabwe ^92^ and OCP and emergency contraception (EC) are available over-the-counter, but cost and fear of stigma are major challenges. PrEP is also available in pharmacies with a prescription, but stock-outs exist and it remains largely unaffordable. In Kenya, pharmacy-based OCP and PrEP-dispensing is allowed and pharmacies are already offering HIV testing. Kenyatta University, KEMRI and the University of Washington’s Pharmacy-based PrEP initiation implementation study is evaluating pharmacy prescription and refilling of PrEP through oversight of a remote physician and prescriptions offered based on rapid HIV testing. ^93^ Pharmacy provision of PrEP alongside FP could be an avenue for AGYW who can afford it, and may mitigate the stigma they often experience in facilities, although cost will be prohibitive for many AGYW.

## Service delivery

While integrated service delivery is not widespread across countries, due to a shared gap between policy and practice, Kenya, Malawi and Zimbabwe have employed different approaches to improving services for adolescents and pregnant women that offer lessons for scale up of HIV/SRH integration.

### Youth-friendly health services (YFHS)

Ensuring access to YFHS is a best practice for promoting uptake and effective use of HIV/SRH services among AGYW across SSA. Kenya, Malawi and Zimbabwe have policies that promote the expansion of YF service delivery, yet wide-scale implementation is hindered by limited resources in the public sector.

In Zimbabwe and Kenya, dialogues held with AGYW highlighted major breaches of confidence, discriminatory behavior, lack of respect for life choices and being turned away by healthcare providers when seeking SRH or HIV prevention services. ^94, 95^ In response, AGYW indicated they want guaranteed access to YFHS, defined as the full choice of products, access to free or affordable health services, to be treated with respect when accessing these services and provided with privacy and confidentiality throughout the process.

*“Service providers share your diagnosis in public and point to you where you are supposed to go… In most cases, people are divided according to what brought them there and this is not done in private e.g. ‘those with HIV come on this side!.’ [I]n order to avoid such humiliation, AGYW prefer to go to outside [of their locality].” -AGYW, Kenya* ^96^
*“Here (at a youth drop-in centre) I’m treated well. The staff is friendly and I meet other young people my age. I’m on PrEP and no one asks me why I need PrEP, like what have I done that warrants you taking PrEP.” -AGYW, Zimbabwe ^97^*

**Figure 3.**
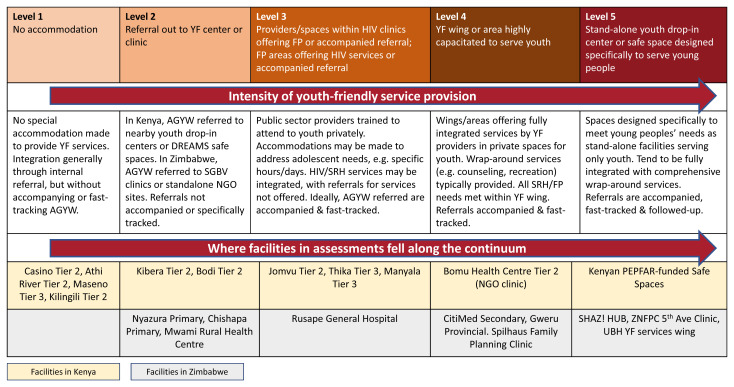
The integration-youth friendly (YF) continuum.

Health facility assessments in Kenya and Zimbabwe found that where available, YFHS provide the strongest examples of comprehensive, integrated HIV/SRH service delivery for adolescents and young people, and were found to mitigate stigma and increase access. Integration and YFHS were found to move together along a continuum, with more YF models exhibiting a greater level of HIV/SRH integration (see Figure 3). ^98, 99^ Low-volume facilities tend to be less YF and are integrated by default due to limited number of staff. High-volume sites tend to offer HIV and FP services in separate areas and rely on referrals to deliver comprehensive services. YF sites typically offer fully, intentionally integrated HIV/SRH and other services specifically for AGYW; these are often highly supported by implementing partners and donors, which equips them with additional resources and incentivizes meeting AGYW-focused targets. In the facility assessment in Kenya, facilities were generally less integrated and YF: four facilities fell in level 1, the least integrated/YF category; two facilities in level 2; three facilities in level 3; one facility in level 4 and PEPFAR-funded safe spaces represent level 5, the most integrated/YF level. In Zimbabwe, greater levels of integration and youth-friendliness were found, with no facilities found in level 1; three facilities in level 2; one facility in level 3; three facilities in level 4 and three facilities in level 5.

In Kenya, YFHS in the public sector are primarily available through stand-alone youth clinics and YF rooms or corners, where HIV testing, PrEP, FP and HIV care and treatment are available. ^100, 101^ YFHS are not widely embedded in public SRH units and primary health facilities and will require equipping facilities with YF corners and training healthcare providers. By contrast, Zimbabwe ^102– 104^ and Malawi ^105^ have moved toward integrating YFHS in public sector facilities, a model that is less expensive than stand-alone clinics and theoretically offers YFHS at all service delivery points, including FP, but availability of HIV prevention interventions, including oral PrEP, is limited. Further, access barriers remain: YFHS are sometimes only available during certain days or times, ^106, 107^ and providers reported that AGYW with children are often referred to general primary care, ^108^ a missed opportunity to provide them with the YFHS that they may prefer. Despite a recognition of the need for specific services for AGYW, the reach of YFHS remains very limited in Malawi. ^109^ Since the implementation of the YFHS program began in 2007, one comprehensive evaluation has been conducted to assess program coverage. The evaluation revealed that only half of CBDAs and 64% of peer educators had been trained in YFHS, including counseling on contraception and HIV/AIDS, and only 68% of health center providers had been trained to offer YFHS. The need for capacity-building for providers, as well as for youth and peer educators, is evident to mobilize AGYW to access SRH and HIV prevention services. ^110^

Across countries, YFHS supported by implementing partners are typically well-resourced “one-stop shops” or safe spaces for integrated HIV/SRH services, often with layered programming aimed at empowering AGYW (e.g., peer clubs and social asset-building). ^111– 114^ In our assessments, these sites showed the highest level of HIV/SRH integration and high utilization by AGYW. Most studies in the published literature focus on either SRH or HIV utilization and outcomes, rather than on integrated services or comprehensive HIV/SRH outcomes. ^115^ There is also a need to identify a core set of indicators to measure variables that may have a greater impact on the use of services by young people, such as confidentiality, privacy and accessibility of quality services. ^116^ Studies of YFHS for HIV have shown improvements among adolescents living with HIV around adherence and retention, ^117, 118^ as well as uptake of HIV testing and PrEP for those who qualify. ^119, 120^ Conducting more rigorous studies using a refined set of indicators that directly assess the benefit of delivering integrated services on both HIV and SRH outcomes, is critical to measure and compare the impact and effectiveness of YFHS, compared to the standard of care.

While many are donor-funded, understanding which aspects of YFHS are most impactful and valued by AGYW can inform approaches to integration in the public sector; ^121– 124^ operational research evaluating the effects of these models on uptake of both HIV prevention and SRH services is needed.

### Integrating PrEP delivery into PMTCT programs

Successful integration of PMTCT with MCH was supported by investments in human resources (e.g., training, task-shifting and hiring additional staff), largely from HIV donors. Similar investments are needed for integrating other HIV prevention and SRH services.

While primary HIV prevention among women of reproductive age and prevention of unintended pregnancies is an established prong of PMTCT programs, most countries have emphasized the prong related to preventing vertical transmission. ^125^ Kenya, Malawi and Zimbabwe have integrated PMTCT and SRH, where HIV testing and ART is routinely provided to pregnant and breastfeeding women (PBFW) upon HIV diagnosis during antenatal care (ANC) and post-partum visits. ^126^ However, there is a critical gap in primary prevention for PBFW who test negative for HIV during ANC, an important opportunity to expand integrated HIV/SRH services.

Malawi was the first country to pioneer Option B+ in late 2011, which offered all HIV-infected PBFW free, lifelong ART, regardless of CD4 count or clinical stage. Option B+ demonstrated the impact of integrated HIV services, especially for women living with HIV – an important advance recognized in the WHO global guidance. ^127^ Kenya and Zimbabwe adopted Option B+ in 2012 and 2013, respectively, and by 2019 all three countries achieved high HIV testing rates and with ART coverage exceeding 90 percent. ^128^ Despite significant progress, vertical transmission rates remain higher than expected, ^129^ due in part to new infections among women who had tested negative in early pregnancy, highlighting the need for early and repeated ANC visits, with repeated HIV testing throughout ANC, and ensuring PrEP provision for women who test negative. ^130^ Adolescent PBFW tend to have much lower rates of PMTCT and ANC uptake and face numerous challenges, including stigma from providers and communities and lack of social support. ^131– 133^

Although integrating ART and ANC services has contributed to reduced infections among children, preventing primary HIV infections among women has not been prioritized. In response, national strategic HIV plans in all three countries have underscored the need to address gaps in the unmet need for FP among women living with HIV, scale up PMTCT sites and services in general and ensure they are adolescent-friendly, strengthen community mobilization and community-based support systems for retention, and improve quality of care in integrated SRH, HIV, TB and RMNCAH services. ^134– 136^

By extension, oral PrEP rollout for PBFW has been slow due to limited safety data in this population until more recently and from lagging HIV prevention services for this population. But PrEP could become a basic intervention with greater confidence and evidence in PrEP use for this population. Oral PrEP is primarily offered in HIV clinics, contributing to low uptake and missing an opportunity to reach women via SRH channels, which tend to be preferred and accessed by more women. In Kenya, Comprehensive HIV Care Clinics (CCCs) and DREAMS safe spaces are the primary delivery channels of PrEP for AGYW. ^137^ With the exception of implementation research studies, ^138, 139^ PrEP is not offered in SRH services, and integration of PrEP is instead achieved through referral. Similarly, in Zimbabwe, while FP is well-integrated into HIV services, Opportunistic Infection (OI, or HIV treatment) clinics are the primary delivery channels for PrEP. SRH units rarely provide PrEP or offer referrals for PrEP services, except for pilot Zimbabwe National Family Planning Council (ZNFPC) sites ^140^ and YF facilities. ^141^ Until 2021, PrEP had been provided in Malawi in limited scope through NGOs, ^142^ but public sector clinics have begun to deliver PrEP to high-risk adolescents, and plan to scale up. There is a critical need to expand access to PrEP beyond HIV-focused clinics, as clients are being lost when referred, and to strengthen staff capacity and infrastructure to provide additional services.

Despite the remaining challenges, significant increases in ART coverage for women have been propelled by integrating ART into the points where women traditionally access health services. This was spurred by task-shifting, simplified delivery and linkages to community-based services and accelerated donor support – key elements needed to better integrate oral PrEP into broader SRH programs.

## Key gaps

### Targeting AGYW with demand generation

Demand generation activities for both HIV prevention and SRH to reach AGYW have not been scaled, despite supportive policies. ^143, 144^ Mobilizing demand for HIV prevention and SRH by providing information for AGYW, as well as for their partners, peers and communities, is necessary to increase access to services. ^145^ One implementing partner in Malawi emphasized, *“One of the most important things is that we need an avenue to reach girls, we need to have activities that matter to them (social assets, economic strengthening), where they can meet and we can bring services to them.”* ^146^ Sensitizing the broader community could de-stigmatize seeking services for AGYW, a prominent barrier.

*“Once the community spots you going behind the building [to the health facility], anyone can guess what you are there for.” -AGYW, Kenya* ^147^
*“The girls and women show real fear when you mention clinics and FP... You will even be forbidden from taking part in church activities just for going to a clinic.” -AGYW, Zimbabwe* ^148^
*“Outreach is more than giving out a flier. You need to go to high risk areas and have people who know how to have conversations with their peers to convince them to come in.” -Researcher, Malawi* ^149^

Demand generation strategies across countries rely on platforms and interlocutors whom AGYW trust, including youth or girls’ clubs, peer education or outreach activities (e.g., DREAMS ambassadors), as well as school-based activities. ^150^ These programs have demonstrated some successes in reaching AGYW in different age groups and different contexts with information about SRH and HIV prevention in accessible, non-stigmatizing ways, often through peer-led activities and community-based interventions. According to USAID, in 2020, DREAMS reached over 1.6 million AGYW with prevention services. ^151^ These strategies include education for 9-14 year-old boys and girls on primary prevention, complemented by outreach to their families and communities to bolster support for adolescents. DREAMS ambassadors, in particular, play a key role in linking AGYW to economic strengthening interventions, ^152^ as well as for gender-based violence (GBV), PrEP and other services. ^153^ The DREAMS program has seen an increase in PrEP uptake among AGYW in DREAMS districts. ^154^

Although strong programs exist to reach AGYW, there are generally low levels of information about HIV prevention in communities, and knowledge of HIV prevention hovers around 50% for AGYW across countries. ^155^ Coupled with myths and misconceptions on side effects and impact on fertility, this lack of and mis-information engenders fear and hinders social support for AGYW to use HIV prevention and SRH services. ^156^

Strategies to reduce unmet need or create demand tend to focus on specific FP or HIV prevention products (e.g., PrEP, long-acting contraception ^157^), rather than comprehensively considering integrated needs across all existing HIV prevention and FP options for AGYW. Demand generation is not typically well-funded nor integrated, so individual programs only provide information about certain services, reinforcing silos between HIV and SRH. ^158^ Designing demand generation initiatives aimed at building awareness of comprehensive HIV prevention and SRH will arm AGYW with holistic information to make decisions about their health and mitigate stigma in their networks. In Zimbabwe and Kenya, HIV communication strategies that promote integrated messages have been developed, but implementation remains under-resourced. ^159, 160^ Across countries, demand generation has historically been underfunded and will require dedicated investments from donors and governments to be effectively implemented.

### Structural environment

Integrating HIV prevention and SRH services is necessary but not sufficient to meet the needs of AGYW. Though largely outside the scope of these assessments, structural barriers – including gender inequalities and discriminatory cultural norms – that increase their risks of HIV, unintended pregnancy, STIs and early marriage, and prevent access to economic and educational opportunities, must simultaneously be addressed.

GBV increases HIV risk, and women and girls who experience violence are at greater risk for HIV. ^161^ Kenya, Malawi and Zimbabwe have recognized that addressing GBV is critical to their HIV responses. ^162^ Data from recent Violence Against Children Surveys (VACS) show high rates of violence across countries: in Kenya, 16% of girls experience sexual violence and 39% experience physical violence before age 18; ^163^ in Zimbabwe, 9% of girls experience sexual violence and 17% physical violence prior to age 18 ^164^ and in Malawi, one in five girls is sexually abused before age 18. ^165^ In Kenya and Zimbabwe, higher HIV prevalence rates were found among women who experienced childhood violence, underscoring the link between violence and HIV risk.

In Malawi and Zimbabwe, facilities that address GBV and offer integrated HIV/SRH services could be leveraged to minimize structural barriers. In Malawi, “Chikwanekwanes” (literally, “everything under one roof”) provide medical, legal and psychosocial services for survivors of sexual violence, including HIV testing, post-exposure prophylaxis (PEP) and follow-up testing, STI management and EC when indicated. ^166^ In Zimbabwe, Sexual Gender-Based Violence (SGBV) clinics, also known as “one-stop centres,” provide PEP, FP, counseling and YFHS in seven districts (in addition to psychosocial, legal and police support), though PrEP is not offered. In 2020, the United Nations Population Fund (UNFPA) allocated an additional $2.5 million to the GBV response in Zimbabwe, which included scaling up mobile one-stop centres to bring integrated, free services into communities, underscoring the value of delivering integrated health and social services to reach those most at-risk. ^167^

## Discussion

The changing funding, policy, research and development landscape highlights the growing urgency and commitment needed for HIV prevention and SRH integration. Both the United States President’s Emergency Plan for AIDS Relief (PEPFAR) and the Global Fund to Fight AIDS, TB, and Malaria (Global Fund), the largest funders of HIV programs globally, have recognized the importance of HIV/SRH integration for AGYW, and have increased their support for expanded access: PEPFAR allocated $17.5 million for oral PrEP in 2018 and $35.7 million in 2020, as well as a $1 billion investment in the DREAMS program, and the Global Fund allocated $140 million through the HER Initiative. ^168– 170^ Both initiatives show that layered social, structural and biomedical interventions – especially using YF approaches – can achieve impact for AGYW. ^171^ Current PEPFAR and Global Fund country plans prioritize bi-directional HIV/SRH integration by providing FP and STI testing within HIV programs, integrating oral PrEP across service delivery points, including ANC, ART, STI and FP services, and capacity-building for FP/MCH providers to supply oral PrEP. Making these changes now to expand access to oral PrEP also ensures greater access to HIV testing and other forms of HIV prevention (e.g., condoms, risk-reduction counseling) and will lay the foundation for expanding access to all biomedical prevention once new products enter the market.

A focus on delivering YFHS is critical to expanding access to integrated HIV/SRH services for AGYW. Data from DREAMS shows that new HIV infections among AGYW have been reduced in all the geographic settings where it operates: 96% of DREAMS districts saw a reduction of at least 25% and 62% saw a reduction greater than 40%. ^172^ In 2019, the Global Fund reached over 1 million young people between ages 10-24 with HIV prevention programs in Malawi, over 54,000 in Kenya and over 29,000 in Zimbabwe. ^173^ DREAMS has broad reach across countries, reaching 257,000 AGYW ages 10-24 in Kenya, with nearly 9,000 taking up oral PrEP; 185,403 AGYW in Zimbabwe, of which 2,000 initiated oral PrEP and nearly 60,000 AGYW in Malawi, ^174^ with oral PrEP rollout just beginning. All countries have plans for increasing the number of AGYW reached and for integrating oral PrEP into these programs in 2021.

Significant financial and institutional challenges, exacerbated by limited capacity and biases of health care providers toward sexually active AGYW, hamper HIV/SRH integration in public sector clinics. Externally-funded HIV programs have sometimes led to less integration, with targets for HIV to the exclusion of other SRH services. Across countries, provider capacity and YFHS tend to be highly dependent on implementing partner support, which equips them with additional resources and incentivizes achieving AGYW-focused targets. For instance, HIV funding from PEPFAR in 2020 was $33 million in Kenya, $177 million in Malawi, and $225 million in Zimbabwe, ^175^ and the Global Fund has allocated $272 million in Kenya, $393 million in Malawi and $425 million in Zimbabwe for HIV from 2020-2022. ^176^ Meanwhile, the combined total funding from UNFPA and the United States Agency for International Development (USAID) for contraceptive commodities alone across 69 countries was just $200 million in 2019. ^177^

More integrated donor-funded programs would bolster integration and YF efforts downstream at the point-of-care where it matters most. Unified donor targets could ensure that HIV prevention and SRH are given greater balance by programs and healthcare providers, and that providing YFHS in these settings is prioritized. Zeroing in on specific programmatic elements of DREAMS and Global Fund programs that can be adapted by the public sector could help scale up YFHS. ^178^ One USAID/Malawi official noted, *“The challenge is that DREAMS implementation can be expensive, the investment can’t be matched by the government, and we are not able to implement DREAMS everywhere. It is designed to be a time-limited high impact set of interventions to the most vulnerable girls to break the cycle of transmission and reduce the HIV incidence in high burden areas. Once the transmission cycle is broken then we will need to develop a ‘maintenance’ DREAMS-like programming that can be sustainably implemented by the host government.”* ^179^

There are also critical opportunities to expand access to integrated services through the private sector, particularly through pharmacies, which are a critical channel for FP services in all three countries and have major potential for expanding oral PrEP. In many resource-constrained settings, retail pharmacies fill an important gap in the health care system, providing access to treatment of urgent conditions, monitoring of chronic conditions, point-of-care testing and preventative care, ^180– 184^ and have been shown to increase access for young people. ^185^ Delivery of oral PrEP through pharmacies is utilized in the US, Europe and Asia, and studies have shown that oral PrEP can be successfully provided completely by pharmacists in these settings, with oversight by a remote physician. ^186^ A recent stakeholder consultation in Kenya showed that providers and implementers were strongly supportive of developing and testing a model for pharmacy-based oral PrEP delivery to increase oral PrEP access. ^187^ The consultative group developed a pathway for pilot testing pharmacy-based oral PrEP delivery in Kenya. Expanding PrEP delivery beyond health clinics and aligning FP and HIV prevention delivery channels has the potential to increase uptake of both PrEP and contraception, and respond to women’s desire for convenient and less stigmatized services.

A growing number of HIV prevention products – including a vaginal ring, injectable PrEP and multi-purpose prevention technologies – are pending regulatory approval and will be available in the next two to three years. Aligning the delivery channels where these products will be offered with contraception or as part of ANC visits would likely make access much easier for AGYW. Integrated services are more convenient, treat women holistically and reduce the burden placed on AGYW to access healthcare that meets their multiple and changing needs. Several studies have shown that when oral PrEP is co-delivered alongside FP, uptake of both increases. ^188, 189^ Accordingly, introducing new products for HIV prevention within integrated services could increase their uptake and continued use. FP healthcare providers are more familiar with administering a range of modalities, from pills to injections to vaginally inserted products, compared to HIV healthcare providers. FP healthcare providers also interact more frequently with women over a lifetime and, with adequate training and support, could be well-placed to address multiple health concerns. Advancing HIV prevention and SRH integration now could lay the groundwork for the faster and more equitable introduction of a range of technologies in the future. Putting AGYW at the center of care demands acting on commitments to HIV/SRH integration. Most importantly, AGYW are calling for services that are respectful, comprehensive, educational and empathetic, not patronizing or fragmented. ^190, 191^

Shared needs identified through rapid assessments in Kenya, Malawi and Zimbabwe were greater investments in provider capacity-building and demand generation to expand integrated service delivery, while country-specific needs for integration centered on health systems adaptations, donor support and scope of PrEP implementation. Further research is required to: 1) identify which interventions and delivery models are preferred by AGYW and correlated with better HIV and SRH outcomes; 2) determine the cost-effectiveness of integrating HIV prevention into SRH facility- and community-based delivery channels, expanding private sector access of HIV and SRH products through pharmacies and 3) pinpoint effective strategies for capacitating and supporting healthcare providers at the frontlines of delivering integrated care.

## Conclusions

While the call for HIV/SRH integration is not new, integration of HIV prevention and SRH has lagged, and integrated policies and programs preceding the introduction of oral PrEP have largely not been adapted. In Kenya, Malawi and Zimbabwe, YFHS and PMTCT programs offer the strongest examples of integration that can be leveraged and expanded to effectively reach AGYW. Provider capacity-building, synchronization of HIV and SRH services, demand generation and structural barriers warrant deeper attention if HIV prevention and SRH integration are to be realized at scale.

As new HIV prevention products are introduced, thus expanding the method mix and increasing awareness of HIV prevention more broadly, demand for integrated HIV/SRH services is likely to grow. Investing now in integrating HIV prevention and SRH across areas of health systems that will have synergistic effects, such as improving early and repeated ANC visits, streamlined SRH supply chains and expanding access to YFHS programs and pharmacy-based delivery, will alleviate pressure in the future; in addition, it will create a pathway for a more sustainable, government-led response amid a more complex prevention landscape and an inevitable decrease in external funding. Prioritizing integration can strengthen the response to the HIV epidemic while improving the health outcomes and lives of AGYW.

## Data availability

### Underlying data

Data underlying the results are available as part of the article and no additional source data are required. The assessments in Kenya, Malawi and Zimbabwe that are comparatively analyzed in this article are accessible via the following sources:

- *Integration of HIV prevention and sexual and reproductive health services in Kenya* is available at https://www.avac.org/resource/integration-hiv-prevention-and-sexual-reproductive-health-services-kenya.
- *Integration of HIV prevention and sexual and reproductive health services in Zimbabwe* is available at https://www.avac.org/resource/integration-hiv-prevention-and-srh-services-zimbabwe.
- *Opportunities and Challenges for the Integration of HIV and SRH Services in Malawi* is currently unpublished and available upon request under restricted access. To request access to this assessment, please email Sara Allinder at Sara.Allinder@georgetown.edu and Anna Carter at Anna.Carter@georgetown.edu.

## Footnotes

^1^ UNAIDS: AIDSinfo. 2020. Accessed May 14, 2021. Reference Source

^2^ AVAC: Understanding the Results of the ECHO Study. 2020. Reference Source

^3^ UNAIDS: AIDSinfo. 2020. Accessed May 14, 2021. Reference Source

^4^ UNAIDS: AIDSinfo. 2020. Accessed May 14, 2021. Reference Source

^5^ UNFPA: State of the World Population 2020. 2020. Reference Source

^6^ UNFPA: World Population Dashboard. 2021. Accessed May 14, 2021. Reference Source

^9^ FP2020: Zimbabwe: FP2020 Core Indicator Summary Sheet: 2019-2020 Annual Progress Report. 2020. Reference Source

^10^ AVAC: The Global PrEP Tracker. 2021. Accessed May 14, 2021. Reference Source

^11^ Kenya National Bureau of Statistics: Kenya Demographic and Health Survey 2014. 2015. Reference Source

^12^ Ministry of Health, National AIDS & STI Control Programme: Status on implementation of Oral Pre-exposure Prophylaxis for HIV in Kenya. Nairobi: NASCOP. 2018.

^13^ Zimbabwe National Statistics Agency and ICF International: Zimbabwe Demographic and Health Survey 2015: Final Report. Rockville, Maryland: Zimbabwe National Statistics Agency (ZIMSTAT) and ICF International. 2016. Reference Source

^14^ National Statistical Office (NSO) [Malawi] and ICF: Malawi Demographic and Health Survey 2015-16. Zomba, Malawi and Rockville, Maryland, USA: NSO and ICF. 2017. Reference Source

^15^ MacQuarrie, KLD: HIV/AIDS and Unmet Need for Family Planning. DHS Working Papers No. 122. Rockville, Maryland: ICF International. 2015. Reference Source

^16^ McCoy SI, Buzdugan R, Ralph L, *et al.*: Unmet need for family planning, contraceptive failure, and unintended pregnancy among HIV-infected and HIV-uninfected women in Zimbabwe. *PLoS One.* 2014; **9**(8): e105320. PubMed Abstract | Publisher Full Text

^17^ Rucinski KB, Powers KA, Schwartz SR, *et al.*: Longitudinal patterns of unmet need for contraception among women living with HIV on antiretroviral therapy in South Africa. *PLoS One.* 2018; **13**(12): e0209114. PubMed Abstract | Publisher Full Text

^18^ Abubeker FA, Fanta MB, Dalton VK: Unmet Need for Contraception among HIV-Positive Women Attending HIV Care and Treatment Service at Saint Paul’s Hospital Millennium Medical College, Addis Ababa, Ethiopia. *Int J Reprod Med.* 2019; **2019**: 3276780. PubMed Abstract | Publisher Full Text

^40^ HIV Prevention Market Manager, Ministry of Health [Kenya]: Integration of HIV prevention and sexual and reproductive health services in Kenya. 2020. Reference Source

^41^ HIV Prevention Market Manager, Ministry of Health and Child Care [Zimbabwe]: Integration of HIV prevention and sexual and reproductive health services in Zimbabwe. 2020. Reference Source

^42^ Georgetown University Center for Innovation in Global Health: Opportunities and Challenges for the Integration of HIV and SRH Services in Malawi. 2020. Unpublished report.

^43^ Interview with RH coordinator, Kenya, Nov. 2019.

^44^ Interview with MOH officials, Kenya, Nov. 2019.

^45^ On file with the national HIV/SRH integration sub-committee, Ministry of Health, Kenya.

^46^ HIV Prevention Market Manager, Ministry of Health and Child Care [Zimbabwe]: Integration of HIV prevention and sexual and reproductive health services in Zimbabwe. 2020. Reference Source

^47^ Interviews with key informants in Malawi, March-April 2020.

^48^ Interview with USAID/Malawi official, March 2020.

^49^ Interview with University of Malawi, College of Medicine, professor, April 2020.

^50^ Interview with County AIDS and STI Coordinator (CASCO), Kenya, Nov. 2019.

^51^ Interview with MoHCC official, Zimbabwe, March 2020.

^52^ Interview with YF provider, Zimbabwe, March 2020.

^53^ Smit JA, Church K, Milford C, *et al.*: Key informant perspectives on policy- and service-level challenges and opportunities for delivering integrated sexual and reproductive health and HIV care in South Africa. *BMC Health Serv Res.* 2012; **12**: 48. PubMed Abstract | Publisher Full Text

^56^ Kangudie DM, Guidigbi H, Mensah S, *et al*.: Effective integration of sexual reproductive health and HIV prevention, treatment, and care services across sub-Saharan Africa: where is the evidence for program implementation? *Reprod Health*. 2019; **16**(Suppl 1): 56. PubMed Abstract | Publisher Full Text

^57^ Ministry of Health [Kenya]: National Family Planning Costed Implementation Plan 2017-2020. Nairobi: Ministry of Health. 2017. Reference Source

^58^ Interview with registered MCH nurse, Kenya, Nov. 2019.

^59^ Interview with MoHCC official, Zimbabwe, May 2020.

^60^ Dehnavieh R, Haghdoost A, Khosravi A, *et al.*: The District Health Information System (DHIS2): A literature review and meta-synthesis of its strengths and operational challenges based on the experiences of 11 countries. *Health Inf Manag.* 2019; **48**(2): 62-75. PubMed Abstract | Publisher Full Text

^61^ Interview with healthcare providers, Zimbabwe, March 2020.

^62^ Interview with implementing partner, Kenya, Nov. 2019.

^63^ Hagel C, Paton C, Mbevi G, *et al.*: Data for tracking SDGs: challenges in capturing neonatal data from hospitals in Kenya. *BMJ Glob Health.* 2020; **5**(3): e002108. PubMed Abstract | Publisher Full Text

^64^ Njeru I, Kareko D, Kisangau N, *et al.*: Use of technology for public health surveillance reporting: opportunities, challenges and lessons learnt from Kenya. *BMC Public Health.* 2020; **20**(1): 1101. PubMed Abstract | Publisher Full Text

^65^ Interview with implementing partner, Malawi, April 2020.

^66^ Interview, Malawi, March 2020.

^67^ Inter-Agency Task Team for the Prevention and Treatment of HIV Infection in Pregnant Women, Mothers and Children: HIV and Sexual and Reproductive Health Programming: Innovative Approaches to Integrated Service Delivery. 2014. Reference Source

^68^ Interview with healthcare providers, Kenya, Nov. 2019.

^70^ Interview with healthcare providers, Zimbabwe, March 2020.

^71^ Fitch L, Rodrigues J, Warren M: How policy affects practice: policy barriers to provision of HIV biomedical prevention services in Sub-Saharan Africa. 2020. Reference Source

^72^ Seidman G, Atun R: Does task shifting yield cost savings and improve efficiency for health systems? A systematic review of evidence from low-income and middle-income countries. *Hum Resour Health.* 2017; **15**: 29. PubMed Abstract | Publisher Full Text

^73^ Olawo AA, Bashir I, Solomon M, *et al.*: “A cup of tea with our CBD agent … ”: community provision of injectable contraceptives in Kenya is safe and feasible. *Glob Health Sci Pract.* 2013; **1**(3): 308–315. PubMed Abstract | Publisher Full Text

^74^ Extending Service Delivery (ESD) Project, USAID: Extending Service Delivery Project: Best Practice Brief # 1: Integrating HIV Services in Local Family Planning: The Expanded Community-Based Distribution Model and Zimbabwe Experience. Washington, DC: ESD Project, USAID. Reference Source

^75^ Rapiti R, Mullick S: An evaluation of Population Services Zimbabwe’s mobile outreach model. Nairobi: Population Council. 2014. Reference Source

^76^ Interview, Malawi, April 2020.

^77^ Republic of Malawi, Ministry of Health: National Sexual and Reproductive Health and Rights (SRHR) Policy. Lilongwe: Ministry of Health. 2016.

^78^ Fitch L, Rodrigues J, Warren M: How policy affects practice: policy barriers to provision of HIV biomedical prevention services in Sub-Saharan Africa. 2020. Reference Source

^79^ Interview with healthcare providers, Kenya, Nov. 2019.

^80^ Interview with healthcare providers, Zimbabwe, March 2020.

^81^ Interview with implementing partners, Kenya, Nov. 2019.

^82^ Republic of Malawi, Ministry of Health: National Sexual and Reproductive Health and Rights (SRHR) Policy. Lilongwe: Ministry of Health. 2016.

^83^ HIV Prevention Market Manager, Ministry of Health and Child Care [Zimbabwe]: Integration of HIV prevention and sexual and reproductive health services in Zimbabwe. 2020. Reference Source

^84^ Interview with registered nurse, Zimbabwe, March 2020.

^85^ The WHO defines the private sector as “those individuals and organizations providing health services or products that are not owned or directly controlled by government.” These include pharmacies, private practitioners and NGOs. WHO: Technical Series on Primary Care: The private sector, universal health coverage and primary health care. Geneva: WHO. 2018. Reference Source

^86^ Kenya National Bureau of Statistics: Kenya Demographic and Health Survey 2014. 2015. Reference Source

^87^ National Statistical Office (NSO) [Malawi] and ICF: Malawi Demographic and Health Survey 2015-16. Zomba, Malawi and Rockville, Maryland, USA: NSO and ICF. 2017. Reference Source

^88^ Zimbabwe National Statistics Agency and ICF International: Zimbabwe Demographic and Health Survey 2015: Final Report. Rockville, Maryland: Zimbabwe National Statistics Agency (ZIMSTAT) and ICF International. 2016. Reference Source

^89^ In Malawi, e.g., the Christian Health Association of Malawi (CHAM), Banja La Mtsogolo (BLM) (MSI affiliate), Family Planning Association of Malawi (FPAM) (IPPF affiliate) and Population Services International (PSI). In Zimbabwe, e.g., Population Services Zimbabwe (MSI affiliate), Pangaea Zimbabwe AIDS Trust (PZAT) and PSI.

^90^ Government of Zimbabwe, Ministry of Health and Child Care: Strategic Framework for Public-Private Partnerships for TB and HIV Prevention, Treatment, Care and Support.

^91^ Interview with implementing partner, Malawi, April 2020.

^92^ Zimbabwe National Statistics Agency and ICF International: Zimbabwe Demographic and Health Survey 2015: Final Report. Rockville, Maryland: Zimbabwe National Statistics Agency (ZIMSTAT) and ICF International. 2016. Reference Source

^93^ University of Washington. Pharmacy Delivery to Expand the Reach of PrEP in Kenya. In: ClinicalTrials.gov [cited 2021 May 25], Available from https://clinicaltrials.gov/ct2/show/NCT04558554.

^94^ Civil society dialogues with AGYW and civil society organizations, Zimbabwe, Nov. 2019.

^95^ Civil society dialogues with AGYW, Kenya, Nov. 2019.

^96^ Civil society dialogues with AGYW, Kenya, Nov. 2019.

^97^ Civil society dialogues with AGYW, Zimbabwe, Nov. 2019.

^98^ Facility assessment data analysis, Kenya, Nov. 2019.

^99^ Facility assessment data analysis, Zimbabwe, March-June 2020.

^100^ Facility assessment data analysis, Kenya, Nov. 2019.

^101^ Interview with healthcare providers and implementing partners, Kenya, Nov. 2019.

^102^ Facility assessment data analysis, Zimbabwe, March-June 2020.

^104^ Government of Zimbabwe, Ministry of Health and Child Care: National Adolescent and Youth Sexual and Reproductive Health (ASRH) Strategy II: 2016-2020. Harare: Ministry of Health and Child Care. 2016.

^105^ Republic of Malawi, Ministry of Health: National Youth Friendly Health Services Strategy, 2015-2020. Lilongwe: Ministry of Health. 2015.

^106^ Facility assessment data analysis, Zimbabwe, March-June 2020.

^107^ Facility assessment data analysis, Kenya, Nov. 2019.

^108^ Interview with healthcare providers, Zimbabwe, March 2020.

^109^ Government of Malawi: National Strategy for Adolescent Girls and Young Women 2018-2022. Lilongwe: Government of Malawi. 2018.

^110^ Ministry of Health [Malawi], Reproductive Health Department, USAID, Evidence to Action Project, Centre for Social Research, University of Malawi: Evaluation of Youth-Friendly Health Services in Malawi. 2014. Reference Source

^111^ Facility assessment data analysis, Zimbabwe, March-June 2020.

^114^ PEPFAR: Zimbabwe Country Operational Plan (COP) 2020: Strategic Direction Summary. 2020. Reference Source

^115^ Brittain AW, Loyola Briceno AC, Pazol K, *et al.*: Youth-Friendly Family Planning Services for Young People: A Systematic Review Update. *Am J Prev Med.* 2018; **55**(5): 725-735. PubMed Abstract | Publisher Full Text

^116^ Mazur A, Brindis CD, Decker MJ: Assessing youth-friendly sexual and reproductive health services: a systematic review. *BMC Health Serv Res.* 2018; **18**(1): 216. PubMed Abstract | Publisher Full Text

^117^ Lee L, Yehia BR, Gaur AH, *et al*.: The Impact of Youth-Friendly Structures of Care on Retention Among HIV-Infected Youth. *AIDS Patient Care STDS.* 2016; **30**(4): 170-7. PubMed Abstract | Publisher Full Text

^118^ Rosenberg NE, Bhushan NL, Vansia D, *et al.*: Comparing Youth-Friendly Health Services to the Standard of Care Through “Girl Power-Malawi”: A Quasi-Experimental Cohort Study. *J Acquir Immune Defic Syndr.* 2018; **79**(4): 458-466. PubMed Abstract | Publisher Full Text

^119^ Kidman R, Waidler J, Palermo T: Uptake of HIV testing among adolescents and associated adolescent-friendly services. *BMC Health Serv Res.* 2020; **20**(1): 881. PubMed Abstract | Publisher Full Text

^120^ Velloza J, Kapogiannis B, Bekker LG, *et al*.: Interventions to improve daily medication use among adolescents and young adults: what can we learn for youth pre-exposure prophylaxis services? *AIDS.* 2021; **35**(3): 463-475. PubMed Abstract | Publisher Full Text

^121^ Civil society dialogues with AGYW, Kenya, Nov. 2019.

^124^ Denno DM, Hoopes AJ, Chandra-Mouli V: Effective Strategies to Provide Adolescent Sexual and Reproductive Health Services and to Increase Demand and Community Support. *J Adolesc Health.* 2015; **56**(1): S22-S41. PubMed Abstract | Publisher Full Text

^125^ Callahan T, Modi S, Swanson J, *et al*.: Pregnant adolescents living with HIV: what we know, what we need to know, where we need to go. *J Int AIDS Soc.* 2017; **20**(1): 21858. PubMed Abstract | Publisher Full Text

^126^ UNAIDS: Progress towards the Start Free, Stay Free, AIDS Free targets: 2020 report. Geneva: UNAIDS. 2020. Reference Source

^127^ WHO: Consolidated Guidelines on the Use of Antiretroviral Drugs for Treating and Preventing HIV Infection: Recommendations for a Public Health Approach. WHO: Geneva. 2016. Reference Source

^128^ UNAIDS: AIDSinfo. 2020. Accessed May 14, 2021. Reference Source

^129^ UNAIDS: AIDSinfo. 2020. Accessed May 14, 2021. Reference Source

^130^ PEPFAR: Malawi Country Operational Plan 2020: Strategic Direction Summary. 2020. Reference Source

^131^ Callahan T, Modi S, Swanson J, *et al*.: Pregnant adolescents living with HIV: what we know, what we need to know, where we need to go. *J Int AIDS Soc.* 2017; **20**(1): 21858. PubMed Abstract | Publisher Full Text

^133^ Carbone NB, Njala J, Jackson DJ, *et al.*: “I would love if there was a young woman to encourage us, to ease our anxiety which we would have if we were alone”: Adapting the Mothers2Mothers Mentor Mother Model for adolescent mothers living with HIV in Malawi. *PLoS One.* 2019; **14**(6): e0217693. PubMed Abstract | Publisher Full Text

^134^ Ministry of Health, NASCOP: Kenya Framework for Elimination of Mother-To-Child Transmission of HIV and Syphilis 2016-2021. Nairobi: NASCOP. 2016.

^136^ Government of Zimbabwe, Ministry of Health and Child Care: Plan for Elimination of Mother to Child Transmission of HIV & Syphilis in Zimbabwe 2018-2022. Harare: Ministry of Health and Child Care. 2018. Reference Source

^137^ Facility assessment data analysis, Kenya, Nov. 2019.

^138^ Mugwanya KK, Pintye J, Kinuthia J, *et al.*: Integrating preexposure prophylaxis delivery in routine family planning clinics: A feasibility programmatic evaluation in Kenya. *PLOS Medicine.* 2019; **16**(9): e1002885. PubMed Abstract | Publisher Full Text

^139^ Kinuthia J, Pintye J, Abuna F, *et al.*: Pre-exposure prophylaxis uptake and early continuation among pregnant and post-partum women within maternal and child health clinics in Kenya: results from an implementation programme. *Lancet HIV.* 2020; **7**(1): E38-E48. PubMed Abstract | Publisher Full Text

^140^ Gombe MM, Cakouros BE, Ncube G, *et al.*: Key barriers and enablers associated with uptake and continuation of oral pre-exposure prophylaxis (PrEP) in the public sector in Zimbabwe: Qualitative perspectives of general population clients at high risk for HIV. *PLoS One.* 2020; **15**(1): e0227632. PubMed Abstract | Publisher Full Text

^141^ Interview with MOH officials, healthcare providers and implementing partners, Zimbabwe, March-June 2020.

^142^ Interview with implementing partners, Malawi, April 2020.

^143^ Government of Zimbabwe, Ministry of Health and Child Care: Comprehensive National HIV Communications Strategy for Zimbabwe: 2019-2025. Harare: Ministry of Health and Child Care. 2019. Reference Source

^144^ Ministry of Health, NASCOP: National HIV & STI Combination Prevention Communication Strategy, 2019-2024. 2019.

^145^ Interview with implementing partners, Malawi, March-April 2020, and MOH officials, Malawi, July 2020.

^146^ Interview with implementing partner, March 2020.

^147^ Civil society dialogues with AGYW, Kenya, Nov. 2019.

^148^ Civil society dialogues with AGYW and civil society organizations, Zimbabwe, Nov. 2019.

^149^ Interview with local researcher, Malawi, April 2020.

^150^ Interview with implementing partners, Malawi, March-April 2020.

^151^ USAID: DREAMS: Partnership to Reduce HIV/AIDS in Adolescent Girls and Young Women. 2021. Accessed May 25, 2021. Reference Source

^152^ PEPFAR: Country Operational Plan (COP/ROP) 2020 [Kenya]: Strategic Direction Summary. 2020. Reference Source

^153^ PEPFAR: Malawi Country Operational Plan 2020: Strategic Direction Summary. 2020. Reference Source

^154^ Global PrEP Learning Network: PrEP for Adolescent Girls and Young Women (AGYW): Experiences From DREAMS Programs. 2020. Reference Source

^155^ UNAIDS: AIDSinfo. 2020. Accessed May 14, 2021. Reference Source

^156^ HIV Prevention Market Manager: What we know and don’t know about adolescent girls and young women and HIV prevention in sub-Saharan Africa: Mapping findings across completed, ongoing and planned projects. 2018. Reference Source

^157^ Rademacher KH, Solomon M, Brett T, *et al*.: Expanding Access to a New, More Affordable Levonorgestrel Intrauterine System in Kenya: Service Delivery Costs Compared With Other Contraceptive Methods and Perspectives of Key Opinion Leaders. *Glob Health Sci Pract.* 2016; **4**(S2): S83-93. PubMed Abstract | Publisher Full Text

^158^ OPTIONS Consortium: Global PrEP Learning Network: PrEP Integration: Including PrEP with Family Planning Services. 2019. Reference Source

^159^ Government of Zimbabwe, Ministry of Health and Child Care: Comprehensive National HIV Communications Strategy for Zimbabwe: 2019-2025. Harare: Ministry of Health and Child Care. 2019. Reference Source

^160^ Ministry of Health, NASCOP: National HIV & STI Combination Prevention Communication Strategy, 2019-2024. 2019.

^161^ UNAIDS: Unite with Women, Unite against Violence and HIV. Geneva: UNAIDS. 2014. Reference Source

^162^ Ministry of Labour and Social Protection, State Department for Social Protection, Department of Children’s Services: National Prevention and Response Plan on Violence against Children in Kenya 2019-2023. Nairobi: Government of Kenya. 2020. Reference Source

^163^ Together for Girls, Ministry of Labour and Social Protection [Kenya]: Violence Against Children Survey Report 2019 fact sheet. 2020. Reference Source

^164^ Together for Girls, Government of Zimbabwe: Violence Against Children Survey Report 2019 fact sheet. 2019. Reference Source

^165^ Ministry of Gender, Children, Disability and Social Welfare, Republic of Malawi: Executive Summary: Violence Against Children and Young Women in Malawi: Findings from a National Survey 2013. 2014. Reference Source

^166^ Mulambia Y, Miller AJ, MacDonald G, *et al*.: Are one-stop centres an appropriate model to deliver services to sexually abused children in urban Malawi?, *BMC Pediatr.* 2018; **18**: 145. PubMed Abstract | Publisher Full Text

^167^ UNFPA Zimbabwe: UNFPA releases over $2,5 million to support access to scale up provision of Gender Based Violence & sexual reproductive health services during the COVID19 pandemic. 2020. Accessed May 25, 2021. Reference Source

^168^ PEPFAR: PEPFAR 2021 Annual Report to Congress. Washington, DC: PEPFAR. 2021. Reference Source

^170^ The Global Fund: Women & Girls. Accessed May 25, 2021. Reference Source

^171^ U.S. Department of State: DREAMS Partnership. Accessed May 25, 2021. Reference Source

^172^ PEPFAR: PEPFAR 2021 Annual Report to Congress. Washington, DC: PEPFAR. 2021. Reference Source

^173^ The Global Fund: The Global Fund Data Explorer. Accessed May 25, 2021. Reference Source

^174^ PEPFAR: Malawi: DREAMS Overview (FY 2016-2021). 2020. Reference Source

^175^ amfAR: PEPFAR Country/Regional Operating Plans (COPs/ROPs) Database. Accessed May 25, 2021. Reference Source

^176^ The Global Fund: The Global Fund Data Explorer. Accessed May 25, 2021. Reference Source

^177^ CHAI, Reproductive Health Supplies Coalition: Family Planning Market Report. 2020. Reference Source

^178^ Georgetown University Center for Innovation in Global Health: Opportunities and Challenges for the Integration of HIV and SRH Services in Malawi. 2020. Unpublished report.

^179^ Email correspondence with USAID/Malawi official, Aug. 2020.

^180^ Bigogo G, Audi A, Aura B, *et al.*: Health-seeking patterns among participants of population-based morbidity surveillance in rural western Kenya: implications for calculating disease rates. *Int J Infect Dis.* 2010; **14**(11): e967–e973. PubMed Abstract | Publisher Full Text

^184^ Ladner J, Davis B, Audureau E, *et al*.: Treatment-seeking patterns for malaria in pharmacies in five sub-Saharan African countries. *Malar J.* 2017; **16**(1): 353. PubMed Abstract | Publisher Full Text

^185^ Mugo PM, Prins HAB, Wahome EW, *et al*.: Engaging young adult clients of community pharmacies for HIV screening in coastal Kenya: a cross-sectional study. *Sex Transm Infect.* 2015; **91**(4): 257–259. PubMed Abstract | Publisher Full Text

^186^ Tung EL, Thomas A, Eichner A, *et al*.: Implementation of a community pharmacy-based pre-exposure prophylaxis service: a novel model for pre-exposure prophylaxis care. *Sex Health.* 2018; **15**(6): 556–561. PubMed Abstract | Publisher Full Text

^187^ Ortblad KF, Mogere P, Roche S, *et al.*: Design of a care pathway for pharmacy-based PrEP delivery in Kenya: results from a collaborative stakeholder consultation. *BMC Health Serv Res*. 2020; **20**(1): 1034. PubMed Abstract | Publisher Full Text

^188^ Mugwanya KK, Pintye J, Kinuthia J, *et al.*: Integrating preexposure prophylaxis delivery in routine family planning clinics: A feasibility programmatic evaluation in Kenya. *PLOS Medicine.* 2019; **16**(9): e1002885. PubMed Abstract | Publisher Full Text

^189^ Rousseau E, Baeten JM, Morton J, *et al.*: Association between contraceptive use and PrEP uptake and continuation in adolescent girls and young women in Cape Town, South Africa. 2019. Reference Source

^190^ Civil society dialogues with AGYW, Kenya, Nov. 2019.

^191^ Civil society dialogues with AGYW and civil society organizations, Zimbabwe, Nov. 2019.

