# Integration of HIV prevention and sexual and reproductive health in the era of anti-retroviral-based prevention: findings from assessments in Kenya, Malawi and Zimbabwe

**DOI:** 10.12688/gatesopenres.13330.2

**Published:** 2022-03-28

**Authors:** Fannie Kachale, Imelda Mahaka, Fatima Mhuriro, Mary Mugambi, Joseph Murungu, Barbra Ncube, Getrude Ncube, Albert Ndwiga, Rose Nyirenda, Violet Otindo, Anna Carter, Megan Dunbar, Janet Fleischman, Jessica Rodrigues, Kate Segal

**Affiliations:** 1 Department of Reproductive Health, Ministry of Health, Lilongwe, Malawi; 2 Pangaea Zimbabwe AIDS Trust (PZAT), Harare, Zimbabwe; 3 Reproductive Health Unit, Ministry of Health and Child Care, Harare, Zimbabwe; 4 National AIDS & STI Control Programme (NASCOP), Ministry of Health, Nairobi, Kenya; 5 Department of AIDS & TB, Ministry of Health and Child Care, Harare, Zimbabwe; 6 Department of Family Health, Ministry of Health, Nairobi, Kenya; 7 Department of HIV/AIDS, Ministry of Health, Lilongwe, Malawi; 8 Georgetown University Center for Innovation in Global Health, Washington, DC, USA; 9 Consultant, AVAC, New York, USA; 10 Consultant, Georgetown University Center for Innovation in Global Health, Washington, DC, USA; 11 AVAC, New York, USA

**Keywords:** HIV prevention, sexual and reproductive health, integration, oral pre-exposure prophylaxis, family planning, adolescent girls and young women

## Abstract

**Background: **Though substantial progress has helped curb the HIV epidemic, high rates of new HIV infections persist among adolescent girls and young women (AGYW) in sub-Saharan Africa, reflecting critical gaps in reaching them with integrated HIV prevention and sexual and reproductive health (SRH) services. The scale-up of oral pre-exposure prophylaxis (PrEP) and multiple novel HIV prevention products on the horizon offer countries a unique opportunity to expand innovative approaches to deliver comprehensive, integrated HIV prevention/SRH services.

**Methods: **This article comparatively analyzes findings from rapid assessments in Kenya, Malawi and Zimbabwe across key themes to highlight cross-country trends and contextual realities around HIV prevention/SRH integration, with a focus on oral PrEP and contraception. In Kenya and Zimbabwe, assessments were completed by Ministries of Health (MOH) and the HIV Prevention Market Manager and include 20 health facility assessments, 73 key informant interviews (KIIs) and six community dialogues. In Malawi, the assessment was completed by the MOH and Georgetown University Center for Innovation in Global Health and includes 70 KIIs and a review of national policies and program implementation in Blantyre. Findings were contextualized through a review of literature and policies in each country.

**Results: **Across countries, the policy environment is conducive to HIV prevention/SRH integration, though operationalization presents ongoing challenges, with most policies preceding and not accounting for oral PrEP rollout. National coordination mechanisms, youth-friendly health services and prevention of mother-to-child transmission programs are promising practices, while siloed and resource-constrained health systems, limited provider capacity, underfunded demand generation and structural factors exacerbate barriers to achieving integration.

**Conclusions: **As new HIV prevention products are introduced, demand for integrated HIV prevention/SRH services is likely to grow. Investing in HIV prevention/SRH integration can help to ensure a sustainable response to the HIV epidemic, streamline service delivery and improve the health outcomes and lives of AGYW.

## Introduction

Though substantial progress has been made to curb the HIV epidemic over the past decade, high rates of new HIV infections persist, especially among adolescent girls and young women (AGYW) in sub-Saharan Africa (SSA), who in 2019 had approximately three times the HIV incidence rate of their male counterparts. ^1^ To meet global HIV prevention goals and improve health outcomes, prevention programs need to reach AGYW. The 2019 results of the Evidence for Contraceptive Options in HIV Outcomes study (ECHO) underscored a critical gap in targeting AGYW with integrated HIV prevention and sexual and reproductive health (SRH) services. ^2^ The SARS-CoV-2 (COVID-19) pandemic has amplified the urgency for integration, as dire social and economic impacts heightened AGYW risk of HIV and STI infection and unintended pregnancy, disrupted critical HIV and SRH services and further constrained health systems and healthcare workers in many low- and middle-income settings. With the scale up of oral pre-exposure prophylaxis (PrEP) and introduction of multiple novel HIV prevention products on the horizon, health systems are at a turning point, with an opportunity to expand new and innovative approaches to reach AGYW with comprehensive HIV prevention/SRH services, including expanding access to existing and future PrEP products.

This article analyzes the progress that Kenya, Malawi and Zimbabwe have made towards the integration of HIV prevention and SRH (HIV prevention/SRH integration). While the term HIV prevention/SRH integration includes an array of services, this article focuses primarily on the integration of oral PrEP and family planning (FP). Examples from each country highlight promising approaches to integration in the context of HIV prevention, identifying opportunities for improved service delivery and pinpointing persistent gaps that effectively limit the impact of high-potential interventions. By examining cross-country experiences, this article aims to highlight regional trends and context-specific realities around advancing HIV prevention/SRH integration. Lessons can inform the scale-up of HIV prevention/SRH integration in other high HIV-burden countries, though increased government and donor investment and political support will be required.

### HIV prevention and SRH trends and landscape

**Figure 1.**
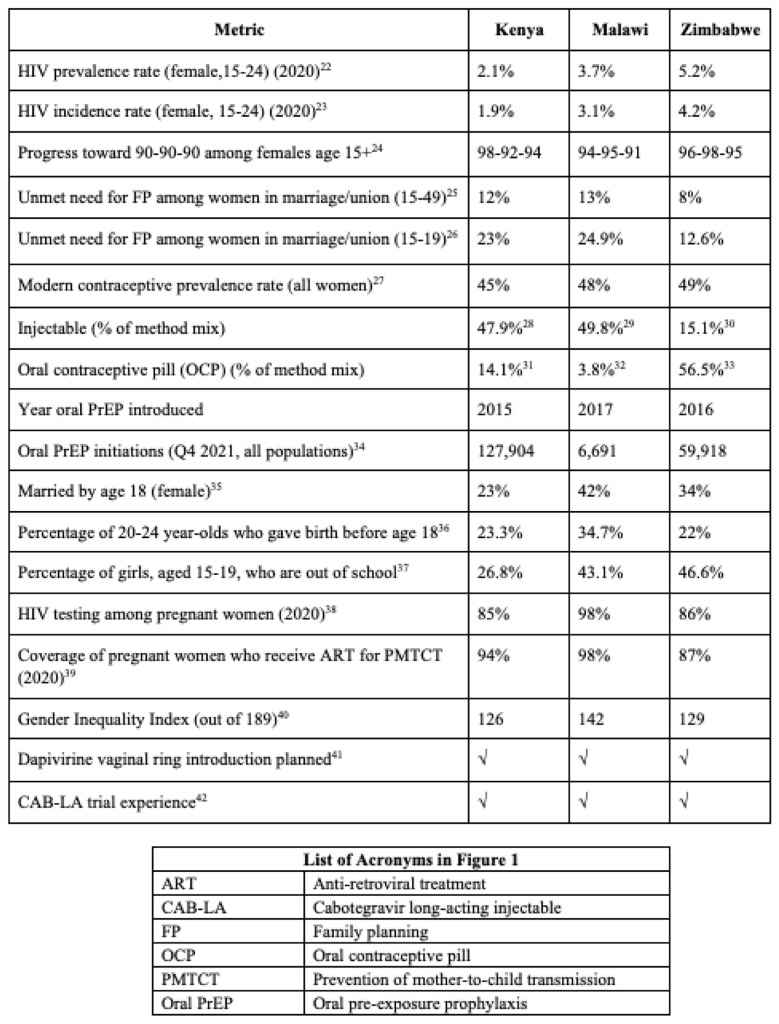
Comparison of demographic and epidemiological data in Kenya, Malawi and Zimbabwe.

Across countries, data reveal high HIV prevalence among young women, with especially high rates in Zimbabwe and Malawi (see Figure 1). ^3^ Data show significant strides in HIV testing among pregnant women, prevention of mother-to-child transmission (PMTCT) coverage and relatively high modern contraceptive prevalence rates for all women, pointing to SRH and maternal child health (MCH) services as strong entry points for integrated services. ^4, 5^ Unmet need for FP is higher in Kenya and Malawi than in Zimbabwe, and the contraceptive method mix in Kenya and Malawi skews toward injectables, while in Zimbabwe, the oral contraceptive pill is the most common method. ^6– 9^ Kenya was the first of these countries to introduce oral PrEP and is furthest ahead with scale-up, while Malawi’s program is just beginning to roll out. ^10^

Across countries, contraception and oral PrEP are primarily accessed in public facilities, though the private sector, including healthcare providers in private practice, non-governmental organizations (NGOs), faith-based clinics and pharmacies, is an important source of FP services, pointing to opportunities for integration with oral PrEP. In Kenya, 60% of contraceptives are obtained in the public sector and 34% in the private sector, while oral PrEP is delivered primarily in HIV clinics (57%) and safe spaces (25%, many NGO-run). ^11, 12^ Similarly, in Zimbabwe, 73% of contraceptives are obtained in the public sector and 22% via the private sector; oral PrEP is primarily obtained in public HIV clinics. ^13^ In Malawi, 79% of contraceptives are accessed in public facilities, 8% from Banja la Mtsogolo (BLM), the local Marie Stopes affiliate, and 6% from the private sector. ^14^ Notably, the COVID-19 pandemic has expanded innovations in service delivery, broadening access to oral PrEP and FP through decentralized, differentiated and private sector channels in an effort to mitigate disruptions to accessing services at public health facilities.

High HIV prevalence and incidence among AGYW in Kenya (2.1% and 1.9%), Malawi (3.7% and 3.1%) and Zimbabwe (5.2% and 4.2%), ^15, 16^ and the large percentage of AGYW in these countries who gave birth before age 18 (23.3%, 34.7% and 22%, respectively), ^17^ signal the need to better reach this population with HIV prevention and SRH information and services. High rates of early marriage in Malawi and low rates of secondary school enrolment for girls in Malawi and Zimbabwe underscore that structural and social barriers often undermine health outcomes. Limited data on AGYW points to a need for sex- and age-disaggregated data to better tailor programs. While the unmet need for FP among women of reproductive age ranges from 10% in Zimbabwe to 15% in Malawi, this figure is higher among AGYW (ranging from 12.6% in Zimbabwe to 24.9% in Malawi). Unmet need for FP among women living with HIV has been shown to be slightly lower than among women in the general population, ^18, 19^ though studies cite missed opportunities to provide FP through anti-retroviral treatment (ART) clinics as a contributing factor to the ongoing unmet need within this population. ^20, 21^

## Methods

This article is a comparative analysis of findings from three completed rapid health systems and landscaping assessments of the integration of HIV prevention, including oral PrEP, in SRH services in Kenya, Malawi and Zimbabwe. ^43– 45^ These rapid assessments utilized proven methodologies for rapid assessments, building from WHO and Measure Evaluation’s “Routine Health Information System Rapid Assessment Tool.” ^46^ The assessments collected programmatic and clinic data from public health facilities and NGO-supported programs, and gathered insights from Ministry of Health (MOH) officials, providers, NGO staff and others working at district/county levels, and employed a thematic analysis of data generated, with the intent to rapidly inform national policies and programs. By synthesizing, analyzing and contextualizing the findings of the three assessments, this article aims to understand the current state of HIV prevention/SRH integration in each country and to elucidate cross-country trends. Summaries of and quotes from primary qualitative data from the rapid assessments were included in this article to underscore findings, and outside literature was incorporated to provide additional context. Because the rapid assessments comprise the main source material of this article, their methodologies are elaborated upon below.

### Research teams for rapid assessments

In Kenya ^47^ and Zimbabwe, ^48^ the rapid assessments were completed in 2020 by multi-disciplinary teams from the MOH and the HIV Prevention Market Manager project (PMM), represented by AVAC. In Kenya, the research team included representatives from the National AIDS and STI Control Programme (NASCOP), Department of Family Health and AVAC. In Zimbabwe, the research team included representatives from the Department of AIDS & TB, Pangaea Zimbabwe AIDS Trust (PZAT) and AVAC. MOH representatives identified sites, key informants and led data collection at country level. AVAC led development of data collection tools, analysis of findings and participated in data collection in Kenya. Advocates in each country organized and led community dialogues with AGYW and, in Zimbabwe, organizations working with AGYW.

In Malawi, ^49^ the rapid assessment was completed in 2020 by representatives from the Department of HIV/AIDS and Department of Reproductive Health at the MOH and Georgetown University Center for Innovation in Global Health to inform the Blantyre Prevention Strategy, launched in May 2020 to catalyze local development of an innovative, data-driven HIV prevention delivery system at the district level. The district of Blantyre was chosen due to the seemingly intractable HIV epidemic and continuing high numbers of new infections there.

### Data collection for rapid assessments

**Figure 2.**
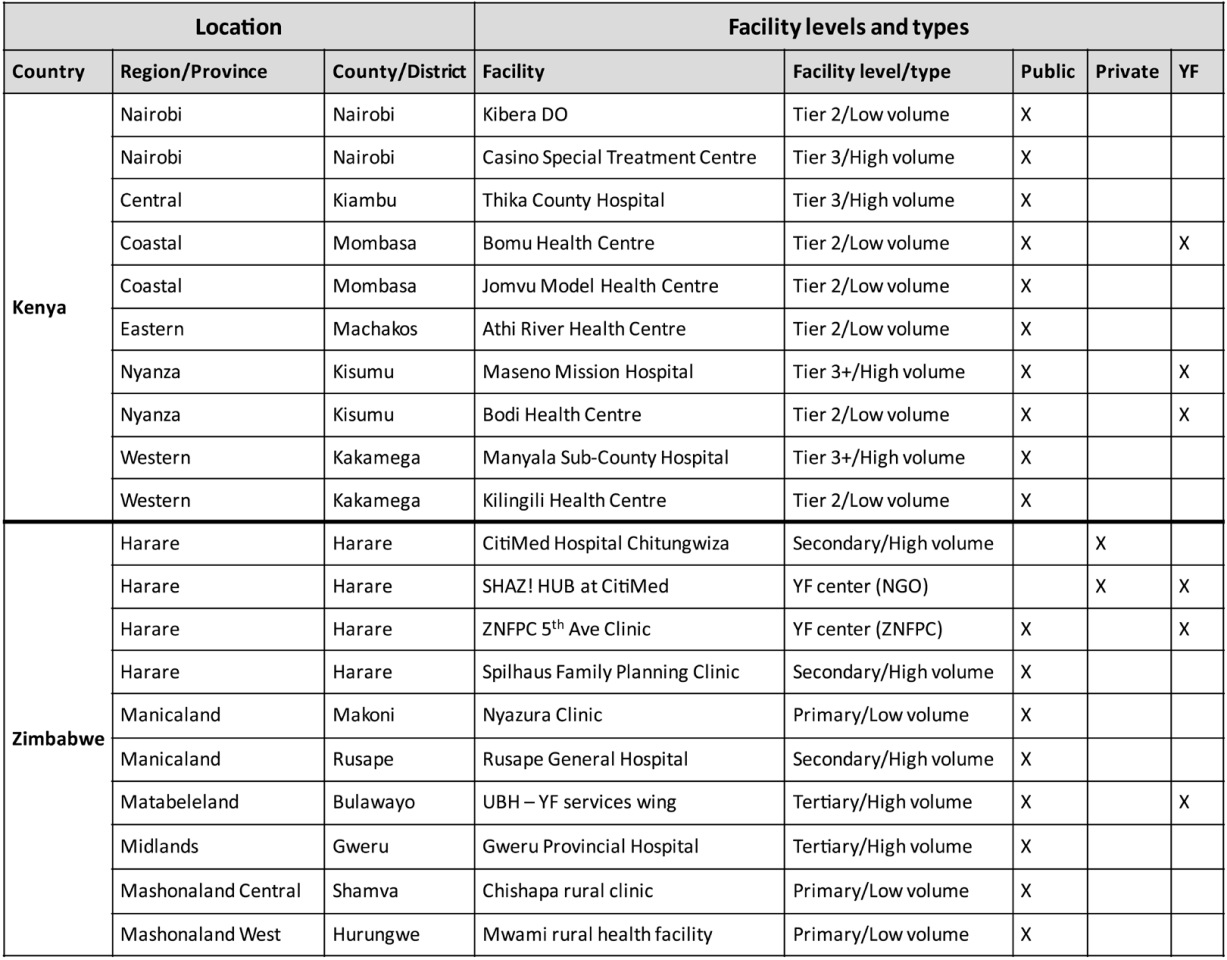
Health facility assessment sites in Kenya and Zimbabwe.

In Kenya and Zimbabwe, research teams identified a cross-section of ten health facilities in each country, primarily in the public sector, across high and low volume sites in both urban and rural settings, including youth-friendly (YF) sites. Twenty health facility assessments were conducted in six regions in Kenya and six provinces in Zimbabwe (see Figure 2).

Key informants were identified from national, sub-national, facility and community levels to provide a representative snapshot of HIV prevention/SRH integration at country level. In total, 73 semi-structured, one-hour interviews were conducted with national, provincial, county and district-level MOH representatives; HIV, SRH and MCH healthcare providers; implementing partners and donors. Six one-day community dialogues were held in-person with AGYW and NGOs. Interviews were requested via email or telephone and health facility visits by letter or email, per MOH protocols. They were primarily conducted in-person and virtually where not feasible, including due to COVID-19 restrictions.

In Malawi, the research team conducted a review of national policies and implementation of HIV and SRH programs in Blantyre district and approximately 70 semi-structured interviews, including with: Blantyre district health officials; NGOs, advocacy organizations and implementing partners; healthcare providers at district health facilities; community-based organizations; organizations associated with the Blantyre Prevention Strategy; U.S. government officials in Malawi and other donors; DREAMS ambassadors and AGYW peer educators. Participants were recruited through organizations and officials associated with the Blantyre Prevention Strategy, NGOs and researchers and community-based organizations with whom they worked. Given travel and social distancing restrictions due to COVID-19, in-person interviews were substituted with interviews through WhatsApp calls to key informants’ cell phones (which are used widely in Malawi), video calls (Zoom or Teams), telephone and email.

Interviews began with an introduction of the researchers and explanation of the research purpose and focus of discussion. Oral informed consent was sought to participate in the interview as well as to audio-record the conversation, where feasible, and confidentiality protocols were explained. Informed consent was then obtained by research teams in each country. Interviews were recorded and notes taken simultaneously, and facility assessment data from Kenya and Zimbabwe were logged in a spreadsheet. Interview transcripts, notes, recordings and health facility assessments were only accessible by the research teams.

### Data analysis for rapid assessments

PMM used standardized interview guides and health facility assessment checklists to conduct the rapid assessment and analysis of data across Kenya and Zimbabwe. The research teams compiled detailed notes after each interview using a standard template, referencing recorded interviews to ensure accuracy and attention to detail, and to document supporting quotes. Health facility assessments collected quantitative data on HIV, FP/SRH and YF services available (e.g., methods and hours offered, whether and how services are integrated, commodity stock-outs, YF accommodations) and staffing and general infrastructure (e.g., facility size, number and cadres of staff across HIV, SRH and YF service areas) to analyze the level of HIV prevention/SRH integration and YF service delivery at each facility. To understand opportunities for and barriers to HIV prevention/SRH integration, each team conducted thematic analysis of detailed notes through a workshop once data collection was completed to identify common themes and outlier perspectives across the interviews, grouped by stakeholder perspectives at the government, health facility and community levels. Findings were triangulated across qualitative and quantitative data.

To understand the spectrum of services in the Blantyre district, the research team analyzed HIV and SRH information and services offered through public sector facilities and youth-friendly health services (YFHS), as well as in the private sector, including national and international NGOs and NGOs with the U.S.-led DREAMS initiative. The analysis highlighted social and structural barriers for sustained and effective use of prevention interventions, including through the education system and gender-based violence (GBV) programs, and new and evolving challenges presented by the COVID-19 pandemic.

### Comparative analysis across countries

To comparatively analyze the three assessments, AVAC and Georgetown University reviewed the final reports of the three assessments to surface patterns, or “themes,” across the assessments and where they differed. Through this review, we identified four cross-cutting, overarching themes around which we structured the comparative analysis: policies and enabling environments, health systems, service delivery and gaps to implementation of HIV prevention/SRH integration. We sorted all pertinent findings from the assessments into these thematic areas, and analyzed similarities and differences in findings, including identifying models of integration. Inconsistencies across the data were contextualized by incorporating additional literature or publicly available country-level sociopolitical data, as well as existing policies from each country, to embed the analysis within a situational context. Findings from the contextual analysis were layered onto the results from the comparative analysis.

### Ethics approval

No ethics approval was sought for this article, as it analyzes the aggregate findings from completed rapid assessments and did not entail additional primary data collection. Ethics approval was not obtained for the rapid assessments, as interviews and health facility asessments collected programmatic data and did not access personal data, and data was used for the purpose of informing national program understanding of the landscapes.

## Results

In this article, we grouped results according to: Enabling Environment and Policy Implementation; Health Systems; Service Delivery and Key Gaps. We highlighted promising national coordination mechanisms, health systems considerations and service delivery approaches to improve HIV prevention/SRH integration, and summarized persistent barriers across countries.

### Enabling environment and policy implementation

Our analyses revealed that the policy environment in all three countries is conducive to expanded integration of HIV prevention and SRH, though most policies on integration preceded oral PrEP rollout (and therefore do not address its integration into SRH services). Operationalizing policies and translating them to local/district levels continues to present challenges, especially in reaching AGYW with YFHS and safe spaces, due in large part to public-sector resource constraints that entrench HIV and SRH silos, rely on referral systems and limit provider training to offer integrated oral PrEP/FP services and deliver them in a non-judgmental, confidential and affordable way.

Sub-national MOH departments for HIV and FP/reproductive health (RH) were cited as critical for cascading operational changes down to facilities, yet the assessments found that they often work in silos, resulting in parallel budgets, workplans and M&E systems. One county RH coordinator in Kenya likened it to *“operat[ing] like water and oil, but this is changing.”* ^50^ The assessments found that structures supporting collaboration across local HIV and FP/RH departments have led to the formation of mechanisms to implement integration, though the extent of these structures varied across countries.

Although policies and strategies on HIV prevention/SRH existed in Kenya, services were not consistently integrated in practice. Prior to the rapid assessment, the government largely had separate programs supporting the operational rollout of SRH and HIV prevention services at national and sub-national levels. ^51^ In response to assessment findings, the MOH built on these existing structures to join HIV prevention and SRH leaders to renew commitment to integration: NASCOP and the Department of Family Health formed a national HIV prevention/SRH integration sub-committee in 2020. The sub-committee analyzed 16 HIV prevention and SRH policies and consulted stakeholders in all 47 counties in Kenya and, based on findings that most policies preceded oral PrEP and required updating to be more comprehensive, produced a policy circular on HIV prevention/SRH integration containing concise directives for county officials and health facilities, and a package of resources to aid implementation. ^52^ Integration pilots are planned in 2021 in five counties.

In Zimbabwe, close coordination across HIV and SRH leadership was noted by key informants to have historically been a strength. Government-led, program-specific (e.g., PrEP) technical working groups (TWGs) are integrated, as are the Prevention Partnership and Adolescent Reproductive and Sexual Health Partnership Forums. ^53^ Importantly, these platforms include representation from implementing partners and potential beneficiaries, including AGYW.

While Malawi has a national-level SRHR and HIV and AIDS integration sub-committee, the assessment found that until recently, separate TWGs at the City and the District level resulted in fragmented programs, with City Councils responsible for urban catchment areas and District Councils responsible for rural ones, with the absence of a forum for routine involvement of community members, including AGYW and other beneficiaries. The establishment of joint City and District TWGs with routine community engagement is an opportunity to coordinate effective integrated services at the district-level. There was broad agreement among key informants in Malawi interviewed that strengthening district-level leadership is key to operationalizing national integration policies, ^54^ consistent with findings in Kenya and Zimbabwe.

*“Coordination is a big challenge… The districts are eager to understand and provide input to the programs that are ongoing, including how targets are set for partners in their district, and they want to be in the lead in coordinating and directing projects and funding, but they lack adequate staffing and bandwidth. With limited time and human resource capacity, it’s difficult for them to engage as much as they might like in supervising partner activities.” -USAID/Malawi official* ^55^

*“Whatever model, we need to strengthen the DHO [District Health Office] -it’s the unit to ensure continuity. Supervision coupled with strengthening the relevant DHO office is what’s needed for the long run.” -University of Malawi, College of Medicine, professor* ^56^

## Health systems

In spite of promising approaches and models showing successful examples of HIV prevention and SRH integration (see Service Delivery section), siloed and resource-constrained health systems were identified as exacerbating barriers to achieving integration.

### Siloed delivery, M&E and supply chain systems

Across countries, HIV prevention and SRH programs are funded, managed and implemented vertically through separate HIV and FP/RH departments within the MOH and delivered in separate physical spaces by different providers in facilities. The assessments found that referrals are commonly used to fill gaps in services offered and alleviate perceived burden on providers, though key informants noted that this can erect barriers for young clients.

*“There is a tendency at facility level to look at HIV services as quite involving. As such, there is a tendency to push and refer, rather than to embrace integration. At the same time, in CCCs [comprehensive care clinics] we are already overwhelmed and yet we are asked to do family planning.” -County AIDS and STI Coordinator (CASCO), Kenya* ^57^

*“For young people it is even harder. You know how difficult it is to be shuffled from one place to another for services. It is disturbing coming to seek reproductive health service[s] then you have to explain your issue over and over to different nurses. It is embarrassing. So they leave without accessing the service. We have missed opportunities.” -MoHCC official, Zimbabwe* ^58^

*“If we are providing PrEP as a hospital but they have to go to OI [Opportunistic Infection, or HIV clinic] to get, it becomes a barrier. You tell them please go to OI, and you give them directions, then you call OI to check if the client has arrived; they don’t get there.” -YF provider, Zimbabwe* ^59^

Our assessments and other studies have found that this siloed service delivery cascade creates barriers to uptake, exacerbates stigma and does not respond to the priorities or multiple vulnerabilities faced by AGYW. ^60– 63^ This system is largely entrenched due to international donor support for a significant portion of these programs, requiring target setting, provider training and staffing, and reporting for HIV prevention and SRH metrics to be done separately. Key informants raised the large imbalance in funding, where HIV is exponentially better-resourced than FP and other SRH services, as a barrier to integration. To illustrate, HIV funding from the United States President’s Emergency Plan for AIDS Relief (PEPFAR) in 2020 was $33 million in Kenya, $177 million in Malawi, and $225 million in Zimbabwe, ^64^ and the Global Fund has allocated $272 million in Kenya, $393 million in Malawi and $425 million in Zimbabwe for HIV from 2020–2022. ^65^ Meanwhile, the combined total funding from UNFPA and the United States Agency for International Development (USAID) for contraceptive commodities across 69 countries was just $200 million in 2019. ^66^ In Kenya, few counties have FP-specific budget lines and external donor investments in FP have decreased over time. ^67^

*“Sometimes the investments of partner-funded programs can lead to less integration. There is this tendency like … me I work with [an implementing partner], I am supposed to do HIV preventive services. MCH is none of my business.” -MCH nurse, Kenya* ^68^

*“There will be a funder who will fund HIV prevention, but won’t fund family planning or reproductive health activities. Or there will be a funder who will fund reproductive health and leave behind issues to do with HIV. For instance, the funder may say I won’t buy test kits for HIV but I will buy contraceptives for young people*. *” -MoHCC official, Zimbabwe* ^69^

As a result, data systems for supply chain management and monitoring and evaluation (M&E) are generally not integrated. ^70^ Siloed supply chain systems for HIV and SRH commodities supporting public sector facilities and pharmacies have been found to be a barrier to integration, ^71^ reinforced by donor funding for separate programs. For example, PEPFAR, the largest HIV donor, currently does not allow funds to be used to procure contraceptive commodities other than condoms, requiring enhanced coordination across programs and donors to deliver both HIV prevention and FP services in the same setting. ^72^

At facility level, HIV prevention and SRH data are collected at different delivery areas and through separate registers, though providers interviewed emphasized that integrating M&E could be an impetus for delivering integrated services *(“if it is not reportable, then it is [perceived by providers to be] unimportant”*). ^73, 74^ Though some indicators may be shared across registers (e.g., Malawi has an integrated register for Antenatal Care and Maternity Delivery, which captures HIV information), patient tracking and monitoring was reported to be unintegrated across sites.

Furthermore, much of the data collected on HIV testing, oral PrEP and specific contraceptive methods are not always available by specific age bands, target populations or service areas, and data completeness and quality are ongoing challenges. ^75, 76^ As one implementing partner in Malawi noted, *“We’re missing out on a lot of data on the SRH entry point, since they [facilities] don’t have to report on those indicators. We’re not capturing how many we’re reaching and what the issues are.”* ^77^ Conducting joint analyses of FP and PrEP data for program-monitoring or decision-making requires special analysis to bridge separate M&E systems, though strategic information officers at MOH-level are also typically siloed. Those working in HIV departments confirmed they cannot readily access FP data and vice versa, and mechanisms in DHIS-2 (e.g., built-in pivot tables and standard reporting functions) do not support integrated analyses. One key informant advocated for M&E systems to be revised to incorporate new indicators on HIV prevention/SRH integration as defined by governments, such as identifying AGYW at risk of HIV through the presence of STIs and early or unintended pregnancy. ^78^

### Healthcare provider capacity to offer integrated services to AGYW

Across countries and studies, healthcare provider capacity-building is underscored as the most critical need for integrating HIV prevention services, including HIV testing and PrEP, in SRH service delivery points. ^79– 81^ Generally, HIV healthcare providers are trained through pre-service education in SRH/FP, but SRH/FP providers lack similar training in HIV services and require training to test for HIV and screen for and provide oral PrEP.

Currently, oral PrEP is primarily delivered by ART nurses and staff specifically certified to prescribe PrEP at HIV service delivery points. In Kenya and Zimbabwe, SRH healthcare providers cited a lack of training as the primary hindrance to providing screening and education about PrEP in SRH settings. ^82, 83^ These providers expressed a lack of confidence to even bring up PrEP during FP counseling or if a high risk for HIV was detected. In Kenya, it was also reported that highly trained FP nurses in the public sector are often transferred to facilities in need of improving service delivery, ^84^ while in Malawi ^85^ and Zimbabwe, ^86^ severe health care worker shortages were underscored as further limiting the number of trained FP providers. Healthcare providers called for greater investment in human resources, including training and supervision, to fill gaps in capacity to integrate PrEP in FP/SRH services.

*“Sometimes a person can come to a facility where there is no one who can provide HIV testing, and end up receiving family planning services only because there was no one who could test her. Too few people are being trained, which leads to clients opting for other services because there is no one who can provide the service they want at the time of visit.” -Nurse, Zimbabwe* ^87^

In addition to a need to strengthen hard skills such as PrEP delivery, this analysis found that negative provider attitudes toward AGYW seeking PrEP and FP services are a key barrier to access in the public sector across countries. In community dialogues in Kenya and Zimbabwe, AGYW described facing stigma from healthcare providers, particularly if they were suspected to be or engaging in sex work or living with HIV, and that privacy and confidentiality are often disregarded, which can demotivate them from returning. In Malawi, the assessment found that sexually-active AGYW, in particular if they are unmarried or have not given birth, are similarly disparaged by providers in public facilities. These findings echo published literature on provider bias toward young people, ^88, 89^ pointing to a need for training on “soft skills” such as YF communication as a component of provider capacity-building.

**Figure 3.**
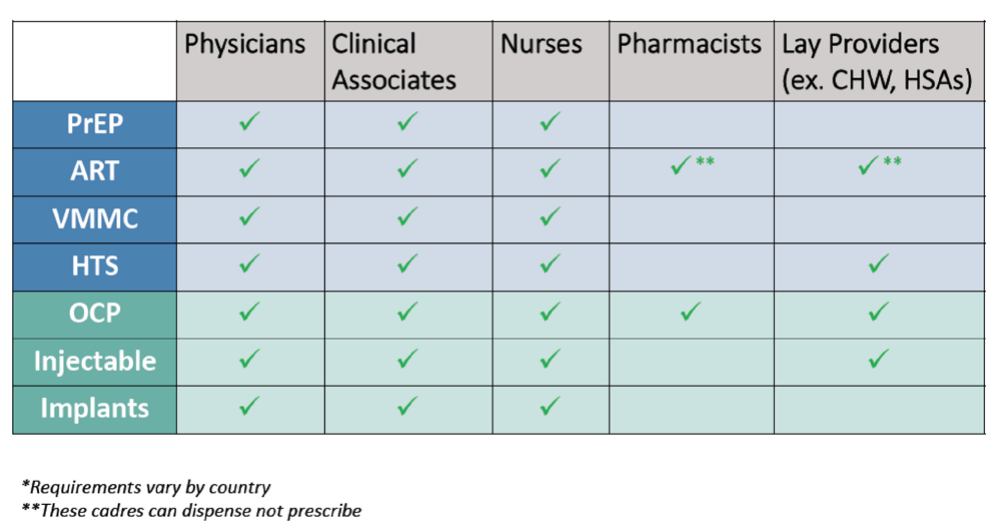
Task-shifting for administering contraceptives & HIV prevention interventions in SSA ^96^.

Task-shifting has been widely employed to expand the number and cadres of providers trained in FP across countries, and is far more advanced compared to oral PrEP (see Figure 3). Task-shifting for PrEP counseling is prevalent in Zimbabwe, where lay providers can counsel on PrEP after completing PrEP-specific trainings, a model other countries where task-shifting for PrEP is inceptive can follow. ^90^ Task-shifting FP to community-based delivery is endorsed in all three countries. It has contributed to notable increases in contraceptive use across countries and in referrals for HIV testing and HIV/STI-related service referrals, and increased dual prevention methods for women living with HIV. ^91, 92^ In Zimbabwe, community health workers (CHWs) can be key to creating demand for FP among AGYW, with CHWs discretely supplying FP methods in rural communities. ^93^ In Malawi, health surveillance assistants (HSAs) provide integrated service delivery at the community level, while community-based distribution agents (CBDAs) provide information and distribute basic FP commodities to young people in communities and provide referrals to facilities for other services. ^94, 95^ Linkages between services in the community and health facilities were often found to be weak for AGYW, indicating a need to expand and integrate the services that CHWs are authorized to provide.

### Limited engagement of the private sector

Leveraging the private sector ^97^ to enhance PrEP uptake is an under-utilized service delivery option, as it is already a well-established channel for FP but has limited HIV prevention delivery across countries. Private sector services are an important source of health care in Kenya, where 34% of modern contraceptive methods are obtained via private providers, including 45% of oral contraceptive pills (OCPs) from pharmacies and 29% of injectables from private hospitals/clinics. ^98^ In Malawi and Zimbabwe, the private sector comprises about one-fifth of delivery channels for FP, ^99, 100^ but access to private practitioners is limited to those who can afford it. NGOs, which are typically subsidized by donor funds, have been important private players in providing integrated SRH and HIV prevention services for AGYW, but donor dependency may impede the sustainability of these channels. ^101^ The Malawian MOH has implemented public-private partnerships (PPPs) to ensure that clients are offered a full package of HIV prevention/SRH services and fill gaps where public sector services are not attracting young people. Government-NGO collaboration in Malawi includes work with Banja La Mtsogolo (BLM), which operates a “nested” approach in 14 sites and public sector strengthening in 29 sites to improve capacity for FP providers at public facilities, especially for LARCs. ^102^ Similarly, in Zimbabwe, the MoHCC is implementing a PPP framework that aims to leverage resources from the public and private sectors to directly reduce the impact of HIV, AIDS and tuberculosis (TB) in the country. ^103^

Pharmacies are a critical channel for FP services in all three countries and an option for expanding HIV prevention commodities such as oral PrEP, once PrEP delivery is permitted. For example, 13.5% of contraceptives are accessed through pharmacies in Zimbabwe ^104^ and OCPs and emergency contraception (EC) are available over-the-counter, but cost and fear of stigma are reported to be major challenges for AGYW. PrEP is also available in pharmacies with a prescription, but stock-outs exist and it remains largely unaffordable. In Kenya, pharmacy-based OCP and PrEP-dispensing is allowed and pharmacies are already offering HIV testing. Kenyatta University, KEMRI and the University of Washington’s Pharmacy-based PrEP initiation implementation study is evaluating pharmacy prescription and refilling of PrEP through oversight of a remote physician and prescriptions offered based on rapid HIV testing. ^105^ Though nascent, these efforts move PrEP delivery toward closer alignment with where many women access FP.

## Service delivery

While integrated service delivery is not widespread across countries, due to a shared gap between policy and practice, Kenya, Malawi and Zimbabwe have employed different approaches to improving services for adolescents and pregnant women that offer lessons for scale up of HIV prevention/SRH integration.

### Youth-friendly health services (YFHS)

Ensuring access to YFHS is a best practice for promoting uptake and effective use of HIV prevention/SRH services among AGYW across SSA. Kenya, Malawi and Zimbabwe have policies that promote the expansion of YF service delivery, yet wide-scale implementation is hindered by limited resources in the public sector.

In Zimbabwe and Kenya, dialogues held with AGYW highlighted major breaches of confidence, discriminatory behavior, lack of respect for life choices and being turned away by healthcare providers when seeking SRH or HIV prevention services. ^106, 107^ In response, AGYW indicated they want guaranteed access to YFHS, defined as the full choice of products, access to free or affordable health services, to be treated with respect when accessing these services and provided with privacy and confidentiality throughout the process.

*“Service providers share your diagnosis in public and point to you where you are supposed to go… In most cases, people are divided according to what brought them there and this is not done in private e.g. ‘those with HIV come on this side!.’ [I]n order to avoid such humiliation, AGYW prefer to go to outside [of their locality].” -AGYW, Kenya* ^108^
*“Here (at a youth drop-in centre) I’m treated well. The staff is friendly and I meet other young people my age. I’m on PrEP and no one asks me why I need PrEP, like what have I done that warrants you taking PrEP.” -AGYW, Zimbabwe* ^109^

**Figure 4.**
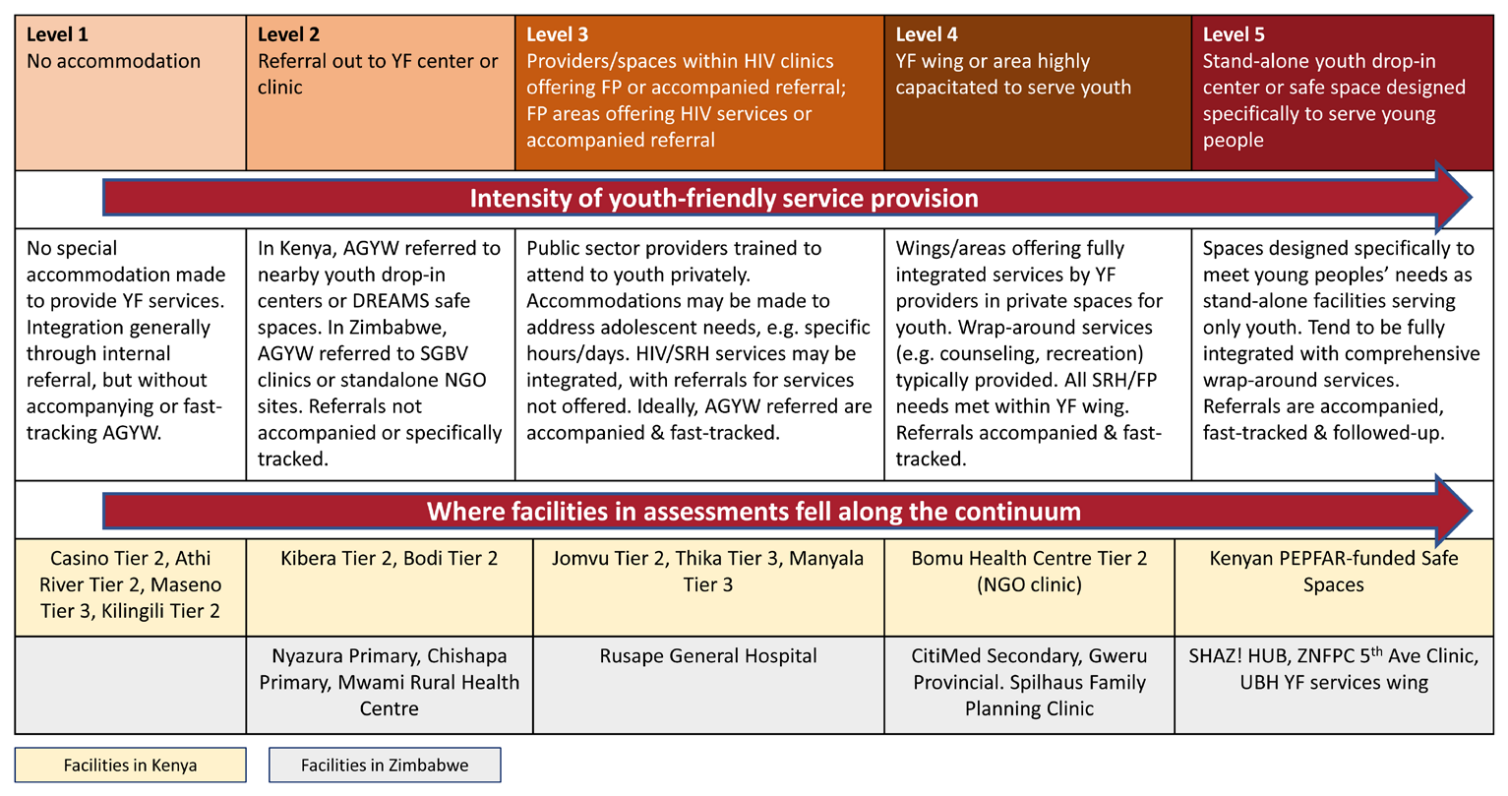
The integration-youth friendly (YF) continuum.

Health facility assessments in Kenya and Zimbabwe found that where available, YFHS provide the strongest examples of comprehensive, integrated HIV prevention/SRH service delivery for adolescents and young people, and were found to mitigate stigma and increase access. Integration and YFHS were found to move together along a continuum, with more YF models exhibiting a greater level of HIV prevention/SRH integration (see Figure 4). ^110, 111^ Low-volume facilities tend to be less YF and are integrated by default due to limited number of staff. High-volume sites tend to offer HIV and FP services in separate areas and rely on referrals to deliver comprehensive services. YF sites typically offer fully, intentionally integrated HIV prevention/SRH and other services specifically for AGYW; these are often highly supported by implementing partners and donors, which equips them with additional resources and incentivizes meeting AGYW-focused targets. In the facility assessment in Kenya, facilities were generally less integrated and YF: four facilities fell in level 1, the least integrated/YF category; two facilities in level 2; three facilities in level 3; one facility in level 4 and PEPFAR-funded safe spaces represent level 5, the most integrated/YF level. In Zimbabwe, greater levels of integration and youth-friendliness were found, with no facilities found in level 1; three facilities in level 2; one facility in level 3; three facilities in level 4 and three facilities in level 5.

In Kenya, YFHS in the public sector are primarily available through stand-alone youth clinics and YF rooms or corners, where HIV testing, PrEP and FP are available. ^112, 113^ YFHS are not widely embedded in public SRH units and primary health facilities and will require equipping facilities with YF corners and training healthcare providers. By contrast, Zimbabwe ^114– 116^ and Malawi ^117^ have moved toward integrating YFHS in public sector facilities, a model that is less expensive than stand-alone clinics and theoretically offers YFHS at all service delivery points, including FP/SRH, but availability of oral PrEP is limited. Further, access barriers remain: YFHS are sometimes only available during certain days or times, ^118, 119^ and providers reported that AGYW with children are often referred to general primary care, ^120^ a missed opportunity to provide them with YFHS. Despite a recognition of the need for specific services for AGYW, the reach of YFHS remains very limited in Malawi. ^121^ Since the implementation of the YFHS program began in 2007, one comprehensive evaluation has been conducted to assess program coverage. The evaluation revealed that only half of CBDAs and 64% of peer educators had been trained in YFHS, including counseling on contraception and HIV/AIDS, and only 68% of health center providers had been trained to offer YFHS.

Across countries, YFHS supported by implementing partners are typically well-resourced “one-stop shops” or safe spaces for integrated HIV prevention/SRH services, often with layered programming aimed at empowering AGYW (e.g., peer clubs and social asset-building). ^122– 125^ In our assessments, these sites showed the highest level of HIV prevention/SRH integration and high utilization by AGYW. While most studies on YFHS in the published literature focus on either SRH or HIV prevention service utilization and outcomes – rather than on integrated services or comprehensive HIV prevention/SRH outcomes ^126^ – studies of YFHS for HIV have shown improvements among adolescents living with HIV around adherence and retention, ^127, 128^ as well as uptake of HIV testing and PrEP for those who qualify. ^129, 130^

### Integrating PrEP delivery into PMTCT programs

Successful integration of PMTCT with MCH was supported by investments in human resources (e.g., training, task-shifting and hiring additional staff), largely from HIV donors, but the assessments found that similar investments have not been allocated to integrating other HIV prevention and SRH services.

While primary HIV prevention among women of reproductive age and prevention of unintended pregnancies is an established prong of PMTCT programs, most countries have emphasized the prong related to preventing vertical transmission. ^131^ Kenya, Malawi and Zimbabwe have integrated PMTCT and SRH, where HIV testing and ART are routinely provided to pregnant and breastfeeding women (PBFW) upon HIV diagnosis during antenatal care (ANC) and post-partum visits. ^132^ However, there is a critical gap in primary prevention for PBFW who test negative for HIV during ANC.

Malawi was the first country to pioneer Option B+ in late 2011, which offered all HIV-infected PBFW free, lifelong ART, regardless of CD4 count or clinical stage. Option B+ demonstrated the impact of integrated HIV services, especially for women living with HIV – an important advance recognized in the WHO global guidance. ^133^ Kenya and Zimbabwe adopted Option B+ in 2012 and 2013, respectively, and by 2019 all three countries achieved high HIV testing rates and with ART coverage exceeding 90 percent. ^134^ Despite significant progress, vertical transmission rates remain higher than expected, ^135^ due in part to new infections among women who had tested negative in early pregnancy, highlighting the need for early and repeated ANC visits, with repeated HIV testing throughout ANC, and ensuring PrEP provision for women who test negative. ^136^ Adolescent PBFW tend to have much lower rates of PMTCT and ANC uptake and face numerous challenges, including stigma from providers and communities and lack of social support. ^137– 139^

Although integrating ART and ANC services has contributed to reduced infections among children, preventing primary HIV infections among women has not been prioritized. In response, national strategic HIV plans in all three countries have underscored the need to address gaps in the unmet need for FP among women living with HIV, scale up PMTCT sites and services in general and ensure they are adolescent-friendly, strengthen community mobilization and community-based support systems for retention and improve quality of care in integrated SRH, HIV, TB and RMNCAH services. ^140– 142^

By extension, oral PrEP rollout for PBFW has been slow due to limited safety data in this population until more recently and from lagging HIV prevention services for this population. Oral PrEP is primarily offered in HIV clinics, contributing to low uptake in the absence of reaching women via SRH channels, which tend to be preferred and accessed by more women. In Kenya, Comprehensive HIV Care Clinics (CCCs) and DREAMS safe spaces are the primary delivery channels of PrEP for AGYW. ^143^ With the exception of implementation research studies, ^144, 145^ PrEP is not offered in SRH services, and integration of PrEP is instead typically achieved through referral. Similarly, in Zimbabwe, while FP is well-integrated into HIV services, Opportunistic Infection (OI, or HIV treatment) clinics are the primary delivery channels for PrEP. SRH units rarely provide PrEP or offer referrals for PrEP services, except for pilot Zimbabwe National Family Planning Council (ZNFPC) sites ^146^ and YF facilities. ^147^ Until 2021, PrEP had been provided in Malawi in limited scope through NGOs, ^148^ but public sector clinics have begun to deliver PrEP to high-risk adolescents, and plan to scale up. Assessments found that clients are being lost when referred for PrEP, indicating that referrals fall short of meeting women’s needs. Still, SRH provider capacity and infrastructure to provide additional PrEP services remains limited (see Health Systems section above).

Despite the remaining challenges, significant increases in ART coverage for women have been propelled by integrating ART into the points where women traditionally access health services. This was spurred by task-shifting, simplified delivery and linkages to community-based services and accelerated donor support – elements that could better integrate oral PrEP into broader SRH programs.

## Key gaps

### Targeting AGYW with demand generation

This analysis found that demand generation activities for both HIV prevention and SRH to reach AGYW have not been scaled, despite supportive policies. ^149, 150^ One implementing partner in Malawi emphasized, *“One of the most important things is that we need an avenue to reach girls, we need to have activities that matter to them (social assets, economic strengthening), where they can meet and we can bring services to them.”* ^151^ Mobilizing demand for HIV prevention and SRH by providing information for AGYW, as well as for their partners, peers and communities, who are influential in their decision-making, was cited as necessary to increase access to services and combat stigma. ^152^

*“Once the community spots you going behind the building [to the health facility], anyone can guess what you are there for.” -AGYW, Kenya* ^153^
*“The girls and women show real fear when you mention clinics and FP... You will even be forbidden from taking part in church activities just for going to a clinic.” -AGYW, Zimbabwe* ^154^
*“Outreach is more than giving out a flier. You need to go to high risk areas and have people who know how to have conversations with their peers to convince them to come in.” -Researcher, Malawi* ^155^

Demand generation strategies across countries rely on platforms and interlocutors whom AGYW trust, including youth or girls’ clubs, peer education or outreach activities (e.g., DREAMS ambassadors), as well as school-based activities. ^156^ These programs have demonstrated some successes in reaching AGYW in different age groups and different contexts with information about SRH and HIV prevention in accessible, non-stigmatizing ways, often through peer-led activities and community-based interventions. According to USAID, in 2020, DREAMS reached over 1.6 million AGYW with prevention services. ^157^ These strategies include education for 9-14 year-old boys and girls on primary prevention, complemented by outreach to their families and communities to bolster support for adolescents. DREAMS ambassadors, in particular, play a key role in linking AGYW to economic strengthening interventions, ^158^ as well as for gender-based violence (GBV), PrEP and other services. ^159^ The DREAMS program has seen an increase in PrEP uptake among AGYW in DREAMS districts. ^160^

Although strong programs exist to reach AGYW, there are generally low levels of information about HIV prevention in communities, and knowledge of HIV prevention hovers around 50% for AGYW across countries. ^161^ Coupled with myths and misconceptions on side effects and impact on fertility, this lack of and mis-information engenders fear and hinders social support for AGYW to use HIV prevention and SRH services. ^162^

Strategies to reduce unmet need or create demand tend to focus on specific FP or HIV prevention products (e.g., PrEP, long-acting contraception ^163^), rather than comprehensively considering integrated needs across all existing HIV prevention and FP options for AGYW. Moreover, demand generation is not typically well-funded nor integrated, so individual programs only provide information about certain services, reinforcing silos between HIV prevention and SRH. ^164^ In Zimbabwe and Kenya, HIV communication strategies that promote integrated messages have been developed, but implementation remains under-resourced. ^165, 166^ Key informants across countries underscored that demand generation will require dedicated investments from donors and governments to be effectively implemented.

### Structural environment

Integrating HIV prevention and SRH services is one approach to better meet the needs of AGYW. Though largely outside the scope of these assessments, structural barriers – including gender inequalities and discriminatory cultural norms – compound their risks of HIV, unintended pregnancy, STIs and early marriage, and prevent access to economic and educational opportunities.

GBV increases HIV risk, and women and girls who experience violence are at greater risk for HIV. ^167^ Kenya, Malawi and Zimbabwe have recognized that addressing GBV is critical to their HIV responses. ^168^ Data from recent Violence Against Children Surveys (VACS) show high rates of violence across countries: in Kenya, 16% of girls experience sexual violence and 39% experience physical violence before age 18; ^169^ in Zimbabwe, 9% of girls experience sexual violence and 17% physical violence prior to age 18 ^170^ and in Malawi, one in five girls is sexually abused before age 18. ^171^ In Kenya and Zimbabwe, higher HIV prevalence rates were found among women who experienced childhood violence, underscoring the link between violence and HIV risk.

In Malawi and Zimbabwe, dedicated facilities exist that address GBV and offer integrated HIV prevention/SRH services. In Malawi, “Chikwanekwanes” (literally, “everything under one roof”) provide medical, legal and psychosocial services for survivors of sexual violence, including HIV testing, post-exposure prophylaxis (PEP) and follow-up testing, STI management and EC when indicated. ^172^ In Zimbabwe, Sexual Gender-Based Violence (SGBV) clinics, also known as “one-stop centres,” provide PEP, FP, counseling and YFHS in seven districts (in addition to psychosocial, legal and police support), though PrEP is not offered. In 2020, the United Nations Population Fund (UNFPA) allocated an additional $2.5 million to the GBV response in Zimbabwe, which included scaling up mobile one-stop centres to bring integrated, free services into communities, pointing to the value of integrating health and social services to reach those most at-risk. ^173^

## Discussion

The changing funding, policy, research and development landscape highlights the growing urgency and commitment needed for HIV prevention and SRH integration. Both PEPFAR and the Global Fund to Fight AIDS, TB, and Malaria (Global Fund), the largest funders of HIV programs globally, have recognized the importance of HIV prevention/SRH integration for AGYW, and have increased their support for expanded access: PEPFAR allocated $17.5 million for oral PrEP in 2018 and $35.7 million in 2020, as well as a $1 billion investment in the DREAMS program, and the Global Fund allocated $140 million through the HER Initiative. ^174– 176^ Both initiatives show that layered social, structural and biomedical interventions – especially using YF approaches – can achieve impact for AGYW. ^177^ Current PEPFAR and Global Fund country plans prioritize bi-directional integration by providing FP and STI testing within HIV programs, integrating oral PrEP across service delivery points, including ANC, ART, STI and FP services, and capacity-building for FP/MCH providers to supply oral PrEP. Making these changes now to expand access to oral PrEP also ensures greater access to HIV testing and other forms of HIV prevention (e.g., condoms, risk-reduction counseling) and will lay the foundation for expanding access to all biomedical prevention once new products enter the market.

A focus on delivering YFHS is critical to expanding access to integrated HIV prevention/SRH services for AGYW. Data from DREAMS shows that new HIV infections among AGYW have been reduced in all the geographic settings where it operates: 96% of DREAMS districts saw a reduction of at least 25% and 62% saw a reduction greater than 40%. ^178^ In 2019, the Global Fund reached over 1 million young people between ages 10-24 with HIV prevention programs in Malawi, over 54,000 in Kenya and over 29,000 in Zimbabwe. ^179^ DREAMS has broad reach across countries, serving 257,000 AGYW ages 10-24 in Kenya, with nearly 9,000 taking up oral PrEP; 185,403 AGYW in Zimbabwe, of which 2,000 initiated oral PrEP and nearly 60,000 AGYW in Malawi, ^180^ with oral PrEP rollout just beginning. All countries have plans for increasing the number of AGYW reached and for integrating oral PrEP into these programs in 2021.

More integrated donor-funded programs would bolster integration and YF efforts downstream at the point-of-care where it matters most. Unified donor targets could ensure that HIV prevention and SRH are given greater balance by programs and healthcare providers, and that providing YFHS in these settings is prioritized. Zeroing in on specific programmatic elements of DREAMS and Global Fund programs that can be adapted by the public sector could help scale up YFHS. ^181^ To measure variables that may have a greater impact on the use of services by young people, such as confidentiality, privacy and accessibility of quality services, a core set of indicators must be identified. ^182^ Conducting more rigorous studies using a refined set of indicators that directly assess the benefit of delivering integrated services on both HIV prevention and SRH outcomes is critical to measure and compare the impact and effectiveness of YFHS, compared to the standard of care. While many are donor-funded, understanding which aspects of YFHS are most impactful and valued by AGYW can inform approaches to integration in the public sector; ^183– 186^ operational research evaluating the effects of these models on uptake of both HIV prevention and SRH services is needed.

Dedicated structures are required to support implementation of integration throughout the health system. Updating outdated national integration policies to include PrEP can mandate programs to deliver PrEP products as part of the package of integrated services. Cascading these policies to sub-national HIV and FP programs and down to facilities – with appropriate resourcing, coordination mechanisms and training – will help to operationalize them. Leadership by sub-national HIV and FP program coordinators is fundamental to drive development and execution of integrated workplans, budgets and meetings and to provide oversight to and monitor implementation at facilities. Kenya’s HIV/SRH integration sub-committee, described above, is a model other countries can emulate to move the needle.

Health systems adaptations will also be needed to effectuate integration. Revising and strengthening M&E systems to document the provision of HIV prevention services in SRH service areas and vice versa, and integrating systems up the chain to national level, would enhance the ability to look at data in an integrated fashion. Electronic monitoring systems, currently being rolled out in Kenya, Malawi and Zimbabwe, provide an opportunity to track individual client health records across services and facilities. Data should be made available in real time and disaggregated by age to improve targeting of at-risk AGYW for evidence-based decision-making. Bi-directional integration of data systems can also support the integrated management of PrEP and FP commodities.

While health systems changes can take time, there are interim measures that health facilities can adopt to facilitate integration and support access for AGYW. These include: “whole site training,” where administrative and clinical staff alike are sensitized on FP, HIV testing and PrEP services and where to access them; accompanied referral for AGYW to minimize loss to follow-up; fast-tracking young people in queues; displaying information, education and communication (IEC) materials on SRH and HIV prevention in all service areas; extending hours that services are available, particularly to afternoons and weekends, which may be more convenient for young people; allocating staff according to daily client flow and employing health systems navigators and peer educators, who may be preferred messengers for AGYW, to conduct counseling, escort clients to services and follow up on referrals, thereby offloading these tasks from provider workloads. ^187– 193^

Training providers on the basics of oral PrEP and on screening high-risk individuals for eligibility would vastly improve the capacity of providers in SRH service areas to identify and refer potential clients for oral PrEP. Task-shifting for HIV testing and oral PrEP delivery could expand opportunities to offer combination prevention alongside SRH services. Similarly, task-shifting for contraceptive commodities, including long-acting reversible contraceptives (LARCs) and the self-injectable contraceptive, subcutaneous depot medroxyprogesterone acetate (DMPA-SC), could broaden access for at-risk women and girls. Diffusing the capacity to provide services across staff could be perceived as alleviating nurses’ workloads, while offering a more comprehensive HIV prevention/SRH package. A systematic review of evidence from low- and middle-income countries found task-shifting led to cost savings and efficiency gains across settings and health areas. ^194^

The need for capacity-building and building empathy among providers is evident to mobilize AGYW to access SRH and HIV prevention services. ^195^ Providers often recognized the benefits of HIV prevention/SRH integration for clients even if it increased workloads in the initial stages of integration. Strengthening soft skills, such as training on values clarification and person-centered care, must be coupled with clinical training to address negative provider attitudes that often dissuade AGYW from seeking services, particularly in public facilities. ^196, 197^ Mentorship for providers that includes skills-building on integrated services has increased uptake, range of services offered, provider self-confidence and quality of care in resource-constrained settings. ^198^ Supportive managerial supervision that empowers providers to make decisions and fosters teamwork has improved delivery of integrated services and helped staff to surmount structural issues, such as stock-outs or workload distribution. ^199^

There are also critical opportunities to expand access to integrated services through the private sector, particularly through pharmacies, which are a critical channel for FP services in all three countries and have major potential for expanding oral PrEP. In many resource-constrained settings, retail pharmacies fill an important gap in the health care system, providing access to treatment of urgent conditions, monitoring of chronic conditions, point-of-care testing and preventative care, ^200– 204^ and have been shown to increase access for young people. ^205^ Delivery of oral PrEP through pharmacies is utilized in the US, Europe and Asia, and studies have shown that oral PrEP can be successfully provided completely by pharmacists in these settings, with oversight by a remote physician. ^206^ A recent stakeholder consultation in Kenya showed that providers and implementers were strongly supportive of developing and testing a model for pharmacy-based oral PrEP delivery to increase oral PrEP access. ^207^ The consultative group developed a pathway for pilot testing pharmacy-based oral PrEP delivery in Kenya. Expanding PrEP delivery beyond health clinics and aligning FP and HIV prevention delivery channels has the potential to increase uptake of both PrEP and contraception, and respond to women’s desire for convenient and less stigmatized services.

A growing number of HIV prevention products – including a vaginal ring, injectable PrEP and multi-purpose prevention technologies – are pending regulatory approval and will be available in the next two to three years. Aligning the delivery channels where these products will be offered with contraception or as part of ANC visits would likely make access much easier for AGYW. Integrated services are more convenient, treat women holistically and reduce the burden placed on AGYW to access healthcare that meets their multiple and changing needs. Several studies have shown that when oral PrEP is co-delivered alongside FP, uptake of both increases. ^208, 209^ Accordingly, introducing new products for HIV prevention within integrated services could increase their uptake and continued use. FP healthcare providers are more familiar with administering a range of modalities, from pills to injections to vaginally inserted products, compared to HIV healthcare providers. FP healthcare providers also interact more frequently with women over a lifetime and, with adequate training and support, could be well-placed to address multiple health concerns. Advancing HIV prevention and SRH integration now could lay the groundwork for the faster and more equitable introduction of a range of technologies in the future. Putting AGYW at the center of care demands acting on commitments to HIV prevention/SRH integration. Most importantly, AGYW are calling for services that are respectful, comprehensive, educational and empathetic, not patronizing or fragmented. ^210, 211^

Shared needs identified through rapid assessments in Kenya, Malawi and Zimbabwe were greater investments in provider capacity-building and demand generation to expand integrated service delivery, while country-specific needs for integration centered on health systems adaptations, donor support and scope of PrEP implementation. Further research is required to: 1) identify which interventions and delivery models are preferred by AGYW and correlated with better HIV and SRH outcomes; 2) determine the cost-effectiveness of integrating HIV prevention into SRH facility- and community-based delivery channels, as well as expanding private sector access of PrEP and SRH products through pharmacies and 3) pinpoint effective strategies for capacitating and supporting healthcare providers at the frontlines of delivering integrated care.

## Conclusions

While the call for HIV/SRH integration is not new, it has been implemented more successfully for HIV treatment and SRH, while integration of HIV prevention with SRH has lagged. Although integrated policies exist on paper, HIV prevention and SRH services remain siloed and these policies preceded the introduction of new biomedical interventions such as oral PrEP. With the introduction of HIV prevention products expected in the next couple of years, there is an urgency to ensure that policies on integration are updated and that programs build on lessons where HIV prevention and SRH integration has been successful. In Kenya, Malawi and Zimbabwe, YFHS and PMTCT programs offer the strongest examples of integration of HIV prevention and SRH that can be leveraged and expanded to more effectively reach AGYW.

Supply and demand-side challenges to HIV prevention/SRH integration remain. These include: provider capacity-building, synchronization of HIV and SRH services and M&E and supply chain management systems, demand generation and structural barriers. Renewed donor and government efforts to fund and systematically address these are warranted if integration is to be realized at scale.

Investing now in integrating HIV prevention and SRH will have synergistic effects, such as improving early and repeated ANC visits, streamlined SRH supply chains, expanding YFHS to the public sector and increasing access to PrEP and HIV testing by diversifying delivery channels, all of which will alleviate pressure in the future. In addition, it will prepare programs for a more complex prevention landscape and make systems more resilient to an inevitable decrease in external funding. Prioritizing integration is critical to meet the needs of AGYW and put them at the center of response while also creating efficiencies for the health system in the long-term.

## Data availability

### Underlying data

Data underlying the results are available as part of the article and no additional source data are required. The assessments in Kenya, Malawi and Zimbabwe that are comparatively analyzed in this article are accessible via the following sources:

- *Integration of HIV prevention and sexual and reproductive health services in Kenya* is available at https://www.avac.org/resource/integration-hiv-prevention-and-sexual-reproductive-health-services-kenya.
- *Integration of HIV prevention and sexual and reproductive health services in Zimbabwe* is available at https://www.avac.org/resource/integration-hiv-prevention-and-srh-services-zimbabwe.
- *Opportunities and Challenges for the Integration of HIV and SRH Services in Malawi* is currently unpublished and available upon request under restricted access. To request access to this assessment, please email Sara Allinder at Sara.Allinder@georgetown.edu.

## Footnotes

^1^ UNAIDS: AIDSinfo. 2020. Accessed May 14, 2021. Reference Source

^2^ AVAC: Understanding the Results of the ECHO Study. 2020. Reference Source

^3^ UNAIDS: AIDSinfo. 2020. Accessed May 14, 2021. Reference Source

^4^ UNAIDS: AIDSinfo. 2020. Accessed May 14, 2021. Reference Source

^5^ UNFPA: State of the World Population 2020. 2020. Reference Source

^6^ UNFPA: World Population Dashboard. 2021. Accessed May 14, 2021. Reference Source

^9^ FP2020: Zimbabwe: FP2020 Core Indicator Summary Sheet: 2019–2020 Annual Progress Report. 2020. Reference Source

^10^ AVAC: The Global PrEP Tracker. 2021. Accessed May 14, 2021. Reference Source

^11^ Kenya National Bureau of Statistics: Kenya Demographic and Health Survey 2014. 2015. Reference Source

^12^ Ministry of Health, National AIDS & STI Control Programme: Status on implementation of Oral Pre-exposure Prophylaxis for HIV in Kenya. Nairobi: NASCOP. 2018.

^13^ Zimbabwe National Statistics Agency and ICF International: Zimbabwe Demographic and Health Survey 2015: Final Report. Rockville, Maryland: Zimbabwe National Statistics Agency (ZIMSTAT) and ICF International. 2016. Reference Source

^14^ National Statistical Office (NSO) [Malawi] and ICF: Malawi Demographic and Health Survey 2015–16. Zomba, Malawi and Rockville, Maryland, USA: NSO and ICF. 2017. Reference Source

^15^ UNAIDS: AIDSinfo. 2020. Accessed December 8, 2021. Reference Source

^16^ UNAIDS: AIDSinfo. 2020. Accessed December 8, 2021. Reference Source

^17^ UNFPA: Adolescents and Youth Dashboard. 2020. Accessed December 8, 2021. Reference Source

^18^ MacQuarrie, KLD: HIV/AIDS and Unmet Need for Family Planning. DHS Working Papers No. 122. Rockville, Maryland: ICF International. 2015. Reference Source

^19^ McCoy SI, Buzdugan R, Ralph L, *et al.*: Unmet need for family planning, contraceptive failure, and unintended pregnancy among HIV-infected and HIV-uninfected women in Zimbabwe. *PLoS One.* 2014; **9**(8): e105320. PubMed Abstract | Publisher Full Text

^20^ Rucinski KB, Powers KA, Schwartz SR, *et al.*: Longitudinal patterns of unmet need for contraception among women living with HIV on antiretroviral therapy in South Africa. *PLoS One.* 2018; **13**(12): e0209114. PubMed Abstract | Publisher Full Text

^21^ Abubeker FA, Fanta MB, Dalton VK: Unmet Need for Contraception among HIV-Positive Women Attending HIV Care and Treatment Service at Saint Paul’s Hospital Millennium Medical College, Addis Ababa, Ethiopia. *Int J Reprod Med.* 2019; **2019**: 3276780. PubMed Abstract | Publisher Full Text

^43^ HIV Prevention Market Manager, Ministry of Health [Kenya]: Integration of HIV prevention and sexual and reproductive health services in Kenya. 2020. Reference Source

^45^ Georgetown University Center for Innovation in Global Health: Opportunities and Challenges for the Integration of HIV and SRH Services in Malawi. 2020. Unpublished report.

^46^ MEASURE Evaluation: Routine Health Information System Rapid Assessment Tool: Implementation Guide. 2018. Reference Source

^47^ HIV Prevention Market Manager, Ministry of Health [Kenya]: Integration of HIV prevention and sexual and reproductive health services in Kenya. 2020. Reference Source

^48^ HIV Prevention Market Manager, Ministry of Health and Child Care [Zimbabwe]: Integration of HIV prevention and sexual and reproductive health services in Zimbabwe. 2020. Reference Source

^49^ Georgetown University Center for Innovation in Global Health: Opportunities and Challenges for the Integration of HIV and SRH Services in Malawi. 2020. Unpublished report.

^50^ Interview with RH coordinator, Kenya, Nov. 2019.

^51^ Interview with MOH officials, Kenya, Nov. 2019.

^52^ On file with the national HIV/SRH integration sub-committee, Ministry of Health, Kenya.

^53^ HIV Prevention Market Manager, Ministry of Health and Child Care [Zimbabwe]: Integration of HIV prevention and sexual and reproductive health services in Zimbabwe. 2020. Reference Source

^54^ Interviews with key informants in Malawi, March-April 2020.

^55^ Interview with USAID/Malawi official, March 2020.

^56^ Interview with University of Malawi, College of Medicine, professor, April 2020.

^57^ Interview with County AIDS and STI Coordinator (CASCO), Kenya, Nov. 2019.

^58^ Interview with MoHCC official, Zimbabwe, March 2020.

^59^ Interview with YF provider, Zimbabwe, March 2020.

^60^ Smit JA, Church K, Milford C, *et al.*: Key informant perspectives on policy- and service-level challenges and opportunities for delivering integrated sexual and reproductive health and HIV care in South Africa. *BMC Health Serv Res.* 2012; **12**: 48. PubMed Abstract | Publisher Full Text

^63^ Kangudie DM, Guidigbi H, Mensah S, *et al*.: Effective integration of sexual reproductive health and HIV prevention, treatment, and care services across sub-Saharan Africa: where is the evidence for program implementation? *Reprod Health*. 2019; **16**(Suppl 1): 56. PubMed Abstract | Publisher Full Text

^64^ amfAR: PEPFAR Country/Regional Operating Plans (COPs/ROPs) Database. Accessed May 25, 2021. Reference Source

^65^ The Global Fund: The Global Fund Data Explorer. Accessed May 25, 2021. Reference Source

^66^ CHAI, Reproductive Health Supplies Coalition: Family Planning Market Report. 2020. Reference Source

^67^ Ministry of Health [Kenya]: National Family Planning Costed Implementation Plan 2017-2020. Nairobi: Ministry of Health. 2017. Reference Source

^68^ Interview with registered MCH nurse, Kenya, Nov. 2019.

^69^ Interview with MoHCC official, Zimbabwe, May 2020.

^70^ Dehnavieh R, Haghdoost A, Khosravi A, *et al.*: The District Health Information System (DHIS2): A literature review and meta-synthesis of its strengths and operational challenges based on the experiences of 11 countries. *Health Inf Manag.* 2019; **48**(2): 62–75. PubMed Abstract | Publisher Full Text

^71^ Inter-Agency Task Team for the Prevention and Treatment of HIV Infection in Pregnant Women, Mothers and Children: HIV and Sexual and Reproductive Health Programming: Innovative Approaches to Integrated Service Delivery. 2014. Reference Source

^72^ USAID. Family Planning and HIV Integrated Supply Chains. Reference Source

^73^ Interview with healthcare providers, Zimbabwe, March 2020.

^74^ Interview with implementing partner, Kenya, Nov. 2019.

^75^ Hagel C, Paton C, Mbevi G, *et al.*: Data for tracking SDGs: challenges in capturing neonatal data from hospitals in Kenya. *BMJ Glob Health.* 2020; **5**(3): e002108. PubMed Abstract | Publisher Full Text

^76^ Njeru I, Kareko D, Kisangau N, *et al.*: Use of technology for public health surveillance reporting: opportunities, challenges and lessons learnt from Kenya. *BMC Public Health.* 2020; **20**(1): 1101. PubMed Abstract | Publisher Full Text

^77^ Interview with implementing partner, Malawi, April 2020.

^78^ Interview, Malawi, March 2020.

^79^ Interview with healthcare providers, Kenya, Nov. 2019.

^81^ Interview with healthcare providers, Zimbabwe, March 2020.

^82^ Interview with healthcare providers, Kenya, Nov. 2019.

^83^ Interview with healthcare providers, Zimbabwe, March 2020.

^84^ Interview with implementing partners, Kenya, Nov. 2019.

^85^ Republic of Malawi, Ministry of Health: National Sexual and Reproductive Health and Rights (SRHR) Policy. Lilongwe: Ministry of Health. 2016.

^86^ HIV Prevention Market Manager, Ministry of Health and Child Care [Zimbabwe]: Integration of HIV prevention and sexual and reproductive health services in Zimbabwe. 2020. Reference Source

^87^ Interview with registered nurse, Zimbabwe, March 2020.

^88^ Starling S, Burgess S, Bennette N, *et al*.: Beyond Bias. 2017. Reference Source

^89^ Jonas K, Crutzen R, van den Borne B, *et al*.: Healthcare workers’ behaviors and personal determinants associated with providing adequate sexual and reproductive healthcare services in sub-Saharan Africa: a systematic review. *BMC Pregnancy Childbirth.* 2017; **17**(1): 86. PubMed Abstract | Publisher Full Text

^90^ Fitch L, Rodrigues J, Warren M: How policy affects practice: policy barriers to provision of HIV biomedical prevention services in Sub-Saharan Africa. 2020. Reference Source

^91^ Olawo AA, Bashir I, Solomon M, *et al.*: “A cup of tea with our CBD agent … ”: community provision of injectable contraceptives in Kenya is safe and feasible. *Glob Health Sci Pract.* 2013; **1**(3): 308–315. PubMed Abstract | Publisher Full Text

^92^ Extending Service Delivery (ESD) Project, USAID: Extending Service Delivery Project: Best Practice Brief # 1: Integrating HIV Services in Local Family Planning: The Expanded Community-Based Distribution Model and Zimbabwe Experience. Washington, DC: ESD Project, USAID. Reference Source

^93^ Rapiti R, Mullick S: An evaluation of Population Services Zimbabwe’s mobile outreach model. Nairobi: Population Council. 2014. Reference Source

^94^ Interview, Malawi, April 2020.

^95^ Republic of Malawi, Ministry of Health: National Sexual and Reproductive Health and Rights (SRHR) Policy. Lilongwe: Ministry of Health. 2016.

^96^ Fitch L, Rodrigues J, Warren M: How policy affects practice: policy barriers to provision of HIV biomedical prevention services in Sub-Saharan Africa. 2020. Reference Source

^97^ The WHO defines the private sector as “those individuals and organizations providing health services or products that are not owned or directly controlled by government.” These include pharmacies, private practitioners and NGOs. WHO: Technical Series on Primary Care: The private sector, universal health coverage and primary health care. Geneva: WHO. 2018. Reference Source

^98^ Kenya National Bureau of Statistics: Kenya Demographic and Health Survey 2014. 2015. Reference Source

^99^ National Statistical Office (NSO) [Malawi] and ICF: Malawi Demographic and Health Survey 2015-16. Zomba, Malawi and Rockville, Maryland, USA: NSO and ICF. 2017. Reference Source

^100^ Zimbabwe National Statistics Agency and ICF International: Zimbabwe Demographic and Health Survey 2015: Final Report. Rockville, Maryland: Zimbabwe National Statistics Agency (ZIMSTAT) and ICF International. 2016. Reference Source

^101^ In Malawi, e.g., the Christian Health Association of Malawi (CHAM), Banja La Mtsogolo (BLM) (MSI affiliate), Family Planning Association of Malawi (FPAM) (IPPF affiliate) and Population Services International (PSI). In Zimbabwe, e.g., Population Services Zimbabwe (MSI affiliate), Pangaea Zimbabwe AIDS Trust (PZAT) and PSI.

^102^ Interview with implementing partner, Malawi, April 2020.

^103^ Government of Zimbabwe, Ministry of Health and Child Care: Strategic Framework for Public-Private Partnerships for TB and HIV Prevention, Treatment, Care and Support.

^104^ Zimbabwe National Statistics Agency and ICF International: Zimbabwe Demographic and Health Survey 2015: Final Report. Rockville, Maryland: Zimbabwe National Statistics Agency (ZIMSTAT) and ICF International. 2016. Reference Source

^105^ University of Washington. Pharmacy Delivery to Expand the Reach of PrEP in Kenya. In: ClinicalTrials.gov [cited 2021 May 25], Available from https://clinicaltrials.gov/ct2/show/NCT04558554.

^106^ Civil society dialogues with AGYW and civil society organizations, Zimbabwe, Nov. 2019.

^107^ Civil society dialogues with AGYW, Kenya, Nov. 2019.

^108^ Civil society dialogues with AGYW, Kenya, Nov. 2019.

^109^ Civil society dialogues with AGYW, Zimbabwe, Nov. 2019.

^110^ Facility assessment data analysis, Kenya, Nov. 2019.

^111^ Facility assessment data analysis, Zimbabwe, March-June 2020.

^112^ Facility assessment data analysis, Kenya, Nov. 2019.

^113^ Interview with healthcare providers and implementing partners, Kenya, Nov. 2019.

^114^ Facility assessment data analysis, Zimbabwe, March-June 2020.

^116^ Government of Zimbabwe, Ministry of Health and Child Care: National Adolescent and Youth Sexual and Reproductive Health (ASRH) Strategy II: 2016-2020. Harare: Ministry of Health and Child Care. 2016.

^117^ Republic of Malawi, Ministry of Health: National Youth Friendly Health Services Strategy, 2015-2020. Lilongwe: Ministry of Health. 2015.

^118^ Facility assessment data analysis, Zimbabwe, March-June 2020.

^119^ Facility assessment data analysis, Kenya, Nov. 2019.

^120^ Interview with healthcare providers, Zimbabwe, March 2020.

^121^ Government of Malawi: National Strategy for Adolescent Girls and Young Women 2018-2022. Lilongwe: Government of Malawi. 2018.

^122^ Facility assessment data analysis, Zimbabwe, March-June 2020.

^125^ PEPFAR: Zimbabwe Country Operational Plan (COP) 2020: Strategic Direction Summary. 2020. Reference Source

^126^ Brittain AW, Loyola Briceno AC, Pazol K, *et al.*: Youth-Friendly Family Planning Services for Young People: A Systematic Review Update. *Am J Prev Med.* 2018; **55**(5): 725–735. PubMed Abstract | Publisher Full Text

^127^ Lee L, Yehia BR, Gaur AH, *et al*.: The Impact of Youth-Friendly Structures of Care on Retention Among HIV-Infected Youth. *AIDS Patient Care STDS.* 2016; **30**(4): 170–7. PubMed Abstract | Publisher Full Text

^128^ Rosenberg NE, Bhushan NL, Vansia D, *et al.*: Comparing Youth-Friendly Health Services to the Standard of Care Through “Girl Power-Malawi”: A Quasi-Experimental Cohort Study. *J Acquir Immune Defic Syndr.* 2018; **79**(4): 458–466. PubMed Abstract | Publisher Full Text

^129^ Kidman R, Waidler J, Palermo T: Uptake of HIV testing among adolescents and associated adolescent-friendly services. *BMC Health Serv Res.* 2020; **20**(1): 881. PubMed Abstract | Publisher Full Text

^130^ Velloza J, Kapogiannis B, Bekker LG, *et al*.: Interventions to improve daily medication use among adolescents and young adults: what can we learn for youth pre-exposure prophylaxis services? *AIDS.* 2021; **35**(3): 463–475. PubMed Abstract | Publisher Full Text

^131^ Callahan T, Modi S, Swanson J, *et al*.: Pregnant adolescents living with HIV: what we know, what we need to know, where we need to go. *J Int AIDS Soc.* 2017; **20**(1): 21858. PubMed Abstract | Publisher Full Text

^132^ UNAIDS: Progress towards the Start Free, Stay Free, AIDS Free targets: 2020 report. Geneva: UNAIDS. 2020. Reference Source

^133^ WHO: Consolidated Guidelines on the Use of Antiretroviral Drugs for Treating and Preventing HIV Infection: Recommendations for a Public Health Approach. WHO: Geneva. 2016. Reference Source

^134^ UNAIDS: AIDSinfo. 2020. Accessed May 14, 2021. Reference Source

^135^ UNAIDS: AIDSinfo. 2020. Accessed May 14, 2021. Reference Source

^136^ PEPFAR: Malawi Country Operational Plan 2020: Strategic Direction Summary. 2020. Reference Source

^137^ Callahan T, Modi S, Swanson J, *et al*.: Pregnant adolescents living with HIV: what we know, what we need to know, where we need to go. *J Int AIDS Soc.* 2017; **20**(1): 21858. PubMed Abstract | Publisher Full Text

^139^ Carbone NB, Njala J, Jackson DJ, *et al.*: “I would love if there was a young woman to encourage us, to ease our anxiety which we would have if we were alone”: Adapting the Mothers2Mothers Mentor Mother Model for adolescent mothers living with HIV in Malawi. *PLoS One.* 2019; **14**(6): e0217693. PubMed Abstract | Publisher Full Text

^140^ Ministry of Health, NASCOP: Kenya Framework for Elimination of Mother-To-Child Transmission of HIV and Syphilis 2016-2021. Nairobi: NASCOP. 2016.

^142^ Government of Zimbabwe, Ministry of Health and Child Care: Plan for Elimination of Mother to Child Transmission of HIV & Syphilis in Zimbabwe 2018-2022. Harare: Ministry of Health and Child Care. 2018. Reference Source

^143^ Facility assessment data analysis, Kenya, Nov. 2019.

^144^ Mugwanya KK, Pintye J, Kinuthia J, *et al.*: Integrating preexposure prophylaxis delivery in routine family planning clinics: A feasibility programmatic evaluation in Kenya. *PLOS Medicine.* 2019; **16**(9): e1002885. PubMed Abstract | Publisher Full Text

^145^ Kinuthia J, Pintye J, Abuna F, *et al.*: Pre-exposure prophylaxis uptake and early continuation among pregnant and post-partum women within maternal and child health clinics in Kenya: results from an implementation programme. *Lancet HIV.* 2020; **7**(1): E38-E48. PubMed Abstract | Publisher Full Text

^146^ Gombe MM, Cakouros BE, Ncube G, *et al.*: Key barriers and enablers associated with uptake and continuation of oral pre-exposure prophylaxis (PrEP) in the public sector in Zimbabwe: Qualitative perspectives of general population clients at high risk for HIV. *PLoS One.* 2020; **15**(1): e0227632. PubMed Abstract | Publisher Full Text

^147^ Interview with MOH officials, healthcare providers and implementing partners, Zimbabwe, March-June 2020.

^148^ Interview with implementing partners, Malawi, April 2020.

^149^ Government of Zimbabwe, Ministry of Health and Child Care: Comprehensive National HIV Communications Strategy for Zimbabwe: 2019-2025. Harare: Ministry of Health and Child Care. 2019. Reference Source

^150^ Ministry of Health, NASCOP: National HIV & STI Combination Prevention Communication Strategy, 2019-2024. 2019.

Interview with implementing partner, March 2020.

^152^ Interview with implementing partners, Malawi, March-April 2020, and MOH officials, Malawi, July 2020.

^153^ Civil society dialogues with AGYW, Kenya, Nov. 2019.

^154^ Civil society dialogues with AGYW and civil society organizations, Zimbabwe, Nov. 2019.

^155^ Interview with local researcher, Malawi, April 2020.

^156^ Interview with implementing partners, Malawi, March-April 2020.

^157^ USAID: DREAMS: Partnership to Reduce HIV/AIDS in Adolescent Girls and Young Women. 2021. Accessed May 25, 2021. Reference Source

^158^ PEPFAR: Country Operational Plan (COP/ROP) 2020 [Kenya]: Strategic Direction Summary. 2020. Reference Source

^159^ PEPFAR: Malawi Country Operational Plan 2020: Strategic Direction Summary. 2020. Reference Source

^160^ Global PrEP Learning Network: PrEP for Adolescent Girls and Young Women (AGYW): Experiences From DREAMS Programs. 2020. Reference Source

^161^ UNAIDS: AIDSinfo. 2020. Accessed May 14, 2021. Reference Source

^162^ HIV Prevention Market Manager: What we know and don’t know about adolescent girls and young women and HIV prevention in sub-Saharan Africa: Mapping findings across completed, ongoing and planned projects. 2018. Reference Source

^163^ Rademacher KH, Solomon M, Brett T, *et al*.: Expanding Access to a New, More Affordable Levonorgestrel Intrauterine System in Kenya: Service Delivery Costs Compared With Other Contraceptive Methods and Perspectives of Key Opinion Leaders. *Glob Health Sci Pract.* 2016; **4**(S2): S83-93. PubMed Abstract | Publisher Full Text

^164^ OPTIONS Consortium: Global PrEP Learning Network: PrEP Integration: Including PrEP with Family Planning Services. 2019. Reference Source

^165^ Government of Zimbabwe, Ministry of Health and Child Care: Comprehensive National HIV Communications Strategy for Zimbabwe: 2019-2025. Harare: Ministry of Health and Child Care. 2019. Reference Source

^166^ Ministry of Health, NASCOP: National HIV & STI Combination Prevention Communication Strategy, 2019-2024. 2019.

^167^ UNAIDS: Unite with Women, Unite against Violence and HIV. Geneva: UNAIDS. 2014. Reference Source

^168^ Ministry of Labour and Social Protection, State Department for Social Protection, Department of Children’s Services: National Prevention and Response Plan on Violence against Children in Kenya 2019-2023. Nairobi: Government of Kenya. 2020. Reference Source

^169^ Together for Girls, Ministry of Labour and Social Protection [Kenya]: Violence Against Children Survey Report 2019 fact sheet. 2020. Reference Source

^170^ Together for Girls, Government of Zimbabwe: Violence Against Children Survey Report 2019 fact sheet. 2019. Reference Source

^171^ Ministry of Gender, Children, Disability and Social Welfare, Republic of Malawi: Executive Summary: Violence Against Children and Young Women in Malawi: Findings from a National Survey 2013. 2014. Reference Source

^172^ Mulambia Y, Miller AJ, MacDonald G, *et al*.: Are one-stop centres an appropriate model to deliver services to sexually abused children in urban Malawi?, *BMC Pediatr.* 2018; **18**: 145. PubMed Abstract | Publisher Full Text

^173^ UNFPA Zimbabwe: UNFPA releases over $2,5 million to support access to scale up provision of Gender Based Violence & sexual reproductive health services during the COVID19 pandemic. 2020. Accessed May 25, 2021. Reference Source

^174^ PEPFAR: PEPFAR 2021 Annual Report to Congress. Washington, DC: PEPFAR. 2021. Reference Source

^176^ The Global Fund: Women & Girls. Accessed May 25, 2021. Reference Source

^177^ U.S. Department of State: DREAMS Partnership. Accessed May 25, 2021. Reference Source

^178^ PEPFAR: PEPFAR 2021 Annual Report to Congress. Washington, DC: PEPFAR. 2021. Reference Source

^179^ The Global Fund: The Global Fund Data Explorer. Accessed May 25, 2021. Reference Source

^180^ PEPFAR: Malawi: DREAMS Overview (FY 2016-2021). 2020. Reference Source

^181^ Georgetown University Center for Innovation in Global Health: Opportunities and Challenges for the Integration of HIV and SRH Services in Malawi. 2020. Unpublished report.

^182^ Mazur A, Brindis CD, Decker MJ: Assessing youth-friendly sexual and reproductive health services: a systematic review. *BMC Health Serv Res.* 2018; *18*(1): 216. PubMed Abstract | Publisher Full Text

^183^ Civil society dialogues with AGYW, Kenya, Nov. 2019.

^186^ Denno DM, Hoopes AJ, Chandra-Mouli V: Effective Strategies to Provide Adolescent Sexual and Reproductive Health Services and to Increase Demand and Community Support. *J Adolesc Health.* 2015; **56**(1): S22-S41. PubMed Abstract | Publisher Full Text

^187^ HIV Prevention Market Manager, Ministry of Health [Kenya]: Integration of HIV prevention and sexual and reproductive health services in Kenya. 2020. Reference Source

^193^ Siapka M, Obure CD, Mayhew SH, *et al.*: Impact of integration of sexual and reproductive health services on consultation duration times: results from the Integra Initiative, *Health Policy Plan.* 2017; **32**(S4): iv82-iv90. PubMed Abstract | Publisher Full Text

^194^ Seidman G, Atun R: Does task shifting yield cost savings and improve efficiency for health systems? A systematic review of evidence from low-income and middle-income countries. *Hum Resour Health.* 2017; **15**: 29. PubMed Abstract | Publisher Full Text

^195^ Ministry of Health [Malawi], Reproductive Health Department, USAID, Evidence to Action Project, Centre for Social Research, University of Malawi: Evaluation of Youth-Friendly Health Services in Malawi. 2014. Reference Source

^196^ Smit JA, Church K, Milford C, *et al.*: Key informant perspectives on policy- and service-level challenges and opportunities for delivering integrated sexual and reproductive health and HIV care in South Africa, *BMC Health Serv Res.* 2012; **12**: 48. PubMed Abstract | Publisher Full Text

^197^ Mayhew SH, Sweeney S, Warren CE, *et al.*: Numbers, systems, people: how interactions influence integration. Insights from case studies of HIV and reproductive health services delivery in Kenya, *Health Policy Plan.* 2017; **32**(S4): iv67-iv81. PubMed Abstract | Publisher Full Text

^198^ Ndwiga C, Abuya T, Mutemwa R, *et al.*: Exploring experiences in peer mentoring as a strategy for capacity building in sexual reproductive health and HIV service integration in Kenya, *BMC Health Serv Res.* 2014; **14**: 98. PubMed Abstract | Publisher Full Text

^199^ Mayhew SH, Sweeney S, Warren CE, *et al.*: Numbers, systems, people: how interactions influence integration. Insights from case studies of HIV and reproductive health services delivery in Kenya, *Health Policy Plan.* 2017; **32**(S4): iv67-iv81. PubMed Abstract | Publisher Full Text

^200^ Bigogo G, Audi A, Aura B, *et al.*: Health-seeking patterns among participants of population-based morbidity surveillance in rural western Kenya: implications for calculating disease rates. *Int J Infect Dis.* 2010; **14**(11): e967–e973. PubMed Abstract | Publisher Full Text

^204^ Ladner J, Davis B, Audureau E, *et al*.: Treatment-seeking patterns for malaria in pharmacies in five sub-Saharan African countries. *Malar J.* 2017; **16**(1): 353. PubMed Abstract | Publisher Full Text

^205^ Mugo PM, Prins HAB, Wahome EW, *et al*.: Engaging young adult clients of community pharmacies for HIV screening in coastal Kenya: a cross-sectional study. *Sex Transm Infect.* 2015; **91**(4): 257–259. PubMed Abstract | Publisher Full Text

^206^ Tung EL, Thomas A, Eichner A, *et al*.: Implementation of a community pharmacy-based pre-exposure prophylaxis service: a novel model for pre-exposure prophylaxis care. *Sex Health.* 2018; **15**(6): 556–561. PubMed Abstract | Publisher Full Text

^207^ Ortblad KF, Mogere P, Roche S, *et al.*: Design of a care pathway for pharmacy-based PrEP delivery in Kenya: results from a collaborative stakeholder consultation. *BMC Health Serv Res*. 2020; **20**(1): 1034. PubMed Abstract | Publisher Full Text

^208^ Mugwanya KK, Pintye J, Kinuthia J, *et al.*: Integrating preexposure prophylaxis delivery in routine family planning clinics: A feasibility programmatic evaluation in Kenya. *PLOS Medicine.* 2019; **16**(9): e1002885. PubMed Abstract | Publisher Full Text

^209^ Rousseau E, Baeten JM, Morton J, *et al.*: Association between contraceptive use and PrEP uptake and continuation in adolescent girls and young women in Cape Town, South Africa. 2019. Reference Source

^210^ Civil society dialogues with AGYW, Kenya, Nov. 2019.

^211^ Civil society dialogues with AGYW and civil society organizations, Zimbabwe, Nov. 2019.

