# Supplementary material for: Integration of HIV prevention and sexual and reproductive health in the era of anti-retroviral-based prevention: findings from assessments in Kenya, Malawi and Zimbabwe
Source: Gates Open Res. 2022 Mar 28;5:145. Originally published 2021 Sep 15. [Version 2] doi: 10.12688/gatesopenres.13330.2 (PMC10547113; doi:10.12688/gatesopenres.13330.2)
Supplement: Peer review history

# Reviewer report: Reviewer response for version 2

**DOI:** 10.21956/gatesopenres.14876.r34555

**Published:** 2023-09-07

**Authors:** Cheryl Baxter

**Affiliations:** 1 Stellenbosch University, Stellenbosch, Western Cape, South Africa

The authors describe the progress made by Kenya, Malawi, and Zimbabwe in integrating HIV prevention and sexual and reproductive health services. The focus is primarily on the integration of oral PrEP and family planning (FP) for adolescent girls and young women (AGYW). The manuscript highlights the high HIV prevalence among young women in these countries and the need to reach this population with comprehensive HIV prevention and SRH services. It also discusses the current trends and landscape of HIV prevention and SRH in each country. The manuscript identifies opportunities for improved integration. The authors recommend increased government and donor investment and political support to scale up HIV prevention/SRH integration in high HIV-burden countries.

 This is a timely article and draws attention to an important topic that will need to be addressed if we hope to address the high rates of HIV and pregnancy in AGYW.

**Major comments:**

 While the methods section briefly mentions that no ethics approval was sought for this article because it analyzes aggregate findings from completed rapid assessments, it's important to provide a more thorough discussion of ethical considerations. Please explain how confidentiality and privacy were ensured during the data collection process. This clarification will enhance the transparency and ethical rigor of the study.

**Minor comments:**

Introduction:

- A citation should be included for the sentence "The SARS-CoV-2 (COVID-19) pandemic has amplified ... low- and middle-income settings. " 

Methods:

- Can you include the actual number of AGYW and NGOs included in the community dialogues?
- In the data analysis for rapid assessment section, the authors mention that the research team "analysed HIV and SRH information and services" for the Malawi data but do not elaborate on how the analysis was actually done. Were the interviews analysed in the same way as the interviews conducted in Kenya and Zimbabwe?
- Also, the sentence "The analysis highlighted social and structural barriers ... presented by the COVID-19 pandemic." is a result and should be moved to the results section.

Results:

- The authors state that "Integration pilots are planned in 2021 in five counties.". If these were implemented in 2021 then the sentence should be changed to state that these were implemented. 
- Define CBDAs when used for the first time. 
- RMNCAH should also be defined.

Is the work clearly and accurately presented and does it cite the current literature?

Yes

If applicable, is the statistical analysis and its interpretation appropriate?

Not applicable

Are all the source data underlying the results available to ensure full reproducibility?

Yes

Is the study design appropriate and is the work technically sound?

Yes

Are the conclusions drawn adequately supported by the results?

Yes

Are sufficient details of methods and analysis provided to allow replication by others?

Partly

Reviewer Expertise:

HIV prevention

I confirm that I have read this submission and believe that I have an appropriate level of expertise to confirm that it is of an acceptable scientific standard.

# Reviewer report: Reviewer response for version 2

**DOI:** 10.21956/gatesopenres.14876.r31900

**Published:** 2022-04-12

**Authors:** Morkor Newman Owiredu

**Affiliations:** 1 Global HIV, Hepatitis and STI programme, World Health Organization, Geneva, Switzerland

I am quite happy with the revisions, however I still find it a little difficult to agree with the conclusion. 

 Kindly see the following:

 "While the call for HIV/SRH integration is not new, it has been **implemented more successfully for HIV treatment and SRH**, while integration of HIV prevention with SRH has lagged."

 I think this is too broad and does not ring true for the whole of HIV SRH integration. HIV treatment and SRH integration is not necessarily ahead of HIV prevention and SRH where many are receiving HIV testing services within SRH.

 Example:

- FP, which is part of SRH, is poorly implemented in HIV treatment whereas HIV prevention and FP seem well linked. 
- Both FP and HIV treatment are subtopics in the wider scope of HIV and SRH.

I have not been able to accept the statement that compares implementation because the two topics (HIV and SRH) are too broad and linkages discussed are only within sub topics. 

 As with the previous title, there may be a need to better define the conclusion. 

 Thus the status remains ‘approved with reservations’ with a request for the authors to be more specific with the conclusions.

Is the work clearly and accurately presented and does it cite the current literature?

Yes

If applicable, is the statistical analysis and its interpretation appropriate?

Not applicable

Are all the source data underlying the results available to ensure full reproducibility?

Partly

Is the study design appropriate and is the work technically sound?

Yes

Are the conclusions drawn adequately supported by the results?

Yes

Are sufficient details of methods and analysis provided to allow replication by others?

Yes

Reviewer Expertise:

NA

I confirm that I have read this submission and believe that I have an appropriate level of expertise to confirm that it is of an acceptable scientific standard, however I have significant reservations, as outlined above.

# Reviewer report: Reviewer response for version 1

**DOI:** 10.21956/gatesopenres.14573.r31182

**Published:** 2021-11-29

**Authors:** Rose Patricia Oluoch

**Affiliations:** 1 USAID, Nairobi, Kenya

It is my pleasure to provide a review for this article and thank you for considering and giving me the opportunity to do so.

 The article aims to analyse the progress made by the three study countries; Kenya, Malawi and Zimbabwe, towards the integration of HIV prevention and Sexual Reproductive Health (SRH). It provides challenges and opportunities to deliver comprehensive integrated HIV/SRH services in the region.

 It is a timely article especially as countries now gear towards the HIV epidemic control and are striving for HIV program sustainability approaches. It is rich in content with relevant information and supporting citations.

 I provide below some points that the authors and readers alike may need to consider in order to gain maximally from the analysis and thoughts shared.

**1. Introduction:**

 a) HIV prevention and SRH trends and landscape section:

- Although PrEP and FP have traditionally been mainly accessed from public facilities in the past, program implementers have devised innovative approaches to mitigate the challenges posed by the COVID pandemic.
- Readers would benefit if the authors provided the citation or actual data on HIV prevalence and incidence and quantified "the large percentage of AGYW who gave birth before age 18".

**2. Methods:**

- The researchers should consider providing more details on the number of sites and settings by category and how these were selected in order to inform generalization. Additionally, explain the specific roles of the ministry of health personnel and the HIV prevention market manager project staff. The article says the analysis was completed by Ministry of Health and PMM. It is not clear if this was the actual data collection or data analysis. 
- Provide more information on how WhatsApp is used for interviews in Malawi.
- It may be helpful if there is a clear distinction between data collection and analysis, they seem to have been used interchangeably.
- The sentence "The research team standardised interview protocols and analysis of data" is not very clear.
- Explain what is meant by use of standard informed consent procedures that were followed.
- A clear explanation on how the qualitative data analysis was done would be helpful, was there a codebook? How was it developed? Who did the coding? How were coding inconsistencies resolved? 

**3. Results:**

- While there was an attempt or intention to group results according to distinct sub-themes, namely Health systems and Service delivery, it is not easy to decipher what the actual findings were because the section also contains largely what should fit in the discussion. It would be helpful if the results were stated as were found without interpretation or justification. See the second paragraph under the subsection **Enabling environment and policy implementation** as a perfect example of this observation. 
- The article states that there are operational challenges of reaching AGYW with YFHS and safe spaces but does not state what the challenges are, leaving the reader with more questions.
- The third paragraph is not entirely true that prior to the assessment Kenya had separate mechanisms supporting the rollout of SRH and HIV services as there exists the  National Reproductive Health and HIV and AIDS Integration Strategy of 2009. 

**4. Discussion:**

- Some of the elements that should come out in the discussion were interspersed in Results as stated above.
- Operationalization of integration may be the elephant in the room. For example in Kenya the supermarket strategy was adopted in the 1980s. The researchers should consider exploring and providing practical recommendations on how to operationalise integration, for example dealing with infrastructure challenges, effective client flow re-organization to achieve integration and most importantly managerial support coupled with attitude change among providers, not forgetting effective coordination and good will at the highest level cascaded down to the low level facilities. 

**5. Conclusion:**

- Reference made to PrEP is not clear as the conclusion is on HIV prevention, PrEP included. It may cause confusion to readers who may equate HIV prevention to PrEP only.

Is the work clearly and accurately presented and does it cite the current literature?

Partly

If applicable, is the statistical analysis and its interpretation appropriate?

Not applicable

Are all the source data underlying the results available to ensure full reproducibility?

No

Is the study design appropriate and is the work technically sound?

Yes

Are the conclusions drawn adequately supported by the results?

Partly

Are sufficient details of methods and analysis provided to allow replication by others?

No

Reviewer Expertise:

HIV Epidemiology,  HTS, Violence against Children, Gender based violence ( GBV)  and PrEP implementation and scale up

I confirm that I have read this submission and believe that I have an appropriate level of expertise to confirm that it is of an acceptable scientific standard, however I have significant reservations, as outlined above.

# Response

**Published:** 2022-03-18

**Authors:** Kate Segal

**Affiliations:** AVAC, USA

It is my pleasure to provide a review for this article and thank you for considering and giving me the opportunity to do so.

 The article aims to analyse the progress made by the three study countries; Kenya, Malawi and Zimbabwe, towards the integration of HIV prevention and Sexual Reproductive Health (SRH). It provides challenges and opportunities to deliver comprehensive integrated HIV/SRH services in the region.

 It is a timely article especially as countries now gear towards the HIV epidemic control and are striving for HIV program sustainability approaches. It is rich in content with relevant information and supporting citations.

 I provide below some points that the authors and readers alike may need to consider in order to gain maximally from the analysis and thoughts shared.

- ***Author response: **Thank you very much. We appreciate your feedback.*

1. Introduction:

 a) HIV prevention and SRH trends and landscape section:

 Although PrEP and FP have traditionally been mainly accessed from public facilities in the past, program implementers have devised innovative approaches to mitigate the challenges posed by the COVID pandemic.

- ***Author response: **The reviewer’s comment is well-taken. We have added a sentence to address this point: *" *Notably, the COVID-19 pandemic has expanded innovations in service delivery, broadening access to oral PrEP and FP through decentralized, differentiated and private sector channels in an effort to mitigate disruptions to accessing services at public health facilities."*

Readers would benefit if the authors provided the citation or actual data on HIV prevalence and incidence and quantified "the large percentage of AGYW who gave birth before age 18".

- ***Author response: **As suggested by the reviewer, we have added the data in-text and corresponding citations. The data are also included in Figure 1. We have changed the sentence to:* *"High HIV prevalence and incidence among AGYW in Kenya (2.1% and 1.9%), Malawi (3.7% and 3.1%) and Zimbabwe (5.2% and 4.2%), and the large percentage of AGYW in these countries who gave birth before age 18 (23.3%, 34.7% and 22%, respectively), signal the need to better reach this population with HIV prevention and SRH information and services."*

2. Methods:

 The researchers should consider providing more details on the number of sites and settings by category and how these were selected in order to inform generalization. Additionally, explain the specific roles of the ministry of health personnel and the HIV prevention market manager project staff. The article says the analysis was completed by Ministry of Health and PMM. It is not clear if this was the actual data collection or data analysis. 

- ***Author response: **In response to the reviewer’s comment, we have added a new chart (Figure 2) that displays the sites where the facility assessments were conducted in Kenya and Zimbabwe as well as an explanation of site selection criteria:​​* *"In Kenya and Zimbabwe, research teams identified a cross-section of ten health facilities in each country, primarily in the public sector, across high and low volume sites in both urban and rural settings, including youth-friendly (YF) sites."*

- *To respond to the reviewer’s second point, we specified that the HIV Prevention Market Manager project was “represented by AVAC” and added a sentence to clarify the respective roles of MOH representatives and AVAC:* *"MOH representatives identified sites, key informants and led data collection at country level. AVAC led development of data collection tools, analysis of findings and participated in data collection in Kenya."*

Provide more information on how WhatsApp is used for interviews in Malawi. 

- ***Author response: **We have elaborated on the rationale and process for conducting interviews via WhatsApp in Malawi. We have changed the sentence to: "Given travel and social distancing restrictions due to COVID-19, in-person interviews were substituted with interviews through WhatsApp calls to key informants’ cell phones (which are used widely in Malawi), video calls (Zoom or Teams), telephone and email."*

It may be helpful if there is a clear distinction between data collection and analysis, they seem to have been used interchangeably.

- ***Author response: **As suggested by the reviewer, we have separated the data collection and analysis sections for the country assessments, which are now titled: “Data collection for rapid assessments” and “Data analysis for rapid assessments,” respectively. We have re-organized the information within these sections to ensure a cohesive flow.*

The sentence "The research team standardised interview protocols and analysis of data" is not very clear.

- ***Author response: **We agree with the reviewer’s comment and have replaced the sentence with a description of the process that all research teams followed for conducting interviews, as follows: *

*"Interviews began with an introduction of the researchers and explanation of the research purpose and focus of discussion. Oral informed consent was sought to participate in the interview as well as to audio-record the conversation, where feasible, and confidentiality protocols were explained. Informed consent was then obtained by research teams in each country. Interviews were recorded and notes taken simultaneously, and facility assessment data from Kenya and Zimbabwe were logged in a spreadsheet. Interview transcripts, notes, recordings and health facility assessments were only accessible by the research teams.*

*PMM used standardized interview guides and health facility assessment checklists to conduct the rapid assessment and analysis of data across Kenya and Zimbabwe. The research teams compiled detailed notes after each interview using a standard template, referencing recorded interviews to ensure accuracy and attention to detail, and to document supporting quotes. Health facility assessments collected quantitative data on HIV, FP/SRH and YF services available (e.g., methods and hours offered, whether and how services are integrated, commodity stock-outs, YF accommodations) and staffing and general infrastructure (e.g., facility size, number and cadres of staff across HIV, SRH and YF service areas) to analyze the level of HIV prevention/SRH integration and YF service delivery at each facility. To understand opportunities for and barriers to HIV prevention/SRH integration, each team conducted thematic analysis of detailed notes through a workshop once data collection was completed to identify common themes and outlier perspectives across the interviews, grouped by stakeholder perspectives at the government, health facility and community levels. Findings were triangulated across qualitative and quantitative data."*

- *In addition, we feel that separating out and adding further detail to the section Data analysis for rapid assessments, per the reviewer’s recommendation above, has clarified how analysis of the data for the rapid assessments was conducted​​.*

Explain what is meant by use of standard informed consent procedures that were followed.

- ***Author response:*** *As suggested by the reviewer, we have built out an explanation of the process for obtaining informed consent, as follows:*

*"Interviews began with an introduction of the researchers and explanation of the research purpose and focus of discussion. Oral informed consent was sought to participate in the interview as well as to audio-record the conversation, where feasible, and confidentiality protocols were explained. Informed consent was then obtained by research teams in each country. Interviews were recorded and notes taken simultaneously, and facility assessment data from Kenya and Zimbabwe were logged in a spreadsheet. Interview transcripts, notes, recordings and health facility assessments were only accessible by the research teams."*

 A clear explanation on how the qualitative data analysis was done would be helpful, was there a codebook? How was it developed? Who did the coding? How were coding inconsistencies resolved? 

- ***Author response: **We have expanded upon the data analysis methodology for the rapid country assessments, as follows:*

*"PMM used standardized interview guides and health facility assessment checklists to conduct the rapid assessment and analysis of data across Kenya and Zimbabwe. The research teams compiled detailed notes after each interview using a standard template, referencing recorded interviews to ensure accuracy and attention to detail, and to document supporting quotes. Health facility assessments collected quantitative data on HIV, FP/SRH and YF services available (e.g., methods and hours offered, whether and how services are integrated, commodity stock-outs, YF accommodations) and staffing and general infrastructure (e.g., facility size, number and cadres of staff across HIV, SRH and YF service areas) to analyze the level of HIV prevention/SRH integration and YF service delivery at each facility. To understand opportunities for and barriers to HIV prevention/SRH integration, each team conducted thematic analysis of detailed notes through a workshop once data collection was completed to identify common themes and outlier perspectives across the interviews, grouped by stakeholder perspectives at the government, health facility and community levels. Findings were triangulated across qualitative and quantitative data.*

*To understand the spectrum of services in the Blantyre district, the research team analyzed HIV and SRH information and services offered through public sector facilities and youth-friendly health services (YFHS), as well as in the private sector, including national and international NGOs and NGOs with the U.S.-led DREAMS initiative. The analysis highlighted social and structural barriers for sustained and effective use of prevention interventions, including through the education system and gender-based violence (GBV) programs, and new and evolving challenges presented by the COVID-19 pandemic."*

- *In addition, we added a reference to an established framework for conducting rapid assessments: " *These rapid assessments utilized proven methodologies for rapid assessments, building from WHO and Measure Evaluation’s “Routine Health Information System Rapid Assessment Tool.”"**

3. Results:

 While there was an attempt or intention to group results according to distinct sub-themes, namely Health systems and Service delivery, it is not easy to decipher what the actual findings were because the section also contains largely what should fit in the discussion. It would be helpful if the results were stated as were found without interpretation or justification. See the second paragraph under the subsection Enabling environment and policy implementation as a perfect example of this observation. 

- ***Author response: **We agree with the reviewer’s assessment. We have made line edits throughout the Results section to clarify where it may have been unclear that certain statements were findings (including in the second paragraph of the section Enabling environment and policy implementation). In addition, we made larger, structural edits to the manuscript that streamlined the Results to only include findings from this analysis and moved sentences that are interpretations or recommendations to the Discussion section.  *

The article states that there are operational challenges of reaching AGYW with YFHS and safe spaces but does not state what the challenges are, leaving the reader with more questions.

- ***Author response: **We have revised this sentence to note specific operational challenges to reaching AGYW with YFHS and safe spaces, as follows: "Operationalizing policies and translating them to local/district levels continues to present challenges, especially in reaching AGYW with YFHS and safe spaces, due in large part to public-sector resource constraints that entrench HIV and SRH silos, rely on referral systems and limit provider training to offer integrated oral PrEP/FP services and deliver them in a non-judgmental, confidential and affordable way."*

The third paragraph is not entirely true that prior to the assessment Kenya had separate mechanisms supporting the rollout of SRH and HIV services as there exists the National Reproductive Health and HIV and AIDS Integration Strategy of 2009. 

- ***Author response: **Thank you for underscoring this. We added a sentence to distinguish that while policies on HIV prevention and SRH integration were in place prior to the assessment in Kenya, operationalization of integration remained an issue: "Although policies and strategies on HIV prevention/SRH existed in Kenya, services were not consistently integrated in practice. Prior to the rapid assessment, the government largely had separate programs supporting the operational rollout of SRH and HIV prevention services at national and sub-national levels."*

4. Discussion:

 Some of the elements that should come out in the discussion were interspersed in Results as stated above.

- ***Author response: **In accordance with the reviewer’s suggestion and as noted in our response above, we have moved portions of the Results to the Discussion (and vice versa where appropriate) to strengthen the flow and logic of the manuscript.*

Operationalization of integration may be the elephant in the room. For example in Kenya the supermarket strategy was adopted in the 1980s. The researchers should consider exploring and providing practical recommendations on how to operationalise integration, for example dealing with infrastructure challenges, effective client flow re-organization to achieve integration and most importantly managerial support coupled with attitude change among providers, not forgetting effective coordination and good will at the highest level cascaded down to the low level facilities. 

- ***Author response: **Per the reviewer’s recommendation, we have expanded upon practical ways to operationalize integration in the Discussion section in the following paragraphs. Some of these points were moved from the Results section, as they were more akin to recommendations, while others were newly added to sufficiently address the comment.*

*"Dedicated structures are required to support implementation of integration throughout the health system. Updating outdated national integration policies to include PrEP can mandate programs to deliver PrEP products as part of the package of integrated services. Cascading these policies to sub-national HIV and FP programs and down to facilities – with appropriate resourcing, coordination mechanisms and training – will help to operationalize them. Leadership by sub-national HIV and FP program coordinators is fundamental to drive development and execution of integrated workplans, budgets and meetings and to provide oversight to and monitor implementation at facilities. Kenya’s HIV/SRH integration sub-committee, described above, is a model other countries can emulate to move the needle.*

*Health systems adaptations will also be needed to effectuate integration. Revising and strengthening M&E systems to document the provision of HIV prevention services in SRH service areas and vice versa, and integrating systems up the chain to national level, would enhance the ability to look at data in an integrated fashion. Electronic monitoring systems, currently being rolled out in Kenya, Malawi and Zimbabwe, provide an opportunity to track individual client health records across services and facilities. Data should be made available in real time and disaggregated by age to improve targeting of at-risk AGYW for evidence-based decision-making. Bi-directional integration of data systems can also support the integrated management of PrEP and FP commodities.*

*While health systems changes can take time, there are interim measures that health facilities can adopt to facilitate integration and support access for AGYW. These include: “whole site training,” where administrative and clinical staff alike are sensitized on FP, HIV testing and PrEP services and where to access them; accompanied referral for AGYW to minimize loss to follow-up; fast-tracking young people in queues; displaying information, education and communication (IEC) materials on SRH and HIV prevention in all service areas; extending hours that services are available, particularly to afternoons and weekends, which may be more convenient for young people; allocating staff according to daily client flow and employing health systems navigators and peer educators, who may be preferred messengers for AGYW, to conduct counseling, escort clients to services and follow up on referrals, thereby offloading these tasks from provider workloads. …*

*The need for capacity-building and building empathy among providers is evident to mobilize AGYW to access SRH and HIV prevention services. Providers often recognized the benefits of HIV prevention/SRH integration for clients even if it increased workloads in the initial stages of integration. Strengthening soft skills, such as training on values clarification and person-centered care, must be coupled with clinical training to address negative provider attitudes that often dissuade AGYW from seeking services, particularly in public facilities. Mentorship for providers that includes skills-building on integrated services has increased uptake, range of services offered, provider self-confidence and quality of care in resource-constrained settings. Supportive managerial supervision that empowers providers to make decisions and fosters teamwork has improved delivery of integrated services and helped staff to surmount structural issues, such as stock-outs or workload distribution."*

 5. Conclusion:

 Reference made to PrEP is not clear as the conclusion is on HIV prevention, PrEP included. It may cause confusion to readers who may equate HIV prevention to PrEP only.

- ***Author response: **Per the reviewer’s suggestion, we have specified where we are referring to HIV prevention and SRH and to PrEP/HIV prevention products specifically, as follows:*

*"While the call for HIV/SRH integration is not new, it has been implemented more successfully for HIV treatment and SRH, while integration of HIV prevention with SRH has lagged. Although integrated policies exist on paper, HIV prevention and SRH services remain siloed and these policies preceded the introduction of new biomedical interventions such as oral PrEP. With the introduction of HIV prevention products expected in the next couple of years, there is an urgency to ensure that policies on integration are updated and that programs build on lessons where HIV prevention and SRH integration has been successful. In Kenya, Malawi and Zimbabwe, YFHS and PMTCT programs offer the strongest examples of integration of HIV prevention and SRH that can be leveraged and expanded to more effectively reach AGYW."*

# Reviewer report: Reviewer response for version 1

**DOI:** 10.21956/gatesopenres.14573.r31183

**Published:** 2021-11-09

**Authors:** Daniel K. Were

**Affiliations:** 1 Jhpiego, Nairobi, Kenya

The article is very timely, well written, and presents findings of relevance to the HIV prevention and sexual reproductive health field globally. There is greater recognition that integration is a critical pillar for effective scale up of HIV prevention and sexual reproductive health interventions, and it is also essential in improving both HIV prevention and sexual and reproductive health outcomes. 

 The authors have comprehensively outlined the progress, opportunities and some of the key challenges to integration, including policy, health system, service delivery and demand creation gaps, that warrant redress. The explication of the structural and ecological issues that are determinant of effective integration is commendable. Nonetheless, a substantial barrier to effective integration is provider attitudes. Perceived workload and moral opposition towards AGYW using HIV prevention and SRH interventions are some of the major roadblocks to integration that warrants greater attention for a comprehensive view of health system and service delivery gaps. Further, while the authors acknowledge there are capacity building gaps for providers, of greater need are the soft skills such as communication and counseling skills, which are critical in providing services sensitively to AGYW. 

 The authors attempt to situate the article within the broader HIV prevention and sexual and reproductive health discourse. However, the findings are primarily focused on PrEP and contraception/family planning. Other HIV prevention and SRH interventions e.g. STIs are barely addressed. I would suggest that the authors include a statement to define the scope of the article. In my view, the authors use PrEP and FP to highlight integration issues that are broadly relevant to HIV prevention and sexual reproductive health.  

 My major concern is the description of the methodology and the ethics statement points out that this article was based on secondary data analysis. For instance, the authors point out "...While original data from key informant interviews and health facility assessments are not contained in this article, because the analyses form the basis of this article, their methodologies are elaborated upon below...". Two major issues related to methodology:

1. If the article is based on secondary data analysis, it makes sense for the authors to sufficiently describe the secondary data collection and analysis procedures, compared to the primary data collection and analysis that is given prominence in the article.  
2. The presentation of the findings extensively highlights excerpts from interviews with participants, which is characteristic of primary data, as opposed to secondary data analysis. Wouldn't it make sense to revise the methodology and acknowledge the use of primary data from key informant interviews? 

Is the work clearly and accurately presented and does it cite the current literature?

Yes

If applicable, is the statistical analysis and its interpretation appropriate?

Not applicable

Are all the source data underlying the results available to ensure full reproducibility?

Partly

Is the study design appropriate and is the work technically sound?

Yes

Are the conclusions drawn adequately supported by the results?

Yes

Are sufficient details of methods and analysis provided to allow replication by others?

Yes

Reviewer Expertise:

HIV prevention, oral PrEP

I confirm that I have read this submission and believe that I have an appropriate level of expertise to confirm that it is of an acceptable scientific standard, however I have significant reservations, as outlined above.

# Response

**Published:** 2022-03-18

**Authors:** Kate Segal

**Affiliations:** AVAC, USA

The article is very timely, well written, and presents findings of relevance to the HIV prevention and sexual reproductive health field globally. There is greater recognition that integration is a critical pillar for effective scale up of HIV prevention and sexual reproductive health interventions, and it is also essential in improving both HIV prevention and sexual and reproductive health outcomes. 

- ***Author response:*** *Thank you very much – we agree that integration is crucial to realizing access to and improving the quality of comprehensive HIV prevention and SRH services, and could positively impact health outcomes, particularly for AGYW.*

The authors have comprehensively outlined the progress, opportunities and some of the key challenges to integration, including policy, health system, service delivery and demand creation gaps, that warrant redress. The explication of the structural and ecological issues that are determinant of effective integration is commendable. Nonetheless, a substantial barrier to effective integration is provider attitudes. Perceived workload and moral opposition towards AGYW using HIV prevention and SRH interventions are some of the major roadblocks to integration that warrants greater attention for a comprehensive view of health system and service delivery gaps. Further, while the authors acknowledge there are capacity building gaps for providers, of greater need are the soft skills such as communication and counseling skills, which are critical in providing services sensitively to AGYW. 

- ***Author response: **This comment is very well-taken and important to address in greater detail. We have added a paragraph that speaks to this issue in the section Healthcare provider capacity to offer integrated services to AGYW, as follows:*

*"In addition to a need to strengthen hard skills such as PrEP delivery, this analysis found that negative provider attitudes toward AGYW seeking PrEP and FP services are a key barrier to access in the public sector across countries. In community dialogues in Kenya and Zimbabwe, AGYW described facing stigma from healthcare providers, particularly if they were suspected to be or engaging in sex work or living with HIV, and that privacy and confidentiality are often disregarded, which can demotivate them from returning. In Malawi, the assessment found that sexually-active AGYW, in particular if they are unmarried or have not given birth, are similarly disparaged by providers in public facilities. These findings echo published literature on provider bias toward young people, pointing to a need for training on “soft skills” such as YF communication as a component of provider capacity-building."*

- *In addition, we incorporated the need to build soft skills amongst healthcare providers in the Discussion section, as follows:*

*"The need for capacity-building and building empathy among providers is evident to mobilize AGYW to access SRH and HIV prevention services. Providers often recognized the benefits of HIV prevention/SRH integration for clients even if it increased workloads in the initial stages of integration. Strengthening soft skills, such as training on values clarification and person-centered care, must be coupled with clinical training to address negative provider attitudes that often dissuade AGYW from seeking services, particularly in public facilities. Mentorship for providers that includes skills-building on integrated services has increased uptake, range of services offered, provider self-confidence and quality of care in resource-constrained settings. Supportive managerial supervision that empowers providers to make decisions and fosters teamwork has improved delivery of integrated services and helped staff to surmount structural issues, such as stock-outs or workload distribution."*

 The authors attempt to situate the article within the broader HIV prevention and sexual and reproductive health discourse. However, the findings are primarily focused on PrEP and contraception/family planning. Other HIV prevention and SRH interventions e.g. STIs are barely addressed. I would suggest that the authors include a statement to define the scope of the article. In my view, the authors use PrEP and FP to highlight integration issues that are broadly relevant to HIV prevention and sexual reproductive health.  

- ***Author response: **We agree with the reviewer’s comment. We have made edits throughout the manuscript to specify and differentiate where the findings are specific to PrEP/FP where not clear. In addition, we have added a statement upfront to clarify the scope of the article and the primary focus on oral PrEP and FP, as follows:*

*"This article analyzes the progress that Kenya, Malawi and Zimbabwe have made towards the integration of HIV prevention and SRH (HIV prevention/SRH integration). While the term HIV prevention/SRH integration includes an array of services, this article focuses primarily on the integration of oral PrEP and family planning (FP). Examples from each country highlight promising approaches to integration in the context of HIV prevention, identifying opportunities for improved service delivery and pinpointing persistent gaps that effectively limit the impact of high-potential interventions."*

 My major concern is the description of the methodology and the ethics statement points out that this article was based on secondary data analysis. For instance, the authors point out "...While original data from key informant interviews and health facility assessments are not contained in this article, because the analyses form the basis of this article, their methodologies are elaborated upon below...". Two major issues related to methodology:

 A. If the article is based on secondary data analysis, it makes sense for the authors to sufficiently describe the secondary data collection and analysis procedures, compared to the primary data collection and analysis that is given prominence in the article.  

 B. The presentation of the findings extensively highlights excerpts from interviews with participants, which is characteristic of primary data, as opposed to secondary data analysis. Wouldn't it make sense to revise the methodology and acknowledge the use of primary data from key informant interviews? 

- ***Author response: **Thank you for pointing this out. We have clarified in the introductory paragraph of the Methods section that this article incorporates primary data from key informant interviews, in accordance with point B above, as follows:*

*"This article is a comparative analysis of findings from three completed rapid health systems and landscaping assessments of the integration of HIV prevention, including oral PrEP, in SRH services in Kenya, Malawi and Zimbabwe. These rapid assessments utilized proven methodologies for rapid assessments, building from WHO and Measure Evaluation’s “Routine Health Information System Rapid Assessment Tool.” The assessments collected programmatic and clinic data from public health facilities and NGO-supported programs, and gathered insights from Ministry of Health (MOH) officials, providers, NGO staff and others working at district/county levels, and employed a thematic analysis of data generated, with the intent to rapidly inform national policies and programs. By synthesizing, analyzing and contextualizing the findings of the three assessments, this article aims to understand the current state of HIV prevention/SRH integration in each country and to elucidate cross-country trends. Summaries of and quotes from primary qualitative data from the rapid assessments were included in this article to underscore findings, and outside literature was incorporated to provide additional context. Because the rapid assessments comprise the main source material of this article, their methodologies are elaborated upon below."*

# Reviewer report: Reviewer response for version 1

**DOI:** 10.21956/gatesopenres.14573.r31185

**Published:** 2021-10-01

**Authors:** Morkor Newman Owiredu

**Affiliations:** 1 Global HIV, Hepatitis and STI programme, World Health Organization, Geneva, Switzerland

A very interesting and well-written paper on a topic for which information is needed urgently. The investigations were extensive, especially for the qualitative information gathered.

 However, starting with the title, the initial impression is the integration of HIV response and sexual and reproductive health. The HIV response consists of HIV prevention, treatment, and care, and the paper is focused only on HIV prevention in HIV negatives, PrEP.  This is slightly misleading though clarified by the keywords.

 The subsections under ‘Results’, also include ‘Recommendations’, e.g. under ‘Health systems: siloed delivery, M&E and supply chain systems', there is a sentence ‘ *Renewed donor efforts are needed to address these health system challenges to integration in order to move toward the provision of integrated services*.’.  Another example is under ‘Limited engagement of the private sector’, the sentence ‘ *Pharmacy provision of PrEP alongside FP could be an avenue for AGYW who can afford it, and may mitigate the stigma they often experience in facilities, although cost will be prohibitive for many AGYW*’, which can also be considered a recommendation.

 Would suggest the Results section be limited to results or findings only, particularly if the recommendations are the opinions of the investigators and the recommendations come within or after the discussion as a separate topic. 

 The discussion itself appears to include some findings from the landscape analysis which could do better in the findings section.

 Integration itself has a very wide scope and when discussing in the conclusions it may be helpful to segregate components better before delving into the specificities of PrEP and SRH.

 Lastly, considering the extremely large number of interviews conducted, findings in the report seem quite limited and it would be interesting to note a little more on the analysis of this sort of qualitative information.

Is the work clearly and accurately presented and does it cite the current literature?

Yes

If applicable, is the statistical analysis and its interpretation appropriate?

Not applicable

Are all the source data underlying the results available to ensure full reproducibility?

Partly

Is the study design appropriate and is the work technically sound?

Yes

Are the conclusions drawn adequately supported by the results?

Yes

Are sufficient details of methods and analysis provided to allow replication by others?

Yes

Reviewer Expertise:

HIV prevention treatment and care; sexual and reproductive health; policy and programming

I confirm that I have read this submission and believe that I have an appropriate level of expertise to confirm that it is of an acceptable scientific standard, however I have significant reservations, as outlined above.

# Response

**Published:** 2022-03-18

**Authors:** Kate Segal

**Affiliations:** AVAC, USA

A very interesting and well-written paper on a topic for which information is needed urgently. The investigations were extensive, especially for the qualitative information gathered.

- ***Author response:*** *Thank you very much.*

However, starting with the title, the initial impression is the integration of HIV response and sexual and reproductive health. The HIV response consists of HIV prevention, treatment, and care, and the paper is focused only on HIV prevention in HIV negatives, PrEP.  This is slightly misleading though clarified by the keywords.

- ***Author response: **We agree with the reviewer’s comment and have made edits throughout the manuscript to clarify the focus on HIV prevention, including in the title, and specifically where we are focusing on oral PrEP and family planning compared to broader HIV prevention and SRH. We changed the phrase “HIV/SRH integration” to “HIV prevention/SRH integration” throughout. We changed the title to Integration of HIV prevention and sexual and reproductive health in the era of anti-retroviral-based prevention: findings from assessments in Kenya, Malawi and Zimbabwe.*

The subsections under ‘Results’, also include ‘Recommendations’, e.g. under ‘Health systems: siloed delivery, M&E and supply chain systems', there is a sentence ‘Renewed donor efforts are needed to address these health system challenges to integration in order to move toward the provision of integrated services.’.  Another example is under ‘Limited engagement of the private sector’, the sentence ‘Pharmacy provision of PrEP alongside FP could be an avenue for AGYW who can afford it, and may mitigate the stigma they often experience in facilities, although cost will be prohibitive for many AGYW’, which can also be considered a recommendation.

- ***Author response: **We agree with the reviewer’s assessment. We moved sentences that are framed as recommendations out of the Results section and, where pertinent, added them in the Discussion section.*

Would suggest the Results section be limited to results or findings only, particularly if the recommendations are the opinions of the investigators and the recommendations come within or after the discussion as a separate topic. 

- ***Author response: **In accordance with the reviewer’s suggestion, we have reorganized the Results section to focus on findings from this analysis, and moved sentences that are interpretations or recommendations to the Discussion section. *

The discussion itself appears to include some findings from the landscape analysis which could do better in the findings section.

- ***Author response: **As part of the reorganization of the Results and Discussion sections, we have moved findings from this analysis to the Results from the Discussion section where appropriate. *

Integration itself has a very wide scope and when discussing in the conclusions it may be helpful to segregate components better before delving into the specificities of PrEP and SRH.

- ***Author response: **Per the reviewer’s suggestion, we have specified in the Conclusions section where we are referring to HIV treatment and SRH, HIV prevention and SRH and specifically to PrEP and other HIV prevention products as components of integration, as follows:* 

*"While the call for HIV/SRH integration is not new, it has been implemented more successfully for HIV treatment and SRH, while integration of HIV prevention with SRH has lagged. Although integrated policies exist on paper, HIV prevention and SRH services remain siloed and these policies preceded the introduction of new biomedical interventions such as oral PrEP. With the introduction of HIV prevention products expected in the next couple of years, there is an urgency to ensure that policies on integration are updated and that programs build on lessons where HIV prevention and SRH integration has been successful. In Kenya, Malawi and Zimbabwe, YFHS and PMTCT programs offer the strongest examples of integration of HIV prevention and SRH that can be leveraged and expanded to more effectively reach AGYW."*

 Lastly, considering the extremely large number of interviews conducted, findings in the report seem quite limited and it would be interesting to note a little more on the analysis of this sort of qualitative information.

- ***Author response: **In accordance with the reviewer’s suggestion, we have added information to describe the methodology and qualitative analysis process in greater detail. The Methods section now begins with the following introduction:*

*"This article is a comparative analysis of findings from three completed rapid health systems and landscaping assessments of the integration of HIV prevention, including oral PrEP, in SRH services in Kenya, Malawi and Zimbabwe. These rapid assessments utilized proven methodologies for rapid assessments, building from WHO and Measure Evaluation’s “Routine Health Information System Rapid Assessment Tool.” The assessments collected programmatic and clinic data from public health facilities and NGO-supported programs, and gathered insights from Ministry of Health (MOH) officials, providers, NGO staff and others working at district/county levels, and employed a thematic analysis of data generated, with the intent to rapidly inform national policies and programs. By synthesizing, analyzing and contextualizing the findings of the three assessments, this article aims to understand the current state of HIV prevention/SRH integration in each country and to elucidate cross-country trends. Summaries of and quotes from primary qualitative data from the rapid assessments were included in this article to underscore findings, and outside literature was incorporated to provide additional context. Because the rapid assessments comprise the main source material of this article, their methodologies are elaborated upon below."*

- *In addition, we updated the section Data analysis for rapid assessments to include more information:*

*"PMM used standardized interview guides and health facility assessment checklists to conduct the rapid assessment and analysis of data across Kenya and Zimbabwe. The research teams compiled detailed notes after each interview using a standard template, referencing recorded interviews to ensure accuracy and attention to detail, and to document supporting quotes. Health facility assessments collected quantitative data on HIV, FP/SRH and YF services available (e.g., methods and hours offered, whether and how services are integrated, commodity stock-outs, YF accommodations) and staffing and general infrastructure (e.g., facility size, number and cadres of staff across HIV, SRH and YF service areas) to analyze the level of HIV prevention/SRH integration and YF service delivery at each facility. To understand opportunities for and barriers to HIV prevention/SRH integration, each team conducted thematic analysis of detailed notes through a workshop once data collection was completed to identify common themes and outlier perspectives across the interviews, grouped by stakeholder perspectives at the government, health facility and community levels. Findings were triangulated across qualitative and quantitative data.*

*To understand the spectrum of services in the Blantyre district, the research team analyzed HIV and SRH information and services offered through public sector facilities and youth-friendly health services (YFHS), as well as in the private sector, including national and international NGOs and NGOs with the U.S.-led DREAMS initiative. The analysis highlighted social and structural barriers for sustained and effective use of prevention interventions, including through the education system and gender-based violence (GBV) programs, and new and evolving challenges presented by the COVID-19 pandemic."*
